# The health burden of disease attributable to low calcium intake: a comprehensive analysis of trends and socioeconomic impacts from 1990 to 2021

**DOI:** 10.3389/fnut.2025.1594656

**Published:** 2025-07-09

**Authors:** Yanping Wang, Meihui Tao, Li Wang, Siyu Zhou, Weifeng Yang, Xi Zhao, Qinyu Feng, Mengfan Tang, Wei Yan, Yu Fu

**Affiliations:** ^1^Department of Gastroenterology, Union Hospital, Tongji Medical College, Huazhong University of Science and Technology, Wuhan, China; ^2^Department of Emergency Surgery, Union Hospital, Tongji Medical College, Huazhong University of Science and Technology, Wuhan, China; ^3^Department of Gastrointestinal Surgery, The Affiliated Hospital, Southwest Medical University, Luzhou, China; ^4^Department of Gastroenterology, Tongji Hospital, Tongji Medical College, Huazhong University of Science and Technology, Wuhan, China

**Keywords:** global burden of disease, human development index, socio-demographic index, diet low in calcium, colon and rectum cancer, prostate cancer

## Abstract

**Background:**

Calcium, a vital nutrient for the human body, is indispensable for keeping our bones strong and managing cell function. A diet low in calcium (DLC) is a key player in the formation of numerous health issues. This research delved into the most recent datasets acquired via the 2021 Global Burden of Disease (GBD) report to uncover the worldwide impact of DLC.

**Methods:**

Utilizing the GBD 2021 database, this research examined the association of DLC with disease burden, covering colorectal and prostate cancers. To quantify disease burden and track its temporal variations, key indicators were employed. These included deaths, disability-adjusted life years (DALYs), age-standardized mortality rate (ASMR), age-standardized DALY rate (ASDR), and the estimated annual percentage change (EAPC). The analysis broke down results by sex, age brackets, Socio-demographic Index (SDI) categories, and geographic regions. To examine potential links between disease burden and socioeconomic factors, Pearson's correlation method was applied. Furthermore, Bayesian age-period-cohort (BAPC) modeling was applied to forecast trends spanning 2022–2050.

**Results:**

From 1990 to 2021, global colon and rectum cancer (CRC)-related deaths tied to DLC rose from 57,363 to 89,089, while the ASMR decreased from 1.54 to 1.06 per 100,000 [EAPC = −1.33 (95% CI: −1.37 to −1.29)]. DALYs increased from 1,512,762 to 2,128,939, and the ASDR declined from 37.04 to 24.7 per 100,000 [EAPC = −1.45 (95% CI: −1.50 to −1.40)]. The burden of CRC tended to escalate with age, and it disproportionately affected women more than men. However, the impact on women diminished more rapidly over time. Prostate cancer and DLC showed a negative directional trend but lacked statistical significance. CRC burden showed a negative correlation with SDI, while prostate cancer burden had a positive association.

**Conclusion:**

DLC significantly increases the incidence of CRC, while its impact on prostate cancer incidence is relatively minor and shows a negative correlation. Both phenomena are closely associated with socioeconomic development. The research yields essential data to develop focused dietary interventions and cancer-prevention policies.

## Introduction

The interplay of nutritional factors with cancer has long been a key area of study in worldwide public health investigations. As a vital nutrient, calcium does more than just support bone strength, it may also impact cancer development by modulating critical cellular functions like proliferation, differentiation, and programmed cell death (1, 2). Dietary intake serves as the primary source of calcium for humans. However, influenced by socioeconomic status, dietary habits, and food diversity, inadequate calcium intake may lead to calcium deficiency-related diseases and impose a significant burden on public health (3).

Accumulating evidence indicates that calcium intake may confer a protective effect against CRC (4). A well-established inverse correlation exists for calcium consumption amounts in relation to CRC risk, as evidenced by an extensive meta-analysis indicating that a daily 300 mg rise in calcium consumption corresponds to an 8% decrease in the risk of CRC (RR = 0.92; 95% CI: 0.89 to 0.95) (5). Calcium is central to preventing colorectal cancer, as it binds to bile acids, fatty acids, and heme iron in the gut. This interaction helps neutralize their irritating effects on the colonic mucosa, ultimately lowering the chances of developing CRC (6, 7). Furthermore, calcium has been shown to modulate critical cellular processes, including proliferation and differentiation, effectively suppressing tumor cell growth and metastasis (7, 8). Despite these protective effects, global calcium intake patterns exhibit substantial geographical variations due to diverse dietary practices and nutritional disparities (9). Particularly in regions with suboptimal calcium intake, this nutritional deficiency may contribute to elevated CRC incidence rates (3). Several research findings suggest a possible rise in the likelihood of prostate cancer with elevated consumption of total calcium, calcium from diet, and calcium from dairy sources (10). Research indicates a positive correlation of calcium consumption with prostate cancer. The higher the calcium consumption, the lower the 1,25(OH)2 vitamin D concentration (11). As the most biologically active form of vitamin D, 1,25(OH)2 vitamin D plays a critical role in calcium and phosphorus metabolism. It elevates calcium concentrations by acting on bones, kidneys, and the intestine when circulating calcium levels are low. Conversely, elevated blood calcium suppresses parathyroid hormone secretion, thereby inhibiting 1,25(OH)2 vitamin D production (12). 1,25(OH)2 vitamin D can suppress tumor cell growth, invasion, and spread while prompting differentiation and cell death through control of gene expression (10, 13). However, high calcium intake could undermine the cancer-fighting prowess of 1,25(OH)2 vitamin D by lowering circulating concentration, potentially speeding up tumor growth. Moreover, studies indicate that this process might heighten the likelihood of developing prostate cancer (14).

Although prior research evaluated the disease burden linked to CRC, there remains a significant gap in the comprehensive and in-depth analysis regarding the specific impact of DLC as a standalone risk factor on CRC burden, particularly in terms of its dynamic trends over an extended time span (1990–2021). Moreover, comprehensive studies on low calcium intake's link to prostate cancer burden remain scarce. Drawing on comprehensive findings from the 2021 GBD research, this investigation thoroughly examines the burden exerted by colorectal and prostate cancers linked to DLC from 1990 through 2021. By analyzing trends at different geographical levels, the study sheds light on critical public health concerns stemming from this nutritional deficiency. These insights not only highlight pressing health challenges but also establish a scientific base for crafting specific nutritional intervention programs and cancer mitigation tactics.

## Materials and methods

### Data source

Our research data is directly obtained from the 2021 GBD report. This academic endeavor is thorough in scope. It seeks to measure the burden impacts from numerous diseases, injuries, and associated risks. Age, gender, and regional factors are all considered during the analysis (15). The 2021 GBD study carried out a systematic evaluation of disease burden. It covered 371 diseases and injuries, together with 88 risk factors. The assessment was conducted across 204 countries and regions worldwide (16). Notably, this paper leverages GBD 2021 data to systematically analyze the population health impacts of DLC from 1990 to 2021. Employing standardized methodologies, we conducted a comprehensive analysis that encompassed age groups, genders, geographical regions, and temporal dimensions. The research specifically evaluated deaths and DALYs linked to DLC and calculated the corresponding ASMR and ASDR. All data were obtained from the official GBD database, accessible to researchers at https://vizhub.healthdata.org/gbd-results/. To ensure methodological rigor, the data analysis strictly adhered to the standard operating procedures of the GBD study and incorporated multiple statistical approaches for robust data interpretation.

### Definitions

According to GBD 2021, DLC refers to a dietary risk factor characterized by a total calcium intake from various sources, including milk, yogurt, and cheese, which is below 0.72–0.86 grams per day for males and below 1.1–1.2 grams per day for females. DALYs serve as a measure for quantifying the overall disease burden. This measure works by estimating the count of healthy years that are lost. Such losses can be attributed to various factors, including illness, disability, or premature death (17). These calculations rely on standardized global population data from the GBD study, incorporating a 95% uncertainty range to reflect potential variations in the underlying data. As a comprehensive measure that integrates both the quantity and quality of life, DALYs are broadly acknowledged as a highly efficient means for evaluating the worldwide disease burden (18). Additionally, this research employs the EAPC for measuring trends in ASMR and ASDR. Notably, a key metric for assessing a nation's overall socioeconomic progress, the SDI is categorized into five levels: high, high-middle, middle, low-middle, and low (19). For the 2021 Human Development Index (HDI), data were retrieved from the database maintained by the United Nations Development Programme. This database can be accessed via the link: https://hdr.undp.org/data-center/human-development-index. As a key measure of comprehensive socioeconomic development, the HDI takes into account three critical factors: life expectancy, educational attainment, and national income. Scaled from 0 to 1, with values closer to 1 representing more advanced development (20).

### Estimates

The 2021 GBD study conducted an exhaustive assessment of 50 different cancers across 204 nations and regions worldwide. Cancer registries and vital registration systems served as the main sources of mortality data, which were analyzed through the Cause of Death Ensemble modeling (CODEm) methodology. This framework employs a range of rate- or cause-specific modeling techniques to ensure accurate estimation and analysis of cause-specific mortality data (21). Moreover, DALYs offer a holistic measure of total health burden by combining two key components: years of life lost (YLLs) and years lived with disability (YLDs) for each specific health issue. DALYs provide a holistic assessment of disease burden by quantifying both prematurely lost life-years and health loss attributable to disease-related disability, thereby establishing a robust framework for evaluating population health impact (22).

### Statistical analysis

We stratified the data by gender, age categories, SDI, geographic region, and nation to obtain ASMR and ASDR. To guarantee uniformity and cross-source comparability of the data, the ASR underwent age-standardization using populations normalized to global norms. To gauge the shifts in ASMR and ASDR from 1990 up until 2021, we employed the EAPC. To derive the 95% CI for the final estimates, 1,000 random resamples were drawn from the estimation distribution. The lower and upper CI thresholds were ascertained by identifying the 2.5th and 97.5th percentile values of this resampled distribution (23). The EAPC was derived via log-linear regression to evaluate yearly shifts in disease burden indicators. The statistical model was structured as *y* = α + β*x* + ε, with *y* standing for the log-transformed ASR values, *x* corresponding to the year of observation, ε being the random error term, and β representing the temporal trend of ASR. To estimate the EAPC, we calculated it directly from the regression coefficient (β) through the expression: EAPC = 100 × [exp(β) – 1]. Trends were analyzed using 95% confidence intervals: an upward trend was noted when the lower limit of the interval was above zero, a downward trend when the upper bound fell below zero, and no meaningful trend if the CI enveloped zero (24, 25). Pearson correlation analysis assessed the links of EAPC with 1990 ASRs and 2021 HDI. To project new CRC cases and ASRs linked to DLC up to 2050, we employed a log-linear age-period-cohort model, which captures exponential growth patterns. Projections were generated with the BAPC package in R software, incorporating population forecasts from the United Nations for each country (26). All statistical analyses were performed using R software (version 4.1.2), and a significance level of *P* < 0.05 was employed for statistical testing.

## Results

### Global level

**Figure 1:**
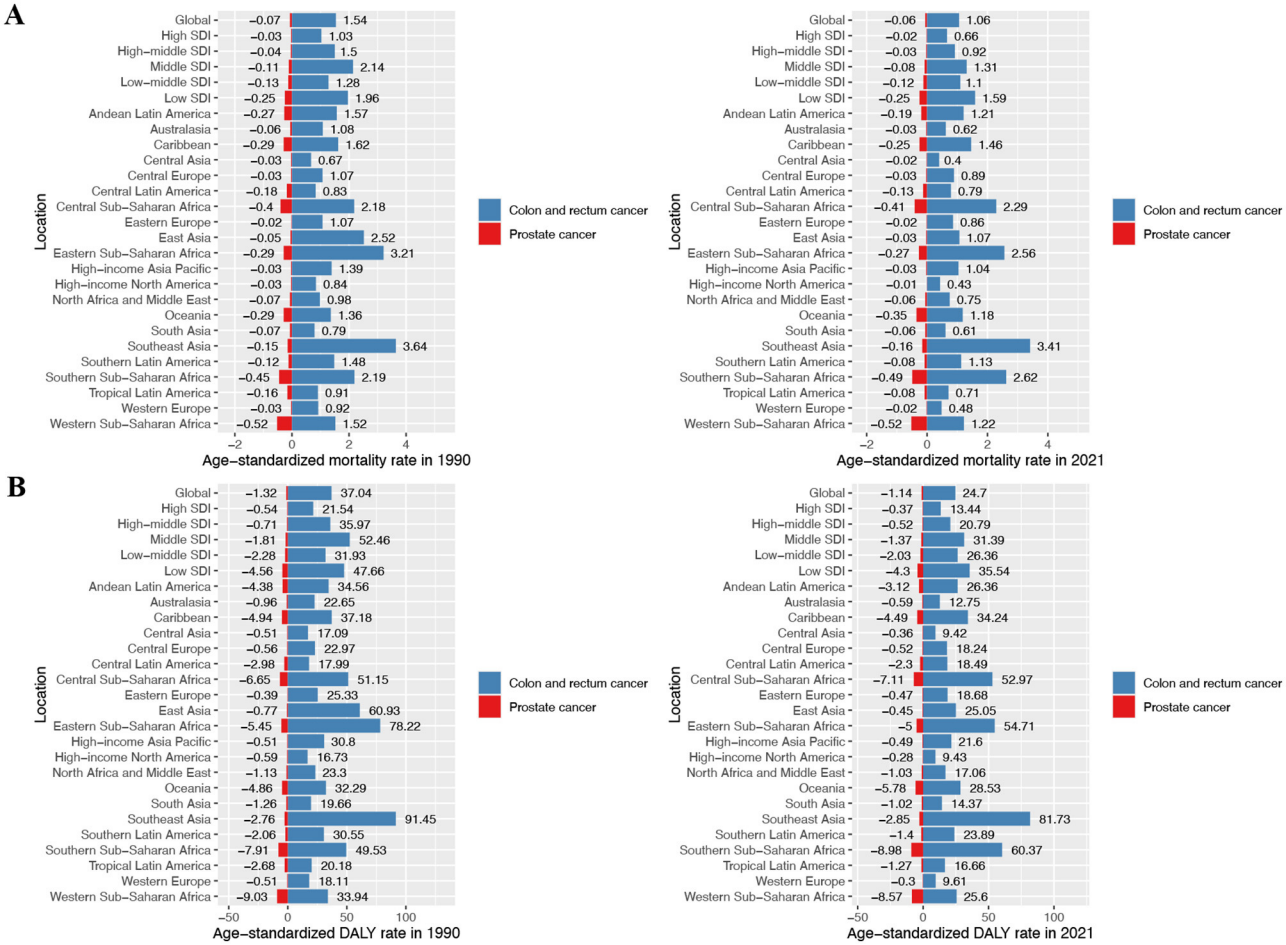
ASMR and ASDR of prostate cancer and colon and rectum cancer attributable to diet low in calcium in different regions and global average levels in 1990 and 2021. **(A)** Prostate cancer and colon and rectum cancer age-standardized mortality rate (ASMR) in 1990 and 2021; **(B)** prostate cancer and colon and rectum cancer age-standardized DALY rate (ASDR) in 1990 and 2021.

Diseases associated with DLC include colorectal and prostate cancers. Globally, CRC deaths attributable to DLC rose from 57,363 cases (95% UI: 42,915 to 71,257) in 1990 to 89,089 cases (95% UI: 65,019 to 112,298) in 2021. Nonetheless, the ASMR showed a decline from 1.54 (95% UI: 1.15 to 1.92) to 1.06 (95% UI: 0.77 to 1.33) per 100,000 population, corresponding to an EAPC of −1.33 (95% CI: −1.37 to −1.29) (Figure 1A; Table 1). Similarly, global DALYs attributable to DLC increased from 1,512,762 (95% UI: 1,132,026 to 1,881,667) in 1990 to 2,128,939 (95% UI: 1,565,530 to 2,672,450) in 2021. The ASDR declined from 37.04 (95% UI: 27.74 to 46.14) to 24.7 (95% UI: 18.17 to 31.02) per 100,000 population, corresponding to an EAPC of −1.45 (95% CI: −1.50 to −1.40) (Figure 1A; Table 1). The absolute counts of deaths and DALYs showed an upward trajectory. Conversely, both the ASMR and ASDR demonstrated a continuous decline throughout the investigative span.

**Table 1:** The deaths cases, age-standardized deaths, or temporal trends of the burden of disease attributable to diet low in calcium in 204 countries, 1990 and 2021.

**Disease**	**1990**				**2021**				**EAPC (1990–2021)**	
	**Deaths cases No. (95% UI)**	**ASMR per 100,000 No. (95% UI)**	**DALYs No. (95% UI)**	**ASDR per 100,000 No. (95% UI)**	**Deaths cases No. (95% UI)**	**ASMR per 100,000 No. (95% UI)**	**DALYs No. (95% UI)**	**ASDR per 100,000 No. (95% UI)**	**ASMR No. (95% CI)**	**ASDR No. (95% CI)**
**Colon and rectum cancer**										
Global	57,363 (42,915, 71,257)	1.54 (1.15, 1.92)	1,512,762 (1,132,026, 1,881,667)	37.04 (27.74, 46.14)	89,089 (65,019, 112,298)	1.06 (0.77, 1.33)	2,128,939 (1,565,530, 2,672,450)	24.7 (18.17, 31.02)	−1.33 (−1.37, −1.29)	−1.45 (−1.5, −1.4)
Female	33,956 (24,765, 42,701)	1.65 (1.20, 2.08)	855,358 (617,926, 1,075,506)	39.69 (28.74, 49.84)	52,121 (37,283, 66,521)	1.13 (0.81, 1.44)	1,189,269 (865,714, 1,502,083)	26.13 (19.01, 32.99)	−1.37 (−1.42, −1.32)	−1.51 (−1.57, −1.45)
Male	23,407 (17,131, 30,625)	1.37 (1.00, 1.80)	657,404 (478,504, 854,471)	33.79 (24.47, 44.07)	36,968 (26,842, 49,073)	0.96(0.69,1.27)	939,669 (680,055, 1,241,878)	22.95 (16.61, 30.33)	−1.26 (−1.32, −1.2)	−1.36 (−1.41, −1.31)
**Prostate cancer**										
Global	−2,497 (−5,569, 461)	−0.07 (−0.16,0.01)	−48,417 (−10,8244, 8,987)	−1.32 (−2.93,0.24)	−5,225 (−11,326, 983)	−0.06 (−0.14,0.01)	−97,461 (−21,1240, 18,435)	−1.14 (−2.48,0.22)	–	–

ASMR, age-standardized mortality rate; DALYs, disability-adjusted life-years; ASDR, age-standardized DALY rate; UI, uncertainty interval; EAPC, estimated annual percentage change; CI, confidence interval.

Regarding prostate cancer, the global deaths due to DLC shifted from −2,497 cases (95% UI: −5,569 to 461) in 1990 to −5,225 cases (95% UI: −11,326 to 983) in 2021. Concurrently, the ASMR changed from −0.07 (95% UI: −0.16 to 0.01) to −0.06 (95% UI: −0.14 to 0.01) per 100,000 population over this identical timeframe (Figure 1A; Table 1). Furthermore, global DALYs associated with DLC increased from −48,417 (95% UI: −108,244 to 8,987) in 1990 to −97,461 (95% UI: −211,240 to 18,435) in 2021. The ASDR also shifted from −1.32 (95% UI: −2.93 to 0.24) to −1.14 (95% UI: −2.48 to 0.22) per 100,000 population over the study period (Figure 1A; Table 1).

### National level

**Figure 2:**
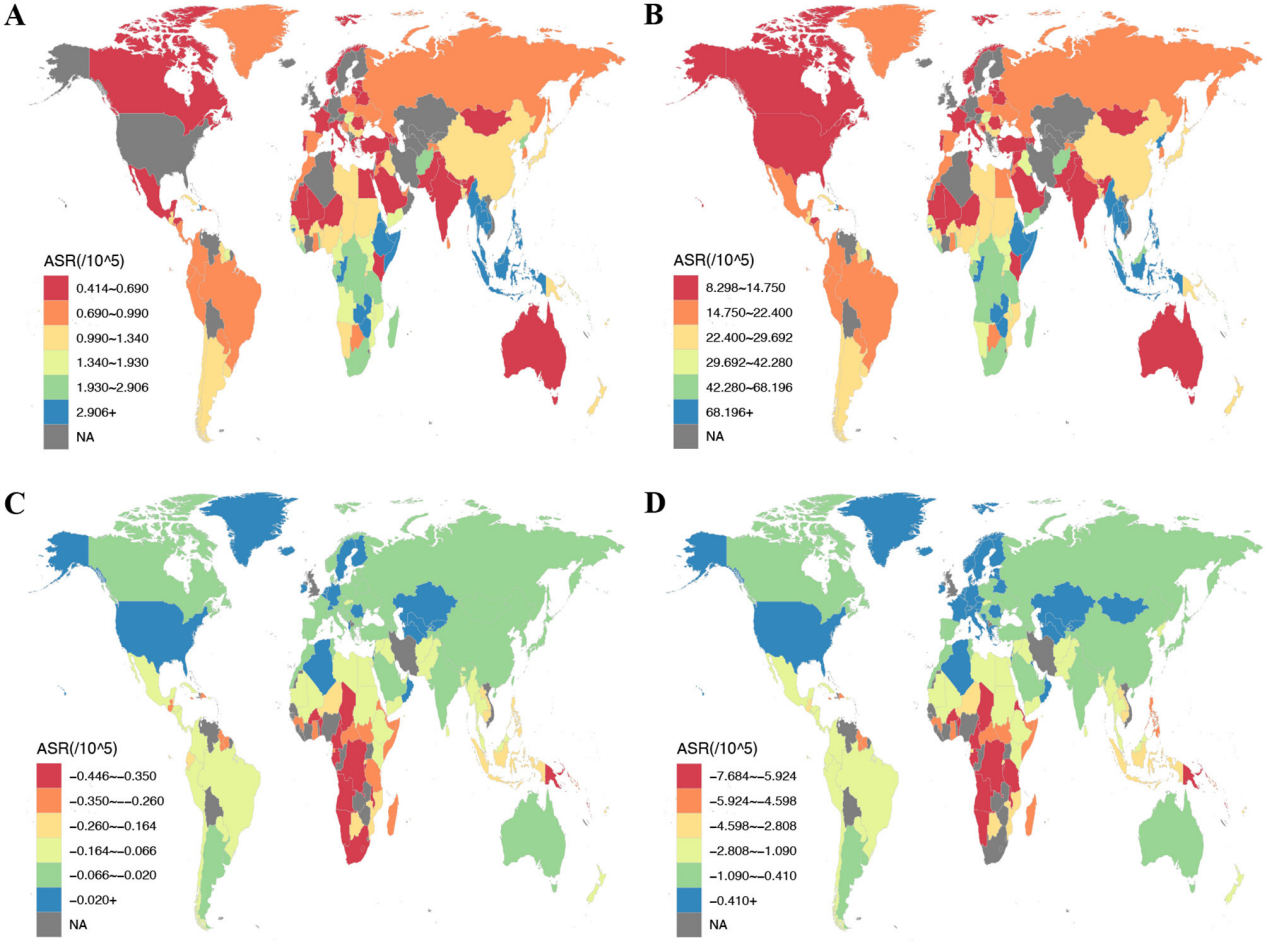
The ASR of deaths and DALYs attributable to diet low in calcium for prostate cancer and colon and rectum cancer among countries and territories in 2021. **(A)** ASMR of colon and rectum cancer; **(B)** ASDR of colon and rectum cancer; **(C)** ASMR of prostate cancer; **(D)** ASDR of prostate cancer. ASMR, age-standardized mortality rate; ASDR, age-standardized DALY rate; DALYs, disability-adjusted life year rates.

Of the 204 nations assessed, China experienced the peak CRC burden from DLC in 2021, with 20,719 deaths (95% UI: 14,553 to 28,272) and 500,468 DALYs (95% UI: 356,219 to 682,873), respectively. In contrast, Zambia led in ASMR and ASDR at 5.17 (95% UI: 3.3 to 9.53) and 123.81 (95% UI: 74.17 to 249.01) per 100,000 population, respectively. By contrast, Albania had minimal ASMR and ASDR: 0.18 (95% UI: 0.1 to 0.28) and 3.54 (95% UI: 2.09 to 5.66) per 100,000 population in sequence (Figure 2; Supplementary Tables S1, S2). Over the 1990–2021 period, the United Arab Emirates experienced the greatest rise in CRC death rate linked to DLC, with an EAPC of 2.98 (95% CI: 2.2 to 3.75). Likewise, Lesotho showed the largest increase in CRC DALY rate, with EAPC measuring 2.97 (95% CI: 2.48 to 3.46). Conversely, Maldives exhibited the steepest reductions in both ASMR and ASDR, featuring EAPCs of −4.82 (95% CI: −5.1 to −4.53) and −5.54 (95% CI: −5.88 to −5.19), respectively (Supplementary Figure S1, Supplementary Tables S1, S2).

In 2021, for prostate cancer, China remained the country with the largest reduction in deaths attributable to DLC, while India showed the greatest reduction in DALYs, with −496 deaths (95% UI: −1,164 to 92) and −8,833 DALYs (95% UI: −19,586 to 1,535), respectively. Zimbabwe recorded maximal ASMR and ASDR reductions: −0.98 (95% UI: −2.19 to 0.17) and −17.89 (95% UI: −39.66 to 3.18) per 100,000 population. In contrast, Kazakhstan demonstrated the smallest reduction in ASDR, at −0.11 (95% UI: −0.27 to 0.02) per 100,000 population (Figure 2; Supplementary Tables S3, S4).

### GBD regional level

During 2021, East Asia bore the greatest CRC deaths among GBD regions due to DLC, while Southeast Asia exhibited the greatest burden in terms of DALYs, with 22,174 deaths (95% UI: 15,483 to 29,949) and 560,315 DALYs (95% UI: 418,460 to 695,240), respectively. Additionally, Southeast Asia maintained the greatest ASMR and ASDR, at 3.41 (95% UI: 2.56 to 4.23) and 81.73 (95% UI: 61.48 to 101.51) per 100,000 population, respectively (Figure 1A; Supplementary Tables S5, S6). In contrast, in 2021, Oceania recorded minimal CRC burden from DLC, specifically 80 deaths (95% UI: 57 to 100) and 2,413 DALYs (95% UI: 1,738 to 3,087). Meanwhile, Central Asia demonstrated the lowest ASMR and ASDR, at 0.40 (95% UI: 0.28 to 0.52) and 9.42 (95% UI: 6.49 to 12.56) per 100,000 population, respectively (Figure 1A; Supplementary Tables S5, S6). From 1990 to 2021, East Asia experienced the sharpest drops in CRC mortality and DALY rate associated with DLC, with EAPC of −2.90 (95% CI: −2.99 to −2.80) and −3.01 (95% CI: −3.11 to −2.92), respectively. Conversely, Southern Sub-Saharan Africa showed the largest increases, with EAPC of 0.57 (95% CI: 0.27 to 0.87) for mortality and 0.72 (95% CI: 0.40 to 1.04) for DALY rate (Supplementary Tables S5, S6).

For prostate cancer in 2021, Southeast Asia exhibited the largest shifts in deaths and DALYs from DLC, with −839 deaths (95% UI: −1,769 to 162) and −16,744 DALYs (95% UI: −35,590 to 3,213). In contrast, Central Asia showed the smallest changes, with −13 deaths (95% UI: −29 to 2) and −268 DALYs (95% UI: −609 to 52). ASMR and ASDR declines peaked in Western and Southern Sub-Saharan Africa, respectively: ASMR at −0.52 (95% UI: −1.19 to 0.11) and ASDR at −8.98 (95% UI: −19.64 to 1.69) per 100,000 population. In contrast, High-income North America demonstrated the least significant for ASMR and ASDR: −0.01 (95% UI: −0.03 to 0) and −0.28 (95% UI: −0.64 to 0.05) per 100,000 population, respectively (Figure 1B; Supplementary Tables S7, S8).

### SDI regional level

In 2021, the Middle SDI region had the most CRC deaths and DALYs linked to DLC among the five SDI regions, with 33,662 deaths (95% UI: 25,076 to 42,327) and 860,241 DALYs (95% UI: 640,404 to 1,080,803), respectively. Conversely, Among all regions, Low SDI demonstrated minimal values, with 7,039 deaths (95% UI: 5,238 to 8,698) and 189,763 DALYs (95% UI: 140,383 to 235,803). However, the Low SDI region demonstrated the greatest ASMR and ASDR, at 1.59 (95% UI: 1.18 to 1.97) and 35.54 (95% UI: 26.48 to 43.95) per 100,000 population, respectively. Inversely, the High SDI region reported the lowest ASMR and ASDR, at 0.66 (95% UI: 0.45 to 0.87) and 13.44 (95% UI: 9.33 to 17.88) per 100,000 population, respectively (Figure 1; Supplementary Figure S2A; Supplementary Tables S5, S6). Spanning 1990–2021, all socioeconomic development strata demonstrated reductions in both ASMR and ASDR for CRC linked to DLC. The High-middle SDI region experienced the most significant declines, with EAPC of −1.74 (95% CI: −1.83 to −1.65) for ASMR and −1.98 (95% CI: −2.07 to −1.89) for ASDR. Conversely, the Low-middle SDI region showed the smallest declines, with EAPC of −0.57 (95% CI: −0.65 to −0.50) for ASMR and −0.72 (95% CI: −0.80 to −0.65) for ASDR (Supplementary Figure S2A; Supplementary Tables S5, S6).

Within the Middle SDI region in 2021, prostate cancer burden linked to DLC demonstrated the most pronounced declines, specifically −1,837 deaths (95% UI: −3,960 to 348) and −34,282 DALYs (95% UI: −73,402 to 6,497). Comparatively, the High SDI region exhibited minimal changes, with −522 deaths (95% UI: −1,183 to 92) and −8,543 DALYs (95% UI: −19,103 to 1,547). For age-standardized rates, the most substantial burden reductions occurred in the Low SDI region, with ASMR at −0.25 (95% UI: −0.55 to 0.05) and ASDR at −4.30 (95% UI: −9.68 to 0.83) per 100,000 population. By contrast, the High SDI region displayed minimal burden variations: ASMR declined by −0.02 (95% UI: −0.05 to 0) and ASDR by −0.37 (95% UI: −0.82 to 0.07) per 100,000 population (Figure 1; Supplementary Figure S2A; Supplementary Tables S7, S8). From 1990 to 2021, No statistically detectable variations emerged in DLC-linked prostate cancer mortality or DALY rates across the five SDI regions (Supplementary Figure S2B).

### Gender patterns

Globally, a higher CRC burden linked to DLC was observed in women than in men. In 2021, females exhibited higher CRC deaths and DALYs associated with DLC compared with males, with 52,121 deaths (95% UI: 37,283 to 66,521) and 1,189,269 DALYs (95% UI: 865,714 to 1,502,083) observed in females, respectively (Table 1). Similarly, females exhibited higher ASMR and ASDR than males in 2021, specifically 1.13 (95% UI: 0.81 to 1.44) and 26.13 (95% UI: 19.01 to 32.99) per 100,000 population for these two measures (Table 1). During 1990–2021, the CRC burden associated with DLC declined more rapidly in women than in men. Specifically, the EAPC for ASMR in females was −1.37 (95% CI: −1.42 to −1.32), while the EAPC for ASDR was −1.51 (95% CI: −1.57 to −1.45) (Table 1).

In 2021, CRC deaths and DALYs linked to DLC among males were 36,968 (95% UI: 26,842 to 49,073) and 939,669 (95% UI: 680,055 to 1,241,878), respectively (Table 1). The ASMR and ASDR for males were 0.96 (95% UI: 0.69 to 1.27) and 22.95 (95% UI: 16.61 to 30.33) per 100,000 population, respectively. Over the 1990–2021 period, males exhibited EAPCs of −1.26 (95% CI: −1.32 to −1.20) for ASMR and −1.36 (95% CI: −1.41 to −1.31) for ASDR (Table 1).

### Age patterns

**Figure 3:**
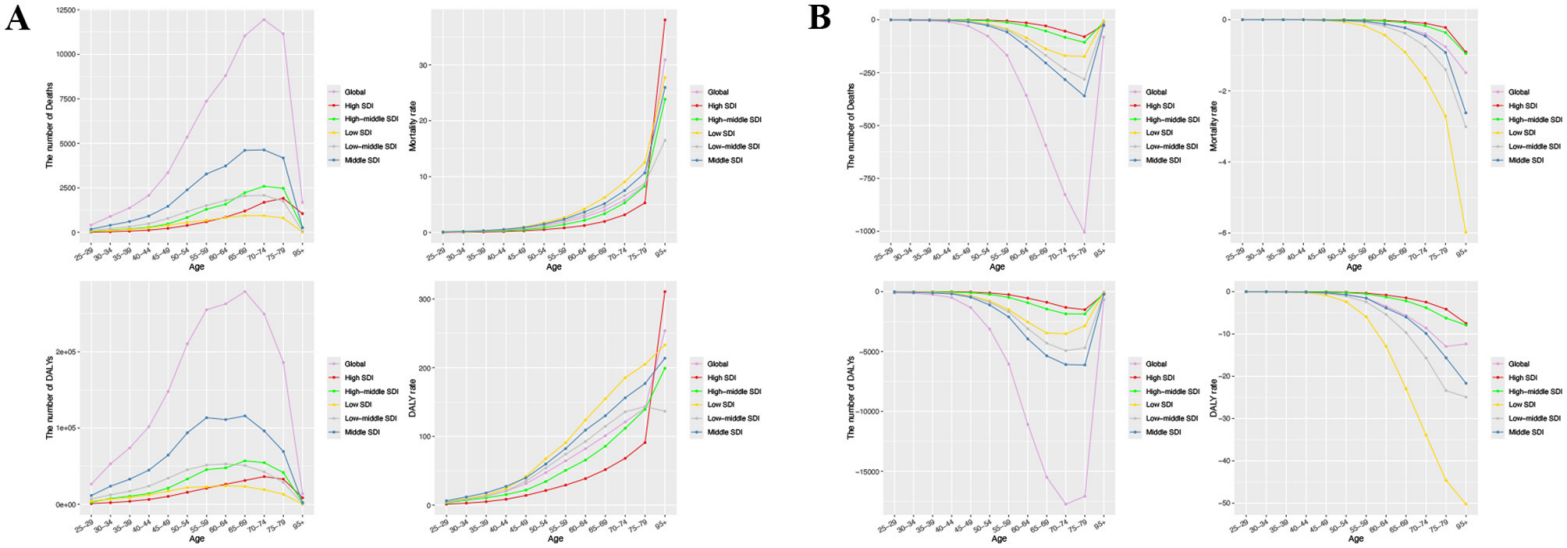
The number and rate of deaths and DALYs attributable to diet low in calcium for prostate cancer and colon and rectum cancer by age group among global and different SDI regions in 2021. **(A)** Colon and rectum cancer; **(B)** Prostate cancer. DALYs, disability-adjusted life year rates; SDI, sociodemographic index.

In 2021, global CRC deaths linked to DLC peaked among 70–74-year-olds, whereas DALYs peaked at their maximum in individuals aged 65–69 years (Figure 3A). Furthermore, CRC's ASMR and ASDR due to DLC increased with age (Figure 3A).

For prostate cancer, the most substantial shifts in deaths and DALYs linked to DLC in 2021 were noted in the 75–79 and 70–74 age brackets, in that order (Figure 3B). Importantly, prostate cancer mortality and DALY rates linked to DLC declined as age advanced, particularly in regions with low SDI (Figure 3B).

### Factors linked to disease burden from DLC

**Figure 4:**
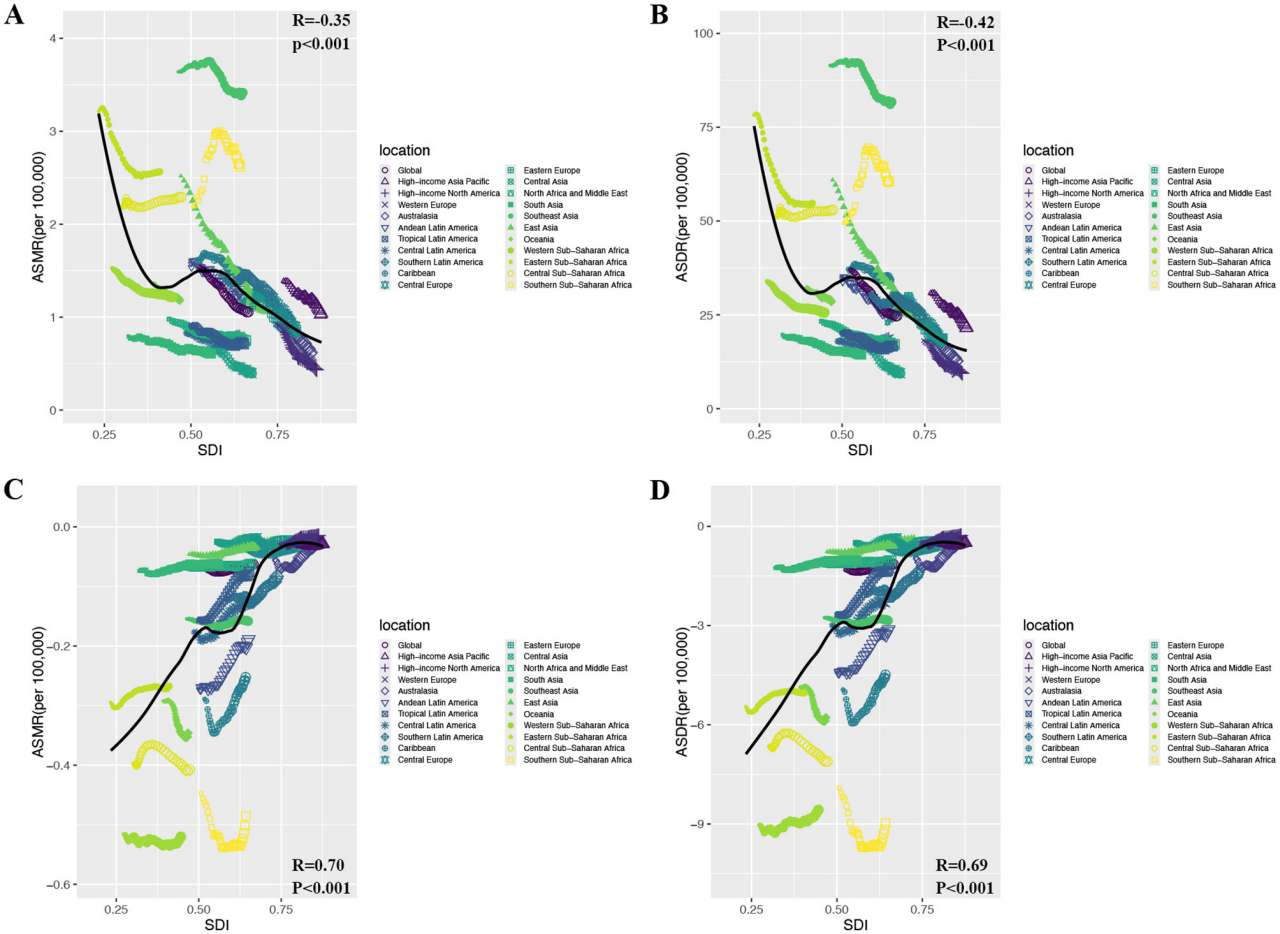
The relationship between ASMR/ASDR attributable to diet low in calcium and SDI for prostate cancer and colon and rectum cancer in 2021. **(A)** ASMR of colon and rectum cancer; **(B)** ASDR of colon and rectum cancer; **(C)** ASMR of prostate cancer; **(D)** ASDR of prostate cancer. ASMR, age-standardized mortality rate; ASDR, age-standardized DALY rate; DALYs, disability-adjusted life year rates; SDI, sociodemographic index.

This research examined the links of SDI with ASMR/ASDR for Colorectal and prostate cancer due to DLC. It encompassed 204 countries and 21 regions in 2021, assessing the relationship of SDI to DLC-related cancer burden metrics (Figure 4; Supplementary Figure S3). Overall, among the 21 regions, as SDI increased, CRC's ASMR and ASDR showed an inverse trend relative to SDI. (ASMR: *R* = −0.35, *P* < 0.001; ASDR: *R* = −0.42, *P* < 0.001) (Figures 4A, B). Notably, in absolute terms, prostate cancer's ASMR and ASDR correlated negatively with SDI. (ASMR: *R* = 0.70, *P* < 0.001; ASDR: *R* = 0.69, *P* < 0.001) (Figures 4C, D). Furthermore, among the 204 countries, we observed that CRC's ASMR and ASDR initially showed a slight decline, followed by a modest increase, and then a subsequent decline with increasing SDI, ultimately demonstrating an overall negative correlation (ASMR: *R* = −0.56, *P* < 0.001; ASDR: *R* = −0.60, *P* < 0.001) (Supplementary Figures S3A, B). Conversely, the absolute magnitudes of prostate cancer's ASMR and ASDR first increased, then decreased as SDI rose, showing an overall positive correlation. (ASMR: *R* = 0.70, *P* < 0.001; ASDR: *R* = 0.70, *P* < 0.001) (Supplementary Figures S3C, D). These findings indicate that regions and nations with higher SDI saw a notable decline in CRC burden linked to DLC, whereas prostate cancer showed a marked increase.

**Figure 5:**
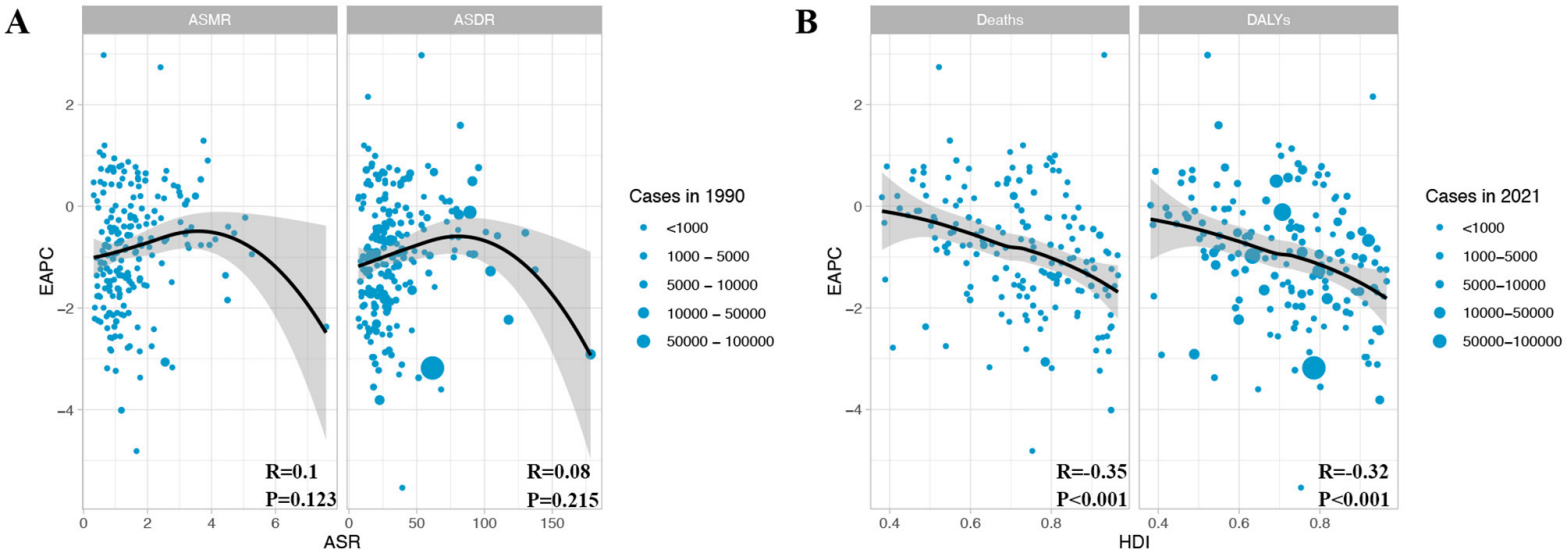
The relationship between EAPC with ASMR/ASDR and HDI in colon and rectum cancer. **(A)** EAPC and ASMR/ASDR in 1990; **(B)** EAPC and HDI in 2021. EAPC, estimated annual percentage change; ASMR, age-standardized mortality rate; ASDR, age-standardized DALY rate; DALYs, disability-adjusted life year rates; HDI, human development index.

Next, we examined the associations of the EAPC for CRC attributable to DLC with both ASMR and ASDR in 1990, as well as the association of EAPC with the HDI in 2021 (Figure 5). We found no significant correlation between EAPC and ASR (ASMR: *R* = 0.1, *P* = 0.123; ASDR: *R* = 0.08, *P* = 0.215) (Figure 5A). However, there was a considerable inverse relationship between the EAPC and the HDI regarding both deaths and DALYs (Deaths: *R* = −0.35, *P* < 0.001; DALYs: *R* = −0.32, *P* < 0.001) (Figure 5B). These results indicate that countries with elevated HDI levels saw a more pronounced decline in CRC burden due to DLC.

### Projected CRC burden due to DLC trends

**Figure 6:**
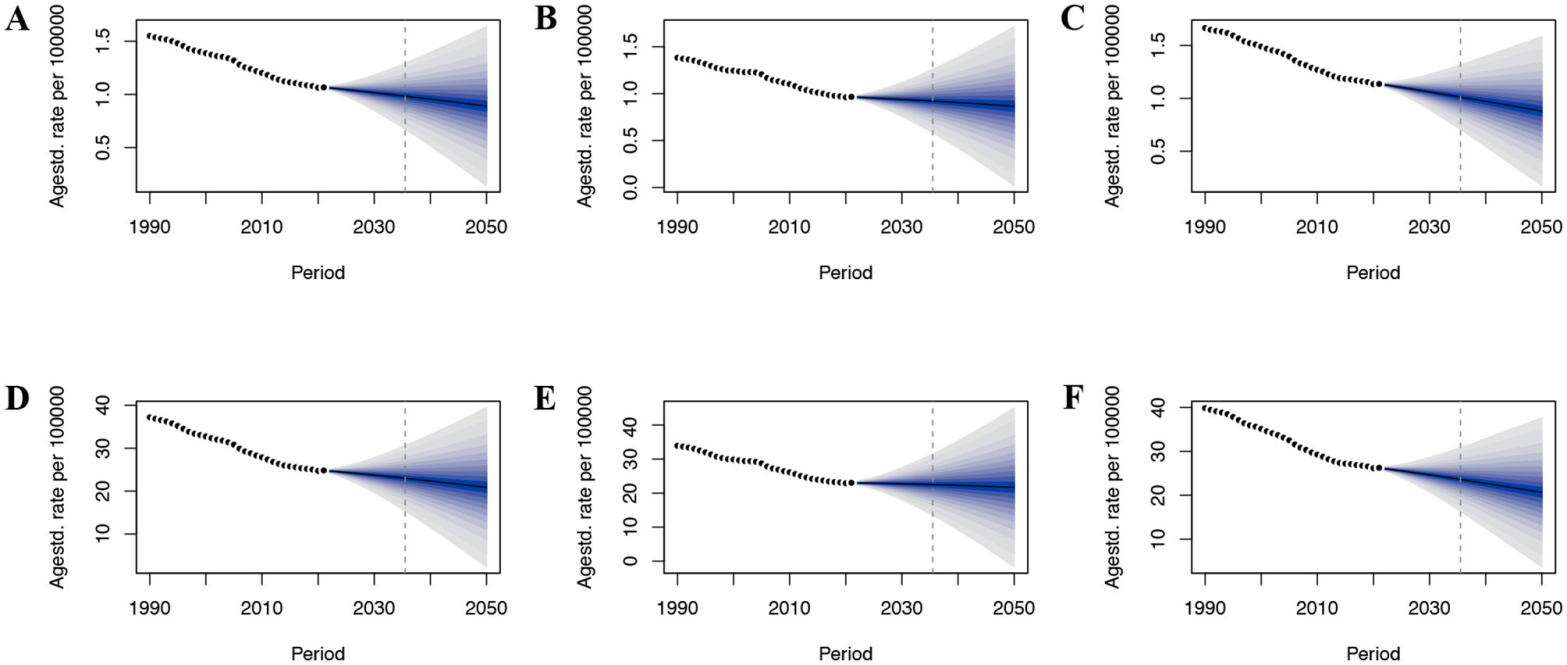
The predicted burden of colon and rectum cancer attributable to a diet low in calcium globally to 2050. **(A)** ASMR in both; **(B)** ASMR in male; **(C)** ASMR in female; **(D)** ASDR in both; **(E)** ASDR in male; **(F)** ASDR in female; ASMR, age-standardized mortality rate; ASDR, age-standardized DALY rate.

By employing the BAPC model, we estimated the worldwide impact of CRC linked to DLC over the period from 2022 through 2050. This analytical approach took age, period, and cohort into account. It made use of historical data that covered a time span of 32 years, starting from 1990 and ending in 2021. Over the coming decades, the CRC burden associated with DLC is expected to exhibit a declining trend, particularly among women (Figure 6). By 2050, CRC's ASMR and ASDR connected with DLC are projected to reach 0.89 and 20.88 per 100,000 population, respectively (Figures 6A, D). For males, these values are estimated at 0.87 and 21.70 per 100,000 population (Figures 6B, E), while for females, they are projected to be 0.88 and 20.66 per 100,000 population (Figures 6C, F).

## Discussion

Drawing on thorough data from the 2021 GBD report, this research renews the evaluation of how DLC affect health across varying geographical tiers, spanning the years 1990–2021. The study specifically investigates the time changes in rates of deaths and DALYs due to DLC for CRC and prostate cancer, while exploring their correlation with socioeconomic development levels. Although previous research has examined the worldwide impact of CRC caused by DLC from 1990 to 2019, the current study represents a significant advancement by incorporating the analysis of prostate cancer burden, which has been rarely investigated in the context of DLC (3). The findings not only reveal a series of meaningful insights but also corroborate and extend previous research in this field.

The findings reveal that while global deaths and DALYs from CRC linked to DLC rose by 55.3% and 40.7% between 1990 and 2021, the ASMR and ASDR saw significant declines. Their EAPCs were −1.33 and −1.45, respectively, with the Maldives exhibiting the most pronounced downward trend. China has continuously been in the leading position in CRC deaths and DALYs caused by DLC, which are closely associated with its large population base, accelerated aging process, and low calcium intake (27). The apparent contradiction of growing total deaths and DALYs amid falling ASRs likely stems from population expansion, longer lifespans, an aging society, and fewer early deaths (21, 28). Similar trends have been reported in other GBD studies (29, 30), highlighting the complexity of the relationship between colorectal cancer-related health issues caused by DLC and demographic changes. Surprisingly, for prostate cancer, deaths, DALYs, and ASRs associated with DLC were negative in some regions (e.g., China, India, and Zimbabwe), but did not reach statistical significance, implying that DLC might help prevent prostate cancer. The lack of statistical significance in prostate cancer analyses might be due to small sample sizes and high data heterogeneity across regions and SDI categories, which may obscure the impact of DLC on prostate cancer (31). Additionally, methodological limitations in GBD modeling, such as potential confounding from regional data quality and uncorrected correlates, may also lead to non-significant differences (32). These findings highlight the need to clarify the interplay of calcium, vitamin D, and parathyroid hormone through mechanistic studies and refined causal inference methods, thus elucidating calcium's role in prostate cancer epidemiology. These findings indicate that DLC impacts cancer risk differently depending on disease type. Future strategies should advocate for high-calcium foods in regions with high CRC incidence while ensuring adequate vitamin D intake, and encourage evidence-based moderate, low-calcium diets in areas with high prostate cancer prevalence, tailored to individual vitamin D status and parathyroid hormone levels, to effectively reduce the corresponding disease burden.

Socioeconomic determinants significantly drive the shifts in disease burden. Economic levels influence dietary patterns, and DLC has a notable impact on the burden of CRC (33, 34). The findings show that the ASMR and ASDR for colorectal cancer resulting from DLC are negatively correlated with the SDI, with a more marked decline in CRC burden seen in high-middle SDI regions. Likewise, characterized by higher HDI rankings saw a more pronounced decline in CRC burden associated with DLC over the period 1990–2021. However, the burden persists high in regions with low-middle SDI tiers, likely stemming from the dual challenges of inadequate calcium intake and limited capacity for cancer prevention and control in these areas (9). Notably, the prostate cancer burden linked to DLC is positively correlated with SDI, which could be due to the high calcium intake in high-income populations exacerbating the prostate cancer burden (9). This indicates a notable disparity in colorectal and prostate cancer burdens linked to DLC across income regions. The core reason for this difference is that calcium intake is commonly greater in nations with high-income status, whereas in nation with low-income status, it is much lower than the recommended amount (35). Research has indicated that the mean daily dietary calcium consumption in high-income nations may peak at 600–800 mg per day, whereas in specific African countries, it is even lower than 200 mg/day (36). In addition, the shortage of dairy products, insufficient micronutrient density of complementary foods, and lack of dietary diversity are also key factors contributing to calcium deficiency in low-income countries (3). The results emphasize the necessity to create customized dietary interventions and cancer-protective measures in areas with diverse economic progress levels. Specifically, improving dietary calcium intake can be achieved through three main approaches: behavioral interventions, which rely heavily on individual habits and the ability to change; personalized supplementation, which provides a direct means of addressing individual calcium deficiencies, particularly in populations known to have insufficient calcium intake; and food fortification, which, as a population-level strategy, aims to improve calcium intake on a large scale through the addition of calcium to food products, thereby provide a systemic solution to the dietary gap (35).

This study found that, from a gender perspective, The CRC burden linked to DLC is notably greater in women compared to men, as reflected by higher ASMR and ASDR. However, Over the timeframe spanning 1990 to 2021, it appears that the reduction in ASRs was occurring at a quicker pace in women than in men, while the reduction in CRC burden among men was slower, potentially due to women's greater tendency to engage in health-promoting behaviors (37). Thus, it is advised that gender-specific health education initiatives be implemented and that early CRC screening be strengthened to reduce the disease burden and narrow gender disparities. From an age perspective, the mortality and DALY rates for CRC related to DLC rose progressively with advancing age. Conversely, Prostate cancer-related mortality and DALY rates linked to DLC showed a decline with increasing age, especially in low SDI regions. This is due to the decreased intestinal calcium absorption capacity of the elderly due to the decline in digestive system function, coupled with the low intake of calcium-rich foods such as milk, resulting in insufficient calcium intake in the body, which in turn exacerbates age-related health risks (38, 39). To address this issue, it is recommended that education on calcium intake be promoted among high-risk populations, especially in specific age groups and among women, encouraging the consumption of appropriate amounts of dairy products or calcium supplements (40).

Despite meticulously examining the correlation between DLC and the burden of colorectal and prostate cancers based on the 2021 GBD data, a few limitations need to be recognized. First, The reliability of GBD data hinges on the comprehensiveness and correctness of cancer registries, vital statistics systems, and other data repositories in nations and regions. Potential recall bias and measurement errors, as well as missing or lower-quality data in some SDI regions, may result in biased disease burden estimates. This is particularly relevant for calcium intake estimates, which rely on GBD-standardized models with residual discrepancies due to regional variations in primary data collection methods. Second, while observational studies can identify potential links between DLC and cancer risk, they fall short of proving causation. This correlation may be affected by unaccounted variables, including genetic predisposition, lifestyle habits like smoking and alcohol use, exercise patterns, and external environmental influences. These confounders were not fully controlled for, potentially affecting result interpretation. Third, negative values for prostate cancer deaths, DALYs, and their ASRs due to DLC precluded the calculation of the EAPC and predictive analysis for prostate cancer. Additionally, although the BAPC model was utilized to anticipate upcoming trends, the predictions depend on assumptions and model parameters derived from existing data, and future changes in socioeconomic conditions, dietary patterns, and healthcare systems may affect the accuracy of these predictions. Finally, the GBD research solely evaluated the health impacts of DLC and failed to account for the implications of moderate or high calcium consumption. Moreover, it failed to account for critical interactions between calcium and cofactors such as vitamin D, phosphorus, magnesium, and parathyroid hormone. These omissions may potentially hinder a thorough grasp of the intricate connection between calcium intake and the burden of cancer. Therefore, future research should focus on enhancing data accuracy, accounting for potential confounding variables, and investigating the dose-response link between calcium consumption and cancer burden to provide more robust scientific evidence.

## Conclusions

Using 2021 GBD data, this study examined DLC on CRC and prostate cancer burden, revealing the substantial effect of calcium deficiency on the global cancer burden. The study found that DLC significantly increased the burden of CRC but the effect on prostate cancer was small and did not reach a statistical difference, suggesting only a reverse effect. The research innovatively revealed the disease-specific effects of DLC on cancer burden and highlighted socioeconomic influences on disease burden changes. These findings offer important evidence for formulating targeted nutritional intervention policies and cancer prevention strategies, particularly promoting high-calcium foods in CRC-high areas and encouraging moderate calcium intake in prostate cancer-high areas. Further exploration is needed to optimize global public health interventions by examining different calcium levels and cancer burden.

## Data Availability

The original contributions presented in the study are included in the article/Supplementary material, further inquiries can be directed to the corresponding authors.
