# Supplementary material for: The health burden of disease attributable to low calcium intake: a comprehensive analysis of trends and socioeconomic impacts from 1990 to 2021
Source: Front Nutr. 2025 Jul 9;12:1594656. doi: 10.3389/fnut.2025.1594656 (PMC12283273; doi:10.3389/fnut.2025.1594656)
Supplement: Supplementary file 1 [file Table_1.docx]

**Supplementary material**


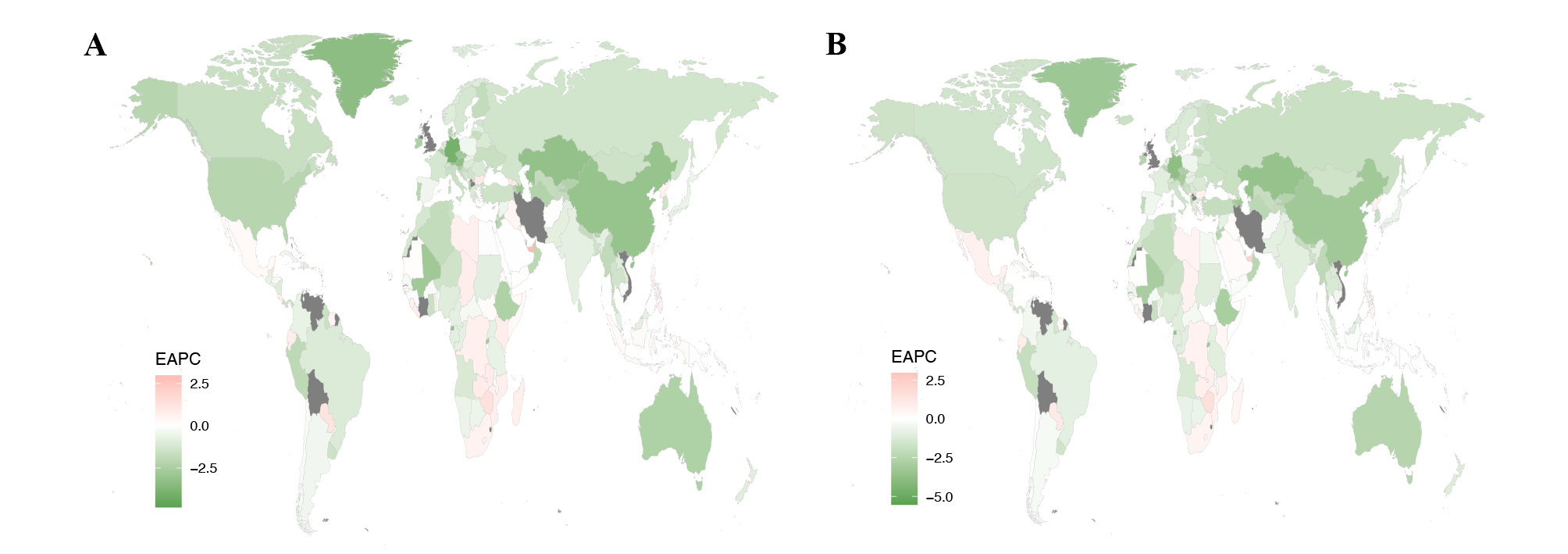


**Figure S1. The EAPC of ASMR and ASDR attributable to diet low in calcium for colon and rectum cancer among countries and territories in 2021.** A. ASMR; B. ASDR. EAPC, estimated annual percentage change; ASMR, age-standardized mortality rate; ASDR, age-standardized DALY rate; DALYs, disability-adjusted life year rates.


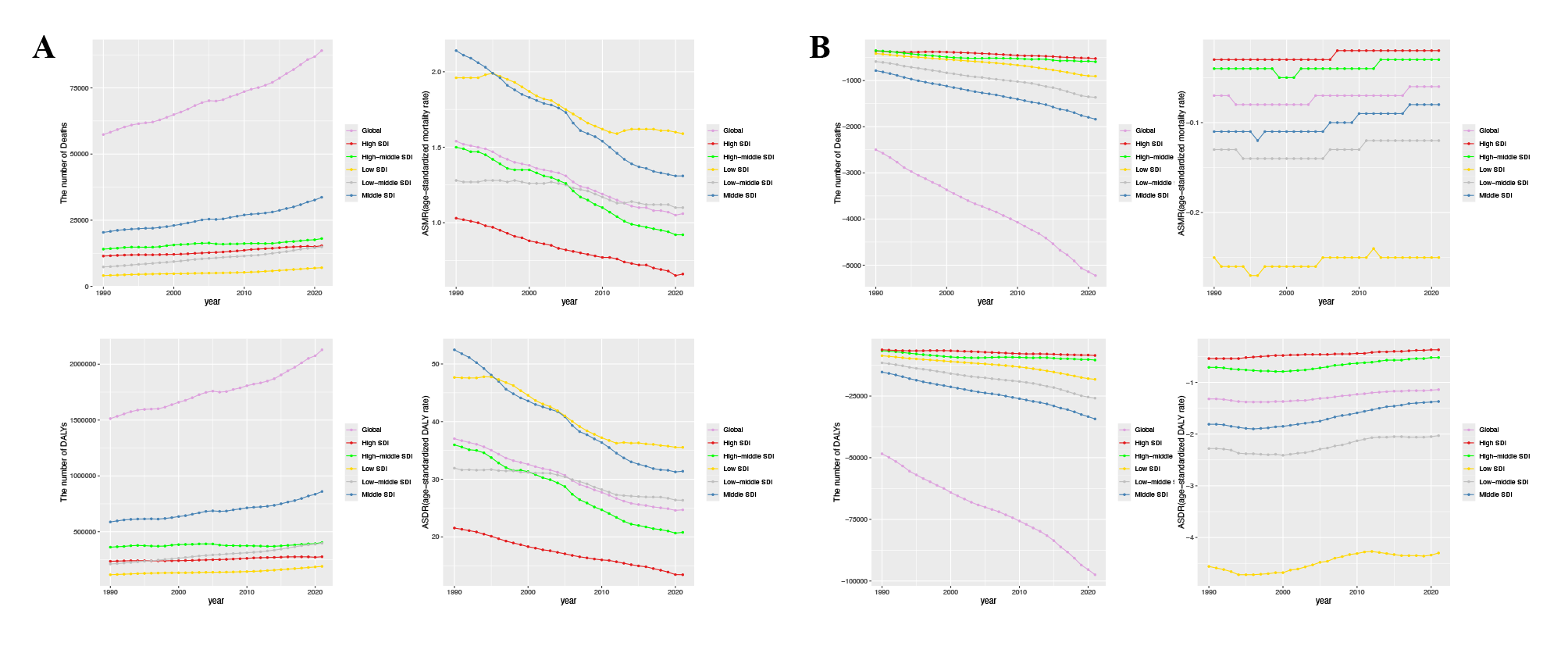


**Figure S2. The number and rate of deaths and DALYs attributable to diet low in calcium for prostate cancer and colon and rectum cancer among global and different SDI regions from 1990 to 2021.** A. Colon and rectum cancer; B. Prostate cancer. DALYs, disability-adjusted life year rates; SDI, sociodemographic index.


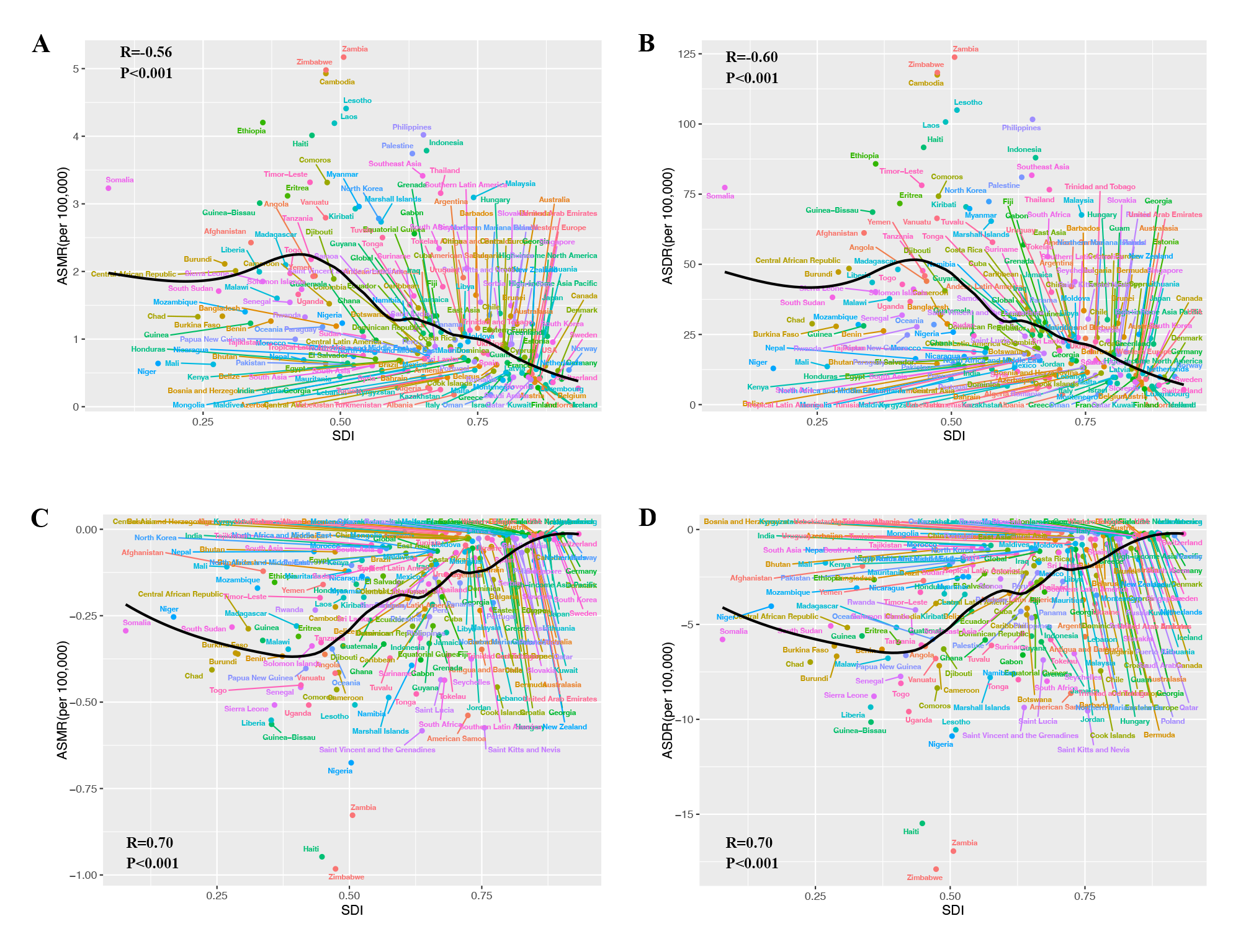


**Figure S3. The relationship between ASMR/ASDR attributable to diet low in calcium and SDI for prostate cancer and colon and rectum cancer among 204 countries in 2021**. A. ASMR of colon and rectum cancer; B. ASDR of colon and rectum cancer; C. ASMR of prostate cancer; D. ASDR of prostate cancer. ASMR, age-standardized mortality rate; ASDR, age-standardized DALY rate; DALYs, disability-adjusted life year rates; SDI, sociodemographic index.

| Table S1. The deaths cases, age-standardized deaths, and temporal trends of colon and rectum cancer attributable to diet low in calcium in 204 countries, 1990 and 2021 | | | | | |
| --- | --- | --- | --- | --- | --- |
|  | 1990 | 1990 | 2021 | 2021 | 1990-2021 |
| Location | Deaths cases  No. (95% UI) | ASMR per 100,000  No. (95% UI) | Deaths cases  No. (95% UI) | ASMR per 100,000  No. (95% UI) | EAPC  No. (95% CI) |
| Afghanistan | 144 (54,241) | 2.22 (0.93,3.68) | 231 (103,379) | 2.43 (1.21,3.91) | -0.09 (-0.35 , 0.17) |
| Albania | 6 (4,9) | 0.35 (0.23,0.51) | 8 (4,12) | 0.18 (0.1,0.28) | -2.21 (-2.55 , -1.87) |
| Algeria | 46 (31,60) | 0.51 (0.36,0.68) | 84 (54,122) | 0.29 (0.18,0.4) | -1.78 (-1.97 , -1.59) |
| American Samoa | 0 (0,0) | 1.73 (1.24,2.3) | 1 (1,1) | 1.91 (1.34,2.54) | 0.39 (0.27 , 0.51) |
| Andorra | 0 (0,0) | 0.41 (0.24,0.64) | 0 (0,1) | 0.27 (0.15,0.41) | -1.02 (-1.24 , -0.79) |
| Angola | 84 (58,117) | 2.45 (1.73,3.29) | 194 (128,272) | 1.93 (1.28,2.77) | -1.12 (-1.26 , -0.98) |
| Antigua and Barbuda | 1 (0,1) | 0.94 (0.68,1.22) | 1 (1,1) | 1.16 (0.84,1.52) | 0.7 (0.5 , 0.9) |
| Argentina | 443 (318,575) | 1.45 (1.04,1.87) | 644 (450,857) | 1.12 (0.79,1.49) | -0.4 (-0.63 , -0.17) |
| Armenia | 28 (20,35) | 1.05 (0.75,1.34) | 26 (17,35) | 0.6 (0.4,0.8) | -2.1 (-2.32 , -1.87) |
| Australia | 184 (126,247) | 0.96 (0.66,1.29) | 252 (165,352) | 0.51 (0.34,0.71) | -2.37 (-2.53 , -2.2) |
| Austria | 126 (87,169) | 1.01 (0.7,1.36) | 75 (50,105) | 0.36 (0.24,0.5) | -3.24 (-3.29 , -3.18) |
| Azerbaijan | 40 (28,53) | 0.83 (0.59,1.09) | 41 (27,59) | 0.43 (0.28,0.61) | -2.81 (-3.1 , -2.52) |
| Bahrain | 1 (1,1) | 0.82 (0.59,1.1) | 4 (2,5) | 0.6 (0.38,0.83) | -1.28 (-1.57 , -1) |
| Bangladesh | 818 (602,1110) | 1.76 (1.3,2.39) | 1564 (1046,2285) | 1.19 (0.81,1.7) | -1.42 (-1.58 , -1.26) |
| Barbados | 4 (3,5) | 1.4 (1.01,1.79) | 9 (6,12) | 1.65 (1.14,2.25) | 0.88 (0.57 , 1.18) |
| Belarus | 83 (57,112) | 0.65 (0.45,0.88) | 101 (65,148) | 0.62 (0.4,0.91) | -1.12 (-1.61 , -0.62) |
| Belgium | 157 (109,214) | 0.99 (0.69,1.35) | 113 (76,161) | 0.41 (0.28,0.58) | -2.56 (-2.73 , -2.38) |
| Belize | 0 (0,1) | 0.5 (0.36,0.65) | 2 (1,2) | 0.68 (0.49,0.88) | 1.07 (0.66 , 1.47) |
| Benin | 28 (21,35) | 1.49 (1.12,1.9) | 56 (37,76) | 1.27 (0.86,1.7) | -0.39 (-0.55 , -0.23) |
| Bermuda | 1 (1,1) | 1.68 (1.19,2.21) | 2 (1,3) | 1.22 (0.86,1.67) | -1.22 (-1.35 , -1.09) |
| Bhutan | 3 (2,4) | 1.19 (0.69,1.69) | 4 (2,5) | 0.64 (0.41,0.88) | -2.13 (-2.23 , -2.03) |
| Bolivarian Republic of Venezuela | 95 (70,118) | 1.05 (0.78,1.31) | 318 (208,456) | 1.11 (0.73,1.59) | -0.52 (-0.86 , -0.18) |
| Bosnia and Herzegovina | 38 (27,49) | 1.03 (0.74,1.33) | 47 (31,66) | 0.73 (0.48,1.04) | -1.27 (-1.45 , -1.09) |
| Botswana | 5 (3,7) | 1.1 (0.71,1.53) | 11 (7,15) | 0.87 (0.58,1.23) | -0.52 (-0.74 , -0.3) |
| Brazil | 732 (546,916) | 0.92 (0.68,1.15) | 1745 (1237,2279) | 0.71 (0.5,0.92) | -1.03 (-1.15 , -0.91) |
| Brunei Darussalam | 2 (1,3) | 2.06 (1.43,2.81) | 4 (3,5) | 1.34 (0.91,1.82) | -0.8 (-1.16 , -0.44) |
| Bulgaria | 111 (77,151) | 0.96 (0.67,1.29) | 187 (127,258) | 1.27 (0.86,1.75) | 0.94 (0.5 , 1.39) |
| Burkina Faso | 55 (39,73) | 1.47 (1.06,1.95) | 106 (70,144) | 1.34 (0.89,1.8) | -0.18 (-0.26 , -0.1) |
| Burundi | 38 (26,50) | 1.78 (1.22,2.33) | 88 (57,131) | 2.11 (1.39,3.09) | 0.2 (0.04 , 0.37) |
| Cambodia | 219 (136,305) | 5.05 (3.24,6.88) | 562 (390,770) | 4.93 (3.48,6.67) | -0.22 (-0.29 , -0.15) |
| Cameroon | 70 (51,89) | 1.81 (1.33,2.32) | 158 (101,227) | 1.52 (1.01,2.11) | -0.78 (-0.86 , -0.69) |
| Canada | 357 (254,476) | 1.1 (0.78,1.48) | 471 (324,642) | 0.6 (0.41,0.82) | -1.63 (-1.78 , -1.49) |
| Central African Republic | 22 (14,33) | 2.2 (1.49,3.19) | 39 (24,58) | 2.01 (1.28,2.93) | -0.33 (-0.49 , -0.17) |
| Chad | 29 (21,40) | 1.12 (0.81,1.54) | 66 (45,92) | 1.33 (0.92,1.81) | 0.78 (0.7 , 0.87) |
| Chile | 135 (100,168) | 1.47 (1.09,1.83) | 296 (219,382) | 1.14 (0.84,1.46) | -0.23 (-0.43 , -0.03) |
| China | 18903 (13486,24551) | 2.55 (1.81,3.28) | 20719 (14553,28272) | 1.04 (0.73,1.41) | -3.06 (-3.17 , -2.96) |
| Colombia | 181 (134,225) | 1.13 (0.84,1.41) | 473 (332,630) | 0.86 (0.6,1.14) | -0.69 (-0.98 , -0.39) |
| Commonwealth of the Bahamas | 2 (1,2) | 1.12 (0.82,1.43) | 5 (3,6) | 1.23 (0.84,1.67) | 0.41 (0.28 , 0.53) |
| Comoros | 5 (3,7) | 2.95 (1.94,3.97) | 15 (9,21) | 3.32 (2.16,4.67) | 0.38 (0.31 , 0.46) |
| Congo | 35 (23,48) | 3.67 (2.53,4.97) | 73 (51,99) | 3.07 (2.17,4.08) | -0.76 (-0.9 , -0.62) |
| Cook Islands | 0 (0,0) | 1.15 (0.84,1.52) | 0 (0,0) | 0.53 (0.36,0.73) | -2.56 (-2.77 , -2.35) |
| Costa Rica | 13 (9,17) | 0.76 (0.55,0.98) | 49 (34,66) | 0.89 (0.61,1.2) | 0.71 (0.53 , 0.89) |
| Croatia | 78 (55,103) | 1.42 (1,1.88) | 92 (64,128) | 0.93 (0.65,1.32) | -1.65 (-1.86 , -1.44) |
| Cuba | 127 (93,163) | 1.27 (0.93,1.63) | 252 (170,332) | 1.24 (0.84,1.63) | -0.4 (-0.56 , -0.23) |
| Cyprus | 7 (5,10) | 1.18 (0.76,1.64) | 13 (9,18) | 0.73 (0.5,0.96) | -1.06 (-1.28 , -0.85) |
| Czech Republic | 201 (139,268) | 1.44 (1,1.93) | 158 (105,218) | 0.69 (0.46,0.95) | -2.75 (-2.98 , -2.53) |
| Democratic People's Republic of Korea | 373 (254,535) | 2.53 (1.73,3.59) | 899 (563,1378) | 2.78 (1.75,4.25) | 0.54 (0.36 , 0.72) |
| Democratic Republic of the Congo | 260 (182,355) | 1.94 (1.33,2.64) | 759 (476,1141) | 2.36 (1.49,3.64) | 0.65 (0.6 , 0.7) |
| Denmark | 79 (55,106) | 0.92 (0.63,1.23) | 71 (48,100) | 0.53 (0.36,0.75) | -2.14 (-2.37 , -1.91) |
| Djibouti | 2 (2,3) | 2.11 (1.39,3) | 10 (6,15) | 1.89 (1.2,2.74) | -0.62 (-0.72 , -0.52) |
| Dominica | 0 (0,1) | 0.83 (0.58,1.13) | 1 (0,1) | 0.7 (0.47,0.97) | -0.42 (-0.54 , -0.29) |
| Dominican Republic | 42 (30,55) | 1.32 (0.95,1.7) | 97 (64,136) | 0.99 (0.65,1.39) | -0.8 (-1.14 , -0.47) |
| Ecuador | 40 (29,51) | 0.84 (0.62,1.06) | 146 (98,197) | 0.93 (0.63,1.24) | 0.77 (0.36 , 1.17) |
| Egypt | 219 (164,276) | 0.89 (0.66,1.13) | 371 (253,510) | 0.68 (0.48,0.93) | -0.08 (-0.49 , 0.33) |
| El Salvador | 24 (18,30) | 0.83 (0.62,1.03) | 45 (31,62) | 0.7 (0.49,0.97) | -0.42 (-0.62 , -0.21) |
| Equatorial Guinea | 5 (3,7) | 2.77 (1.87,3.76) | 6 (4,9) | 1.34 (0.84,2.01) | -3.17 (-3.72 , -2.62) |
| Eritrea | 31 (22,41) | 3.02 (2.18,4.04) | 74 (49,103) | 3.12 (2.12,4.26) | 0.21 (0.15 , 0.26) |
| Estonia | 13 (9,17) | 0.61 (0.43,0.83) | 14 (9,20) | 0.46 (0.3,0.65) | -1.34 (-1.54 , -1.14) |
| Ethiopia | 1300 (729,1757) | 7.58 (4.42,10.09) | 1555 (1125,1974) | 4.2 (3.05,5.32) | -2.37 (-2.57 , -2.18) |
| Federated States of Micronesia | 2 (1,2) | 3.37 (2.4,4.48) | 2 (1,3) | 2.96 (2.01,4.1) | -0.41 (-0.46 , -0.36) |
| Fiji | 5 (3,6) | 1.48 (1.04,1.93) | 9 (5,12) | 1.34 (0.87,1.86) | 0.01 (-0.24 , 0.26) |
| Finland | 34 (24,46) | 0.47 (0.33,0.64) | 41 (28,60) | 0.28 (0.19,0.4) | -1.76 (-1.92 , -1.6) |
| France | 645 (441,896) | 0.73 (0.5,1.01) | 806 (529,1150) | 0.46 (0.3,0.65) | -1.22 (-1.32 , -1.12) |
| Gabon | 17 (10,26) | 3.29 (1.9,4.81) | 23 (15,32) | 2.56 (1.73,3.53) | -0.82 (-0.91 , -0.73) |
| Georgia | 38 (26,50) | 0.62 (0.43,0.81) | 41 (28,55) | 0.68 (0.46,0.91) | 1 (0.62 , 1.38) |
| Germany | 1598 (1097,2155) | 1.18 (0.81,1.6) | 922 (590,1287) | 0.41 (0.27,0.57) | -4.01 (-4.25 , -3.77) |
| Ghana | 83 (59,108) | 1.56 (1.13,2.04) | 133 (89,172) | 0.98 (0.68,1.3) | -1.59 (-1.73 , -1.44) |
| Greece | 82 (57,111) | 0.54 (0.38,0.74) | 126 (84,173) | 0.42 (0.28,0.58) | -1.1 (-1.37 , -0.83) |
| Greenland | 0 (0,1) | 1.77 (1.19,2.45) | 0 (0,1) | 0.7 (0.45,1.01) | -3.37 (-3.54 , -3.2) |
| Grenada | 1 (1,1) | 1.19 (0.85,1.53) | 1 (1,2) | 1.34 (0.95,1.76) | 0.81 (0.64 , 0.98) |
| Guam | 1 (1,1) | 1.2 (0.86,1.56) | 1 (1,2) | 0.65 (0.44,0.88) | -1.36 (-1.75 , -0.97) |
| Guatemala | 34 (26,42) | 1.18 (0.89,1.45) | 116 (82,150) | 1.12 (0.79,1.45) | -0.67 (-1.02 , -0.33) |
| Guinea | 39 (29,51) | 1.27 (0.95,1.66) | 64 (44,90) | 1.23 (0.86,1.71) | -0.09 (-0.21 , 0.02) |
| Guinea-Bissau | 10 (7,14) | 2.82 (1.84,3.73) | 19 (13,25) | 3.01 (2.11,4) | 0.41 (0.34 , 0.48) |
| Guyana | 7 (5,9) | 1.98 (1.47,2.53) | 6 (4,9) | 1.09 (0.74,1.51) | -1.56 (-1.86 , -1.27) |
| Haiti | 134 (82,191) | 4.71 (3.05,6.65) | 254 (161,386) | 4.01 (2.59,5.94) | -0.53 (-0.59 , -0.46) |
| Honduras | 14 (10,19) | 0.73 (0.52,0.95) | 37 (25,53) | 0.64 (0.43,0.92) | -0.3 (-0.48 , -0.12) |
| Hungary | 249 (170,340) | 1.73 (1.18,2.35) | 284 (195,387) | 1.38 (0.95,1.89) | -1.04 (-1.42 , -0.65) |
| Iceland | 1 (1,2) | 0.43 (0.29,0.6) | 2 (1,2) | 0.28 (0.18,0.39) | -1.57 (-1.76 , -1.38) |
| India | 2901 (2163,3764) | 0.68 (0.5,0.88) | 5976 (4385,7587) | 0.54 (0.4,0.68) | -0.77 (-0.88 , -0.65) |
| Indonesia | 3273 (2373,4165) | 3.5 (2.56,4.45) | 8078 (5607,10759) | 3.79 (2.67,4.99) | 0.2 (0.06 , 0.35) |
| Iraq | 69 (50,89) | 0.87 (0.63,1.13) | 275 (186,373) | 1.28 (0.87,1.71) | 0.43 (0.14 , 0.73) |
| Ireland | 30 (20,41) | 0.74 (0.51,1.01) | 27 (17,39) | 0.32 (0.21,0.47) | -2.4 (-2.53 , -2.26) |
| Islamic Republic of Iran | 233 (171,300) | 1.07 (0.79,1.36) | 533 (382,685) | 0.74 (0.53,0.96) | -0.54 (-0.87 , -0.21) |
| Israel | 41 (28,55) | 0.87 (0.59,1.16) | 55 (36,79) | 0.41 (0.27,0.58) | -2.84 (-3.06 , -2.62) |
| Italy | 729 (511,977) | 0.81 (0.57,1.08) | 842 (551,1163) | 0.48 (0.32,0.67) | -1.57 (-1.63 , -1.51) |
| Jamaica | 17 (12,22) | 0.93 (0.68,1.19) | 35 (23,49) | 1.1 (0.73,1.55) | 0.76 (0.45 , 1.07) |
| Japan | 2257 (1646,2882) | 1.38 (1.01,1.76) | 5024 (3508,6630) | 1.1 (0.78,1.44) | -0.57 (-0.67 , -0.47) |
| Jordan | 15 (11,20) | 1.26 (0.9,1.64) | 47 (29,71) | 0.72 (0.45,1.08) | -2.15 (-2.3 , -1.99) |
| Kazakhstan | 89 (62,120) | 0.74 (0.51,0.99) | 50 (33,69) | 0.3 (0.2,0.42) | -3.19 (-3.58 , -2.79) |
| Kenya | 42 (30,56) | 0.55 (0.39,0.74) | 122 (83,171) | 0.61 (0.42,0.85) | 0.74 (0.56 , 0.92) |
| Kingdom of Eswatini | 6 (4,9) | 2.59 (1.68,3.56) | 14 (9,21) | 2.87 (1.82,4.08) | 0.7 (0.28 , 1.12) |
| Kiribati | 1 (1,1) | 3.23 (2.4,4.28) | 2 (1,3) | 2.93 (2.13,3.95) | -0.48 (-0.55 , -0.42) |
| Kuwait | 2 (1,2) | 0.32 (0.22,0.44) | 8 (5,11) | 0.3 (0.19,0.41) | 0.22 (-0.08 , 0.51) |
| Kyrgyzstan | 15 (11,20) | 0.53 (0.38,0.71) | 12 (8,17) | 0.28 (0.18,0.39) | -2.27 (-2.46 , -2.09) |
| Lao People's Democratic Republic | 105 (55,151) | 5.26 (2.86,7.45) | 180 (119,248) | 4.19 (2.85,5.67) | -0.94 (-1.03 , -0.86) |
| Latvia | 21 (15,29) | 0.59 (0.41,0.8) | 22 (14,31) | 0.5 (0.32,0.7) | -0.87 (-1.33 , -0.41) |
| Lebanon | 15 (9,21) | 0.77 (0.49,1.08) | 35 (23,50) | 0.54 (0.35,0.78) | -0.65 (-0.87 , -0.43) |
| Lesotho | 19 (13,25) | 2.41 (1.65,3.26) | 43 (27,63) | 4.41 (2.82,6.37) | 2.74 (2.29 , 3.18) |
| Liberia | 17 (12,22) | 1.63 (1.2,2.16) | 36 (22,58) | 2 (1.24,3.14) | 0.77 (0.66 , 0.89) |
| Libya | 17 (11,23) | 0.94 (0.63,1.3) | 52 (33,73) | 1.08 (0.69,1.52) | 0.68 (0.53 , 0.83) |
| Lithuania | 37 (26,49) | 0.82 (0.58,1.08) | 36 (24,50) | 0.55 (0.37,0.77) | -1.67 (-1.87 , -1.47) |
| Luxembourg | 4 (3,6) | 0.83 (0.56,1.11) | 4 (3,6) | 0.37 (0.24,0.51) | -2.58 (-2.78 , -2.38) |
| Madagascar | 78 (55,103) | 1.69 (1.22,2.22) | 202 (135,280) | 2.1 (1.42,2.87) | 0.66 (0.6 , 0.73) |
| Malawi | 56 (41,71) | 1.59 (1.19,2.04) | 108 (76,153) | 1.6 (1.15,2.18) | -0.21 (-0.39 , -0.03) |
| Malaysia | 343 (263,438) | 3.94 (2.99,5.01) | 794 (603,1006) | 3.09 (2.36,3.91) | -0.76 (-0.91 , -0.61) |
| Maldives | 1 (1,2) | 1.66 (1.08,2.23) | 1 (1,2) | 0.44 (0.3,0.6) | -4.82 (-5.1 , -4.53) |
| Mali | 47 (35,60) | 1.38 (1.04,1.74) | 47 (30,64) | 0.63 (0.41,0.85) | -2.78 (-3.18 , -2.39) |
| Malta | 4 (3,5) | 0.91 (0.64,1.23) | 5 (4,8) | 0.5 (0.33,0.7) | -1.76 (-1.99 , -1.54) |
| Marshall Islands | 0 (0,1) | 3.03 (2.14,4) | 1 (1,1) | 2.73 (1.91,3.77) | -0.31 (-0.38 , -0.24) |
| Mauritania | 4 (3,5) | 0.43 (0.29,0.58) | 8 (5,12) | 0.45 (0.27,0.63) | 0.1 (-0.01 , 0.22) |
| Mauritius | 6 (4,7) | 0.86 (0.64,1.07) | 16 (11,21) | 0.91 (0.66,1.17) | -0.22 (-0.49 , 0.05) |
| Mexico | 232 (173,289) | 0.62 (0.46,0.77) | 807 (595,1040) | 0.66 (0.48,0.84) | 0.27 (0.03 , 0.51) |
| Mongolia | 8 (5,10) | 0.73 (0.48,1.01) | 11 (7,16) | 0.54 (0.35,0.76) | -1.47 (-1.64 , -1.3) |
| Montenegro | 2 (1,3) | 0.32 (0.21,0.44) | 4 (2,5) | 0.39 (0.25,0.57) | 0.47 (0.24 , 0.7) |
| Morocco | 154 (109,207) | 1.14 (0.8,1.52) | 258 (165,368) | 0.81 (0.51,1.14) | -1.22 (-1.33 , -1.1) |
| Mozambique | 60 (45,77) | 1.27 (0.95,1.59) | 120 (80,159) | 1.4 (0.95,1.86) | 0.69 (0.57 , 0.81) |
| Myanmar | 1005 (609,1481) | 4.5 (2.79,6.53) | 1338 (906,1813) | 2.96 (2.02,4.01) | -1.84 (-2.04 , -1.64) |
| Namibia | 9 (6,11) | 1.46 (1.09,1.9) | 16 (11,22) | 1.3 (0.92,1.76) | -0.57 (-0.74 , -0.4) |
| Nepal | 101 (65,146) | 1.13 (0.76,1.63) | 149 (101,205) | 0.69 (0.47,0.95) | -1.72 (-2.04 , -1.4) |
| Netherlands | 132 (88,179) | 0.64 (0.43,0.88) | 175 (116,243) | 0.46 (0.31,0.64) | -0.92 (-1.14 , -0.71) |
| New Zealand | 63 (45,84) | 1.64 (1.17,2.19) | 109 (75,146) | 1.22 (0.85,1.64) | -0.89 (-1.1 , -0.68) |
| Nicaragua | 14 (11,17) | 0.98 (0.73,1.21) | 34 (24,46) | 0.75 (0.52,1) | -0.93 (-1.08 , -0.78) |
| Niger | 22 (15,31) | 0.95 (0.68,1.32) | 41 (27,58) | 0.64 (0.43,0.9) | -1.44 (-1.77 , -1.12) |
| Nigeria | 642 (456,860) | 1.62 (1.17,2.14) | 953 (669,1272) | 1.24 (0.88,1.62) | -0.91 (-0.96 , -0.86) |
| Northern Mariana Islands | 0 (0,0) | 1.22 (0.83,1.66) | 1 (0,1) | 1.35 (0.95,1.73) | 0.54 (0.21 , 0.88) |
| Norway | 55 (38,75) | 0.75 (0.51,1.02) | 62 (42,87) | 0.54 (0.37,0.75) | -0.96 (-1.05 , -0.88) |
| Oman | 2 (1,3) | 0.35 (0.23,0.5) | 3 (2,4) | 0.18 (0.11,0.25) | -1.99 (-2.24 , -1.74) |
| Pakistan | 410 (303,545) | 0.78 (0.58,1.05) | 684 (475,941) | 0.63 (0.44,0.85) | -0.8 (-0.85 , -0.74) |
| Palestine | 36 (24,49) | 4.52 (3.08,6.1) | 81 (58,104) | 3.74 (2.67,4.78) | -0.4 (-0.65 , -0.15) |
| Panama | 22 (16,26) | 1.51 (1.14,1.85) | 44 (30,60) | 0.99 (0.68,1.33) | -1.53 (-1.64 , -1.42) |
| Papua New Guinea | 16 (11,23) | 1 (0.7,1.37) | 47 (33,62) | 1 (0.7,1.33) | 0.01 (-0.08 , 0.1) |
| Paraguay | 14 (10,18) | 0.65 (0.45,0.84) | 46 (30,65) | 0.83 (0.55,1.16) | 1.2 (1.05 , 1.35) |
| Peru | 141 (103,184) | 1.26 (0.92,1.65) | 268 (177,377) | 0.81 (0.53,1.14) | -1.96 (-2.24 , -1.67) |
| Philippines | 1017 (772,1285) | 3.66 (2.79,4.68) | 3176 (2405,4011) | 4.02 (3.06,5.07) | 0.53 (0.46 , 0.6) |
| Plurinational State of Bolivia | 119 (73,170) | 4.12 (2.56,5.88) | 283 (181,411) | 3.45 (2.22,4.95) | -0.65 (-0.68 , -0.62) |
| Poland | 401 (281,523) | 0.94 (0.66,1.23) | 704 (486,952) | 0.93 (0.64,1.25) | -0.43 (-0.83 , -0.02) |
| Portugal | 184 (130,241) | 1.41 (1,1.85) | 193 (131,264) | 0.67 (0.45,0.91) | -2.1 (-2.2 , -1.99) |
| Principality of Monaco | 0 (0,1) | 0.65 (0.39,0.93) | 1 (1,1) | 0.74 (0.48,1.06) | 0.44 (0.39 , 0.48) |
| Puerto Rico | 36 (26,47) | 1.05 (0.75,1.36) | 63 (43,85) | 0.81 (0.56,1.1) | -0.91 (-1.07 , -0.76) |
| Qatar | 1 (0,1) | 0.72 (0.49,1.01) | 2 (1,3) | 0.4 (0.24,0.59) | -2.17 (-2.7 , -1.63) |
| Republic of Cabo Verde | 1 (1,2) | 0.53 (0.38,0.69) | 3 (2,4) | 0.75 (0.5,1.01) | 0.48 (0.1 , 0.87) |
| Republic of Côte d'Ivoire | 38 (27,49) | 1.14 (0.83,1.45) | 90 (62,130) | 0.95 (0.67,1.32) | -0.72 (-0.85 , -0.58) |
| Republic of Korea | 349 (250,439) | 1.43 (1.03,1.82) | 876 (577,1221) | 0.95 (0.63,1.33) | -1.47 (-1.66 , -1.28) |
| Republic of Moldova | 46 (33,59) | 1.1 (0.79,1.42) | 58 (40,81) | 0.96 (0.67,1.34) | -0.27 (-0.58 , 0.04) |
| Republic of Nauru | 0 (0,0) | 2.32 (1.4,3.43) | 0 (0,0) | 2.58 (1.55,3.56) | 0.2 (-0.4 , 0.79) |
| Republic of Niue | 0 (0,0) | 2.14 (1.54,2.8) | 0 (0,0) | 1.71 (1.15,2.26) | -0.96 (-1.05 , -0.87) |
| Republic of Palau | 0 (0,0) | 2.59 (1.78,3.62) | 0 (0,0) | 1.97 (1.33,2.67) | -0.65 (-0.78 , -0.51) |
| Republic of San Marino | 0 (0,0) | 0.63 (0.39,0.92) | 0 (0,0) | 0.27 (0.16,0.43) | -2 (-2.32 , -1.67) |
| Republic of the Gambia | 3 (2,4) | 0.87 (0.62,1.12) | 7 (5,10) | 0.8 (0.53,1.08) | -0.5 (-0.67 , -0.34) |
| Romania | 142 (96,195) | 0.53 (0.36,0.74) | 180 (118,257) | 0.46 (0.3,0.66) | -1.11 (-1.43 , -0.8) |
| Russian Federation | 1968 (1442,2532) | 1.12 (0.82,1.44) | 2223 (1525,2913) | 0.92 (0.63,1.2) | -1.35 (-1.65 , -1.05) |
| Rwanda | 54 (37,74) | 2.13 (1.49,2.92) | 68 (41,97) | 1.33 (0.81,1.88) | -2.75 (-3.16 , -2.35) |
| Saint Kitts and Nevis | 1 (1,1) | 2 (1.46,2.57) | 1 (1,1) | 1.55 (1.09,2.02) | -0.21 (-0.4 , -0.01) |
| Saint Lucia | 1 (1,1) | 1.24 (0.91,1.56) | 2 (1,3) | 0.89 (0.63,1.19) | -1.36 (-1.64 , -1.08) |
| Saint Vincent and the Grenadines | 2 (1,2) | 2.2 (1.66,2.71) | 2 (1,2) | 1.15 (0.83,1.49) | -2.42 (-2.8 , -2.04) |
| Samoa | 1 (1,2) | 1.89 (1.35,2.45) | 2 (1,2) | 1.34 (0.91,1.77) | -1.55 (-1.79 , -1.3) |
| Sao Tome and Principe | 2 (1,2) | 2.52 (1.85,3.17) | 2 (2,3) | 2.51 (1.81,3.34) | 0.16 (0.09 , 0.23) |
| Saudi Arabia | 25 (17,35) | 0.46 (0.31,0.64) | 80 (50,113) | 0.44 (0.29,0.6) | -0.03 (-0.4 , 0.34) |
| Senegal | 47 (34,60) | 1.63 (1.21,2.08) | 103 (70,141) | 1.54 (1.06,2.11) | -0.27 (-0.4 , -0.14) |
| Serbia | 131 (87,186) | 1.4 (0.94,1.99) | 182 (119,259) | 1.06 (0.69,1.51) | -1.37 (-1.59 , -1.15) |
| Seychelles | 1 (1,2) | 2.42 (1.81,3.04) | 2 (2,3) | 2.05 (1.49,2.65) | -0.23 (-0.42 , -0.05) |
| Sierra Leone | 32 (23,43) | 1.71 (1.24,2.23) | 62 (41,84) | 1.85 (1.24,2.48) | 0.53 (0.41 , 0.65) |
| Singapore | 35 (26,45) | 1.76 (1.28,2.26) | 63 (46,84) | 0.76 (0.54,1) | -2.85 (-3.04 , -2.66) |
| Slovakia | 106 (75,140) | 1.78 (1.26,2.34) | 144 (94,202) | 1.5 (0.98,2.11) | -0.64 (-0.82 , -0.46) |
| Slovenia | 22 (15,30) | 0.89 (0.61,1.22) | 22 (15,32) | 0.44 (0.29,0.64) | -2.59 (-2.78 , -2.4) |
| Socialist Republic of Viet Nam | 1248 (907,1590) | 3.18 (2.32,4.06) | 3184 (2206,4098) | 3.37 (2.37,4.31) | 0.04 (-0.09 , 0.17) |
| Solomon Islands | 2 (1,3) | 1.96 (1.24,2.75) | 5 (4,8) | 1.74 (1.17,2.4) | -0.35 (-0.45 , -0.25) |
| Somalia | 66 (40,99) | 2.99 (1.86,4.53) | 174 (104,264) | 3.23 (1.98,4.82) | 0.28 (0.21 , 0.36) |
| South Africa | 371 (271,508) | 1.93 (1.4,2.67) | 989 (749,1208) | 2.34 (1.76,2.85) | 0.51 (0.22 , 0.79) |
| South Sudan | 36 (23,51) | 1.56 (1.01,2.22) | 56 (36,80) | 1.71 (1.12,2.38) | 0.18 (0.08 , 0.28) |
| Spain | 550 (390,721) | 1.01 (0.72,1.33) | 895 (607,1226) | 0.78 (0.52,1.06) | -0.45 (-0.64 , -0.26) |
| Sri Lanka | 133 (103,163) | 1.38 (1.07,1.7) | 216 (134,310) | 0.84 (0.53,1.19) | -1.31 (-1.5 , -1.12) |
| Sudan | 114 (73,164) | 1.3 (0.87,1.86) | 190 (119,296) | 1.04 (0.65,1.58) | -0.85 (-0.91 , -0.8) |
| Suriname | 3 (3,4) | 1.48 (1.1,1.88) | 10 (7,14) | 1.64 (1.09,2.26) | 0.44 (0.04 , 0.85) |
| Sweden | 86 (59,119) | 0.53 (0.36,0.73) | 90 (58,125) | 0.36 (0.23,0.5) | -1.24 (-1.37 , -1.1) |
| Switzerland | 46 (31,63) | 0.41 (0.28,0.57) | 57 (38,80) | 0.27 (0.18,0.38) | -1.36 (-1.53 , -1.19) |
| Syrian Arab Republic | 28 (19,38) | 0.57 (0.39,0.76) | 60 (38,86) | 0.53 (0.35,0.75) | -0.5 (-0.79 , -0.21) |
| Taiwan (Province of China) | 160 (117,208) | 1.12 (0.81,1.44) | 556 (386,726) | 1.3 (0.9,1.69) | 0.43 (0.1 , 0.75) |
| Tajikistan | 30 (21,39) | 1.12 (0.8,1.45) | 41 (27,57) | 0.76 (0.51,1.03) | -1.79 (-2.21 , -1.37) |
| Thailand | 1444 (1063,1837) | 4.44 (3.27,5.65) | 3388 (2330,4563) | 3.16 (2.18,4.24) | -1.36 (-1.5 , -1.21) |
| The former Yugoslav Republic of Macedonia | 18 (13,25) | 1.04 (0.73,1.41) | 27 (17,39) | 0.94 (0.62,1.33) | -0.73 (-1.15 , -0.31) |
| Timor-Leste | 8 (6,11) | 3.24 (2.23,4.31) | 27 (18,36) | 3.32 (2.27,4.38) | 0.11 (-0.05 , 0.28) |
| Togo | 18 (13,24) | 1.7 (1.22,2.22) | 62 (40,89) | 1.97 (1.27,2.77) | 0.56 (0.51 , 0.62) |
| Tokelau | 0 (0,0) | 2.76 (1.92,3.64) | 0 (0,0) | 1.77 (1.21,2.42) | -1.49 (-1.54 , -1.45) |
| Tonga | 1 (1,1) | 1.73 (1.25,2.22) | 1 (1,2) | 1.51 (1.05,2.01) | -0.39 (-0.49 , -0.3) |
| Trinidad and Tobago | 10 (7,13) | 1.32 (0.96,1.67) | 18 (12,26) | 0.97 (0.66,1.39) | -1.41 (-1.6 , -1.23) |
| Tunisia | 29 (20,39) | 0.67 (0.46,0.89) | 54 (34,80) | 0.44 (0.27,0.65) | -1.61 (-1.71 , -1.51) |
| Turkey | 264 (178,363) | 0.84 (0.56,1.14) | 449 (296,628) | 0.52 (0.34,0.72) | -1.47 (-1.64 , -1.3) |
| Turkmenistan | 8 (6,11) | 0.45 (0.33,0.58) | 10 (6,14) | 0.25 (0.16,0.37) | -2.25 (-2.6 , -1.88) |
| Tuvalu | 0 (0,0) | 3.25 (2.31,4.42) | 0 (0,0) | 2.5 (1.73,3.45) | -0.75 (-0.81 , -0.69) |
| Uganda | 99 (70,132) | 1.77 (1.26,2.32) | 213 (143,290) | 1.66 (1.15,2.24) | -0.74 (-0.97 , -0.5) |
| Ukraine | 761 (534,998) | 1.07 (0.75,1.4) | 632 (399,922) | 0.79 (0.5,1.16) | -1.62 (-1.95 , -1.28) |
| United Arab Emirates | 2 (1,4) | 0.63 (0.38,0.94) | 15 (9,23) | 0.73 (0.47,1.09) | 2.98 (2.2 , 3.75) |
| United Kingdom of Great Britain and Northern Ireland | 965 (677,1281) | 1.02 (0.72,1.36) | 718 (492,970) | 0.49 (0.34,0.66) | -2.26 (-2.57 , -1.96) |
| United Republic of Tanzania | 248 (181,328) | 2.53 (1.86,3.3) | 490 (325,662) | 2.18 (1.47,2.91) | -0.69 (-0.77 , -0.6) |
| United States of America | 2682 (1848,3611) | 0.81 (0.56,1.09) | 2501 (1704,3407) | 0.41 (0.28,0.56) | -2.11 (-2.21 , -2) |
| United States Virgin Islands | 1 (1,2) | 1.69 (1.16,2.22) | 1 (1,2) | 0.77 (0.49,1.11) | -2.38 (-2.61 , -2.16) |
| Uruguay | 68 (49,89) | 1.76 (1.27,2.29) | 73 (51,99) | 1.16 (0.82,1.57) | -1.46 (-1.58 , -1.34) |
| Uzbekistan | 48 (34,63) | 0.43 (0.3,0.56) | 67 (44,94) | 0.26 (0.17,0.37) | -1.77 (-2.06 , -1.48) |
| Vanuatu | 2 (1,2) | 3.18 (2.22,4.34) | 4 (3,6) | 2.79 (2,3.75) | -0.55 (-0.65 , -0.45) |
| Yemen | 82 (54,120) | 1.82 (1.24,2.58) | 252 (155,380) | 1.93 (1.21,2.89) | -0.04 (-0.15 , 0.06) |
| Zambia | 103 (75,133) | 3.88 (2.88,4.99) | 330 (198,663) | 5.17 (3.3,9.53) | 0.9 (0.82 , 0.98) |
| Zimbabwe | 131 (97,165) | 3.75 (2.78,4.71) | 310 (219,414) | 4.98 (3.62,6.45) | 1.29 (0.84 , 1.74) |
| ASMR, age-standardized mortality rate; UI, uncertainty interval; EAPC, estimated annual percentage change; CI, confidence interval. | | | | | |

| Table S2. The DALYs cases, age-standardized DALYs, and temporal trends of colon and rectum cancer attributable to diet low in calcium in 204 countries, 1990 and 2021 | | | | | |
| --- | --- | --- | --- | --- | --- |
|  | 1990 | 1990 | 2021 | 2021 | 1990-2021 |
| Location | DALYs  No. (95% UI) | ASDR per 100,000 No. (95% UI) | DALYs  No. (95% UI) | ASDR per 100,000 No. (95% UI) | EAPC  No. (95% CI) |
| Afghanistan | 4076 (1370,6972) | 57.64 (20.21,98.36) | 7367 (3019,12400) | 61.18 (27.34,101.21) | -0.22 (-0.5 , 0.05) |
| Albania | 144 (95,210) | 7.08 (4.69,10.33) | 150 (89,239) | 3.54 (2.09,5.66) | -2.21 (-2.57 , -1.85) |
| Algeria | 1172 (801,1543) | 10.2 (6.98,13.53) | 2032 (1298,2949) | 5.73 (3.65,8.33) | -1.97 (-2.13 , -1.81) |
| American Samoa | 9 (6,12) | 37.77 (26.97,50.57) | 20 (14,27) | 42.14 (29.61,57.17) | 0.4 (0.29 , 0.52) |
| Andorra | 5 (3,8) | 8.52 (5,13.56) | 9 (5,13) | 5.53 (3.03,8.64) | -1.08 (-1.29 , -0.86) |
| Angola | 2509 (1720,3523) | 58.86 (40.52,81.39) | 5633 (3606,7996) | 44 (29.09,61.9) | -1.31 (-1.45 , -1.17) |
| Antigua and Barbuda | 11 (8,14) | 19.7 (14.34,25.52) | 25 (18,32) | 23.6 (17.28,30.64) | 0.63 (0.45 , 0.8) |
| Argentina | 9616 (6876,12656) | 30.13 (21.56,39.64) | 13287 (9424,17621) | 23.96 (17.03,31.76) | -0.31 (-0.52 , -0.09) |
| Armenia | 758 (542,969) | 26.6 (19.02,33.91) | 538 (358,724) | 12.48 (8.3,16.76) | -2.87 (-3.1 , -2.64) |
| Australia | 3888 (2641,5213) | 20.16 (13.71,27.01) | 4595 (3025,6498) | 10.43 (6.93,14.69) | -2.46 (-2.63 , -2.29) |
| Austria | 2297 (1581,3086) | 19.32 (13.37,26.02) | 1301 (894,1815) | 7.12 (4.9,9.95) | -3.1 (-3.15 , -3.05) |
| Azerbaijan | 1163 (815,1528) | 21.85 (15.31,28.79) | 1144 (729,1663) | 10.54 (6.76,15.16) | -3.23 (-3.56 , -2.89) |
| Bahrain | 32 (22,42) | 17.19 (12.13,22.88) | 102 (63,152) | 11.51 (7.22,16.55) | -1.66 (-1.88 , -1.44) |
| Bangladesh | 24028 (17359,32546) | 46.57 (34.06,63.01) | 41081 (26876,61536) | 28.76 (18.99,42.72) | -1.65 (-1.76 , -1.54) |
| Barbados | 85 (62,108) | 29.61 (21.58,37.9) | 170 (116,233) | 33.47 (22.78,45.9) | 0.76 (0.49 , 1.04) |
| Belarus | 1929 (1314,2620) | 15.03 (10.27,20.43) | 2151 (1373,3153) | 13.55 (8.64,19.85) | -1.37 (-1.87 , -0.86) |
| Belgium | 2790 (1923,3782) | 18.12 (12.45,24.55) | 1889 (1288,2668) | 8.03 (5.49,11.2) | -2.42 (-2.57 , -2.26) |
| Belize | 10 (8,13) | 11.04 (7.97,14.04) | 49 (35,63) | 15.48 (11.12,20.17) | 1.2 (0.82 , 1.58) |
| Benin | 673 (495,853) | 33.18 (24.4,41.99) | 1383 (889,1989) | 26.26 (17.43,36.47) | -0.64 (-0.83 , -0.46) |
| Bermuda | 20 (14,27) | 33.02 (23.33,44.01) | 33 (22,45) | 24.08 (16.56,32.95) | -1.2 (-1.31 , -1.09) |
| Bhutan | 83 (50,119) | 30.15 (17.66,42.79) | 91 (58,124) | 14.56 (9.4,19.7) | -2.53 (-2.64 , -2.42) |
| Bolivarian Republic of Venezuela | 2398 (1770,2984) | 23.43 (17.37,29.17) | 7688 (4980,11232) | 25.63 (16.62,37.37) | -0.45 (-0.79 , -0.1) |
| Bosnia and Herzegovina | 977 (685,1262) | 23.57 (16.65,30.35) | 913 (601,1298) | 14.72 (9.62,20.88) | -1.69 (-1.86 , -1.52) |
| Botswana | 132 (82,185) | 23.65 (15.01,33.07) | 270 (169,418) | 18.12 (11.65,26.77) | -0.63 (-0.86 , -0.4) |
| Brazil | 18754 (14088,23477) | 20.33 (15.23,25.42) | 42131 (30132,55002) | 16.64 (11.91,21.71) | -0.91 (-1.03 , -0.78) |
| Brunei Darussalam | 53 (36,74) | 46.28 (31.4,63.27) | 107 (73,146) | 28.97 (19.59,39.21) | -1.05 (-1.41 , -0.68) |
| Bulgaria | 2708 (1881,3677) | 22.35 (15.46,30.16) | 3790 (2540,5241) | 27.55 (18.37,38.09) | 0.61 (0.2 , 1.03) |
| Burkina Faso | 1394 (981,1905) | 32.18 (22.95,43.41) | 2613 (1683,3618) | 27.89 (18.27,38.35) | -0.35 (-0.45 , -0.24) |
| Burundi | 1018 (684,1377) | 41.99 (28.32,56.38) | 2478 (1563,3798) | 47.25 (30.42,70.15) | -0.03 (-0.21 , 0.15) |
| Cambodia | 6520 (3948,9201) | 130.13 (80.28,181.17) | 15478 (10575,21300) | 117.5 (81.18,160.35) | -0.52 (-0.6 , -0.44) |
| Cameroon | 1834 (1339,2369) | 40.27 (29.48,51.8) | 4150 (2581,6171) | 31.99 (20.48,46.06) | -1.01 (-1.12 , -0.9) |
| Canada | 7347 (5250,9744) | 22.76 (16.28,30.27) | 8646 (5978,11867) | 12.41 (8.65,17.05) | -1.61 (-1.75 , -1.46) |
| Central African Republic | 663 (409,983) | 53.57 (34.62,78.89) | 1218 (714,1836) | 48.51 (29.59,71.95) | -0.37 (-0.54 , -0.19) |
| Chad | 702 (507,969) | 24.73 (17.95,34.13) | 1723 (1143,2463) | 28.83 (19.47,40.51) | 0.68 (0.61 , 0.76) |
| Chile | 2957 (2218,3682) | 29.63 (22.26,36.97) | 6065 (4428,7809) | 23.87 (17.44,30.69) | -0.1 (-0.31 , 0.11) |
| China | 546369 (389714,718067) | 61.59 (43.88,80.49) | 500468 (356219,682873) | 24.2 (17.14,33.05) | -3.18 (-3.28 , -3.08) |
| Colombia | 4470 (3326,5580) | 24.49 (18.25,30.62) | 11010 (7618,14717) | 20.02 (13.85,26.74) | -0.44 (-0.74 , -0.14) |
| Commonwealth of the Bahamas | 44 (32,56) | 26.83 (19.67,34.28) | 113 (76,156) | 27.48 (18.66,37.91) | 0.24 (0.14 , 0.34) |
| Comoros | 149 (94,206) | 70.93 (45.8,97.85) | 377 (236,544) | 74.27 (47.32,105.96) | 0.1 (-0.01 , 0.2) |
| Congo | 1004 (637,1375) | 88.93 (58.4,120.48) | 2146 (1454,2973) | 71.43 (49.96,96.74) | -0.93 (-1.09 , -0.77) |
| Cook Islands | 3 (2,4) | 24.63 (17.77,33.24) | 3 (2,4) | 11.23 (7.46,15.68) | -2.52 (-2.76 , -2.28) |
| Costa Rica | 301 (217,390) | 16.64 (12.05,21.57) | 1102 (755,1493) | 20.09 (13.8,27.17) | 0.79 (0.57 , 1) |
| Croatia | 1663 (1164,2211) | 28.43 (19.96,37.65) | 1626 (1116,2316) | 18.08 (12.31,25.82) | -1.86 (-2.09 , -1.62) |
| Cuba | 2797 (2058,3589) | 27.3 (20.1,35.07) | 5082 (3416,6725) | 26.3 (17.7,34.71) | -0.41 (-0.58 , -0.24) |
| Cyprus | 151 (103,207) | 20.78 (13.99,28.33) | 249 (164,337) | 12.7 (8.41,16.89) | -1.16 (-1.32 , -1) |
| Czech Republic | 4085 (2816,5460) | 29.59 (20.4,39.5) | 2932 (1948,4106) | 13.73 (9.12,19.3) | -2.83 (-3.03 , -2.63) |
| Democratic People's Republic of Korea | 10762 (7238,15783) | 62.71 (42.59,90.24) | 24331 (14993,38058) | 72.36 (44.86,112.72) | 0.67 (0.51 , 0.84) |
| Democratic Republic of the Congo | 7403 (5220,10157) | 45.16 (31.57,61.33) | 21815 (13455,32850) | 54.9 (34.46,82.66) | 0.65 (0.59 , 0.71) |
| Denmark | 1504 (1029,1997) | 18.76 (12.75,24.99) | 1182 (812,1678) | 9.78 (6.72,13.82) | -2.41 (-2.61 , -2.21) |
| Djibouti | 73 (46,106) | 48.42 (30.76,69.21) | 274 (163,421) | 40.55 (24.83,61.21) | -0.88 (-1 , -0.76) |
| Dominica | 10 (7,13) | 16.64 (11.52,22.49) | 11 (7,15) | 13.15 (8.7,18.91) | -0.62 (-0.8 , -0.44) |
| Dominican Republic | 1034 (751,1317) | 27.36 (19.77,34.97) | 2210 (1516,3030) | 21.8 (14.89,29.96) | -0.71 (-0.99 , -0.42) |
| Ecuador | 906 (664,1146) | 16.9 (12.41,21.36) | 3291 (2209,4533) | 20 (13.44,27.5) | 0.84 (0.44 , 1.23) |
| Egypt | 6775 (5082,8432) | 22.04 (16.51,27.72) | 10718 (7333,14783) | 15.83 (10.86,21.76) | -0.42 (-0.83 , -0.02) |
| El Salvador | 610 (460,758) | 19.58 (14.77,24.33) | 1028 (710,1413) | 16.63 (11.48,22.88) | -0.43 (-0.66 , -0.19) |
| Equatorial Guinea | 141 (91,196) | 67.96 (44.32,93.72) | 164 (98,252) | 29.37 (17.97,44.29) | -3.6 (-4.19 , -3.01) |
| Eritrea | 1009 (703,1334) | 75.99 (53.89,101.2) | 2213 (1436,3139) | 71.67 (47.73,100.3) | -0.1 (-0.15 , -0.04) |
| Estonia | 274 (188,370) | 13.39 (9.16,18.07) | 252 (164,354) | 9.17 (6.02,12.83) | -1.7 (-1.91 , -1.48) |
| Ethiopia | 37040 (20165,50171) | 178.34 (100.62,241.17) | 37833 (27008,48360) | 85.79 (61.56,108.97) | -2.91 (-3.13 , -2.69) |
| Federated States of Micronesia | 41 (28,56) | 80.53 (55.29,108.7) | 54 (36,77) | 69.33 (46.51,97.56) | -0.47 (-0.52 , -0.41) |
| Fiji | 133 (95,178) | 34.06 (24.12,44.75) | 225 (143,317) | 29.25 (18.79,41.05) | -0.09 (-0.38 , 0.2) |
| Finland | 657 (453,882) | 9.19 (6.33,12.43) | 704 (481,1007) | 5.6 (3.84,7.92) | -1.69 (-1.81 , -1.57) |
| France | 11245 (7749,15626) | 13.46 (9.34,18.63) | 12918 (8549,18285) | 9.02 (6.02,12.75) | -1.03 (-1.13 , -0.92) |
| Gabon | 449 (250,663) | 77.34 (43.4,114.1) | 622 (401,875) | 56.97 (36.98,79.75) | -1 (-1.09 , -0.91) |
| Georgia | 984 (681,1307) | 15.78 (10.93,20.93) | 900 (599,1218) | 15.59 (10.41,21.13) | 0.42 (0.12 , 0.73) |
| Germany | 29128 (20236,39355) | 22.58 (15.73,30.59) | 15722 (10320,21877) | 8.17 (5.42,11.34) | -3.81 (-4.05 , -3.57) |
| Ghana | 2236 (1581,2946) | 34.63 (24.85,45.18) | 3355 (2211,4441) | 20.06 (13.39,25.98) | -1.85 (-2 , -1.69) |
| Greece | 1565 (1085,2132) | 10.4 (7.2,14.17) | 1994 (1342,2760) | 8.04 (5.44,11.05) | -0.99 (-1.23 , -0.74) |
| Greenland | 13 (8,18) | 36.93 (24.69,51.44) | 10 (7,15) | 14.87 (9.54,21.43) | -3.31 (-3.47 , -3.15) |
| Grenada | 19 (13,24) | 26.7 (18.86,34.35) | 30 (21,40) | 27.48 (19.23,36.42) | 0.45 (0.34 , 0.57) |
| Guam | 19 (14,25) | 24.96 (18.07,32.6) | 37 (25,49) | 17.94 (12.48,24.08) | -0.61 (-0.93 , -0.29) |
| Guatemala | 941 (714,1150) | 25.78 (19.49,31.53) | 2846 (2013,3730) | 25 (17.71,32.72) | -0.52 (-0.81 , -0.22) |
| Guinea | 991 (729,1300) | 29.36 (21.73,38.46) | 1660 (1122,2385) | 28.06 (19.09,39.82) | -0.15 (-0.27 , -0.03) |
| Guinea-Bissau | 290 (183,390) | 68.51 (43.94,91.9) | 547 (374,744) | 68.6 (47.71,91.29) | 0.17 (0.11 , 0.23) |
| Guyana | 178 (131,227) | 45.09 (33.26,57.8) | 163 (109,231) | 24.84 (16.74,34.92) | -1.5 (-1.81 , -1.18) |
| Haiti | 3688 (2202,5352) | 109.6 (67.63,156.95) | 7021 (4434,10743) | 91.66 (58.17,139.71) | -0.58 (-0.65 , -0.51) |
| Honduras | 408 (291,528) | 18.02 (12.78,23.24) | 946 (634,1364) | 14.4 (9.62,20.59) | -0.58 (-0.77 , -0.39) |
| Hungary | 5258 (3566,7178) | 36.31 (24.64,49.31) | 5713 (3951,7882) | 30.27 (20.77,42) | -0.92 (-1.32 , -0.53) |
| Iceland | 26 (18,36) | 9.11 (6.22,12.54) | 32 (21,46) | 5.56 (3.64,7.84) | -1.78 (-1.92 , -1.63) |
| India | 85780 (64098,110507) | 16.88 (12.57,21.87) | 156174 (114449,199129) | 12.66 (9.28,16.13) | -0.98 (-1.08 , -0.88) |
| Indonesia | 98551 (71508,126166) | 89.27 (65.06,113.98) | 223404 (151813,300089) | 87.99 (60.75,117.07) | -0.12 (-0.26 , 0.03) |
| Iraq | 1941 (1385,2528) | 22.45 (16,29.44) | 7712 (5208,10741) | 29.71 (20.22,40.58) | 0.11 (-0.23 , 0.46) |
| Ireland | 597 (406,820) | 14.9 (10.14,20.37) | 512 (340,731) | 6.52 (4.34,9.31) | -2.47 (-2.58 , -2.36) |
| Islamic Republic of Iran | 6701 (4874,8630) | 24.6 (17.94,31.6) | 13221 (9503,17027) | 16.44 (11.84,21.13) | -0.67 (-1.01 , -0.33) |
| Israel | 823 (568,1102) | 17.15 (11.87,22.97) | 939 (634,1342) | 7.54 (5.06,10.78) | -2.97 (-3.18 , -2.75) |
| Italy | 14473 (10159,19265) | 16.57 (11.61,22.03) | 14112 (9550,19517) | 9.68 (6.76,13.31) | -1.68 (-1.74 , -1.62) |
| Jamaica | 344 (249,444) | 19.27 (13.93,24.82) | 762 (505,1087) | 24.63 (16.31,35.13) | 0.99 (0.66 , 1.33) |
| Japan | 51542 (37638,65945) | 30.68 (22.43,39.2) | 80619 (56700,106158) | 23.62 (17.04,31.02) | -0.67 (-0.77 , -0.58) |
| Jordan | 440 (310,591) | 29.7 (21.07,39.25) | 1282 (788,1971) | 16.01 (9.87,24.4) | -2.4 (-2.59 , -2.21) |
| Kazakhstan | 2356 (1663,3148) | 18.08 (12.72,24.2) | 1250 (832,1729) | 6.88 (4.58,9.52) | -3.56 (-3.96 , -3.15) |
| Kenya | 1158 (824,1576) | 13.06 (9.38,17.72) | 3341 (2278,4718) | 13.7 (9.33,19.25) | 0.45 (0.3 , 0.59) |
| Kingdom of Eswatini | 175 (116,245) | 58.37 (38.58,81.06) | 409 (252,599) | 67 (42.16,97.28) | 0.8 (0.31 , 1.28) |
| Kiribati | 33 (24,43) | 80.79 (59.69,104.75) | 56 (39,78) | 70.47 (50.7,96.77) | -0.61 (-0.67 , -0.55) |
| Kuwait | 46 (32,62) | 6.81 (4.72,9.28) | 218 (140,308) | 6.32 (4.05,8.89) | 0.14 (-0.12 , 0.42) |
| Kyrgyzstan | 407 (289,546) | 13.32 (9.46,17.91) | 336 (221,466) | 6.65 (4.37,9.24) | -2.56 (-2.76 , -2.37) |
| Lao People's Democratic Republic | 3125 (1577,4575) | 137.39 (71.33,199.28) | 5101 (3302,7122) | 100.71 (66.35,139.1) | -1.25 (-1.35 , -1.15) |
| Latvia | 475 (324,644) | 13.32 (9.08,18.07) | 405 (262,569) | 10.23 (6.6,14.36) | -1.25 (-1.67 , -0.83) |
| Lebanon | 355 (225,498) | 16.55 (10.49,23.21) | 668 (434,963) | 10.88 (7.05,15.72) | -0.88 (-1.11 , -0.66) |
| Lesotho | 455 (300,626) | 53.52 (35.57,73.33) | 1189 (743,1779) | 104.95 (65.75,155.5) | 2.97 (2.48 , 3.46) |
| Liberia | 416 (299,559) | 35.71 (25.99,47.43) | 998 (580,1637) | 43.55 (25.96,70.18) | 0.71 (0.58 , 0.84) |
| Libya | 430 (288,599) | 21.69 (14.6,29.95) | 1405 (883,2054) | 24.52 (15.66,35.13) | 0.54 (0.39 , 0.68) |
| Lithuania | 825 (584,1100) | 18.46 (13.06,24.62) | 641 (422,904) | 11.12 (7.29,15.71) | -2.11 (-2.32 , -1.91) |
| Luxembourg | 86 (58,116) | 15.99 (10.76,21.59) | 74 (48,103) | 6.73 (4.45,9.35) | -2.67 (-2.85 , -2.48) |
| Madagascar | 2167 (1521,2867) | 40.27 (28.63,53.02) | 6027 (4040,8469) | 48.17 (32.32,66.73) | 0.53 (0.46 , 0.6) |
| Malawi | 1581 (1162,2038) | 38.24 (28.37,49.02) | 3048 (2107,4487) | 37.7 (26.58,53.82) | -0.33 (-0.53 , -0.13) |
| Malaysia | 8963 (6885,11394) | 91.87 (70.69,116.84) | 19305 (14600,24362) | 67.59 (51.82,85.53) | -0.94 (-1.07 , -0.82) |
| Maldives | 38 (20,53) | 39.33 (22.85,53.91) | 30 (20,41) | 8.49 (5.74,11.59) | -5.54 (-5.88 , -5.19) |
| Mali | 1276 (943,1633) | 31.3 (23.51,39.76) | 1238 (774,1752) | 13.59 (8.7,18.83) | -2.93 (-3.35 , -2.5) |
| Malta | 78 (55,106) | 18.44 (12.95,25.05) | 95 (62,132) | 9.74 (6.41,13.52) | -1.91 (-2.11 , -1.72) |
| Marshall Islands | 13 (9,17) | 72.2 (49.65,96.62) | 25 (17,36) | 65.38 (44.13,91.8) | -0.3 (-0.38 , -0.22) |
| Mauritania | 92 (60,126) | 9.24 (6.17,12.61) | 200 (126,290) | 9.25 (5.81,13.32) | -0.05 (-0.16 , 0.06) |
| Mauritius | 143 (109,178) | 19.16 (14.37,23.89) | 375 (266,487) | 20.85 (14.83,27.08) | -0.18 (-0.45 , 0.09) |
| Mexico | 5617 (4219,6999) | 12.76 (9.56,15.88) | 20498 (15117,26325) | 15.77 (11.65,20.24) | 0.72 (0.5 , 0.93) |
| Mongolia | 213 (139,294) | 18.89 (12.4,26.18) | 329 (215,453) | 13.08 (8.48,18.06) | -1.67 (-1.86 , -1.49) |
| Montenegro | 44 (29,63) | 7.1 (4.67,10.01) | 75 (48,110) | 7.85 (5.01,11.51) | 0.19 (-0.02 , 0.4) |
| Morocco | 4110 (2770,5603) | 27.38 (18.9,37.32) | 6596 (4100,9530) | 18.66 (11.79,26.93) | -1.37 (-1.48 , -1.26) |
| Mozambique | 1492 (1110,1913) | 26.07 (19.32,33.09) | 3056 (1977,4126) | 28.49 (18.99,37.73) | 0.66 (0.53 , 0.78) |
| Myanmar | 29973 (17434,44696) | 117.79 (70.35,173.62) | 35204 (23474,48133) | 69.81 (46.82,94.95) | -2.23 (-2.45 , -2.01) |
| Namibia | 242 (180,311) | 35.32 (26.41,45.49) | 446 (298,624) | 30.23 (20.64,41.71) | -0.74 (-0.94 , -0.54) |
| Nepal | 3023 (1910,4433) | 28.91 (18.61,42.1) | 3929 (2612,5478) | 16.32 (10.94,22.72) | -2 (-2.32 , -1.68) |
| Netherlands | 2631 (1749,3587) | 13.26 (8.82,18.08) | 3388 (2255,4717) | 9.84 (6.64,13.63) | -0.84 (-1.04 , -0.63) |
| New Zealand | 1347 (951,1807) | 35.09 (24.81,47.08) | 2070 (1439,2759) | 25.05 (17.52,33.31) | -1.11 (-1.33 , -0.89) |
| Nicaragua | 377 (288,474) | 22.51 (17.06,28.03) | 873 (605,1192) | 17.11 (11.85,23.15) | -1.03 (-1.16 , -0.89) |
| Niger | 598 (418,843) | 20.96 (14.72,29.27) | 1037 (676,1496) | 12.91 (8.54,18.23) | -1.77 (-2.13 , -1.41) |
| Nigeria | 16232 (11296,21885) | 36 (25.27,48.35) | 24210 (16476,32966) | 25.84 (18.06,34.68) | -1.16 (-1.23 , -1.09) |
| Northern Mariana Islands | 6 (4,8) | 26.61 (18.05,37.08) | 15 (11,20) | 29.12 (20.33,37.33) | 0.51 (0.18 , 0.84) |
| Norway | 1022 (699,1366) | 15.25 (10.44,20.34) | 1030 (692,1428) | 10.01 (6.76,13.85) | -1.25 (-1.35 , -1.15) |
| Oman | 58 (37,84) | 8 (5.24,11.47) | 77 (47,115) | 3.67 (2.34,5.42) | -2.37 (-2.52 , -2.22) |
| Pakistan | 10865 (7951,14460) | 18.5 (13.62,24.75) | 19277 (13194,26660) | 14.45 (10,19.88) | -0.91 (-0.98 , -0.85) |
| Palestine | 896 (596,1241) | 101 (67.87,139.04) | 2156 (1546,2790) | 81.02 (58.04,104.68) | -0.51 (-0.73 , -0.28) |
| Panama | 515 (389,631) | 33.54 (25.36,41.16) | 990 (680,1365) | 22.38 (15.34,30.85) | -1.47 (-1.57 , -1.38) |
| Papua New Guinea | 531 (353,756) | 25.36 (17.37,35.81) | 1498 (1034,2013) | 24.83 (17.31,33.13) | -0.06 (-0.14 , 0.03) |
| Paraguay | 327 (228,430) | 14.28 (9.96,18.64) | 1070 (706,1514) | 18.11 (12.01,25.63) | 1.13 (0.98 , 1.28) |
| Peru | 3312 (2421,4374) | 26.93 (19.72,35.53) | 5808 (3775,8396) | 17.17 (11.17,24.81) | -1.97 (-2.26 , -1.69) |
| Philippines | 31143 (23819,38790) | 91.07 (69.23,114.83) | 91059 (68799,115157) | 101.64 (76.87,127.98) | 0.49 (0.44 , 0.55) |
| Plurinational State of Bolivia | 3069 (1859,4363) | 92.75 (56.91,131.66) | 6629 (4171,9744) | 72.53 (45.97,105.93) | -0.94 (-0.99 , -0.9) |
| Poland | 8594 (6016,11321) | 19.78 (13.85,26.03) | 12839 (8871,17530) | 17.89 (12.38,24.43) | -0.58 (-0.99 , -0.16) |
| Portugal | 3716 (2640,4957) | 27.79 (19.66,36.94) | 3205 (2197,4373) | 13.13 (9.02,17.91) | -2.08 (-2.19 , -1.97) |
| Principality of Monaco | 9 (6,13) | 14.13 (8.71,20.47) | 15 (10,21) | 16.32 (10.51,23.62) | 0.5 (0.46 , 0.54) |
| Puerto Rico | 802 (575,1058) | 22.45 (16.08,29.56) | 1235 (846,1668) | 18.83 (12.73,25.68) | -0.67 (-0.8 , -0.54) |
| Qatar | 17 (12,24) | 13.94 (9.52,19.39) | 66 (39,103) | 7.3 (4.38,10.98) | -2.39 (-2.84 , -1.95) |
| Republic of Cabo Verde | 24 (18,32) | 10.58 (7.71,13.82) | 58 (39,80) | 13.24 (8.94,18.32) | 0.06 (-0.3 , 0.42) |
| Republic of Côte d'Ivoire | 1098 (774,1432) | 25.68 (18.28,32.73) | 2505 (1652,3692) | 20.86 (14.33,30.01) | -0.81 (-0.96 , -0.67) |
| Republic of Korea | 9297 (6633,11751) | 30.56 (21.93,38.52) | 16262 (10936,22473) | 17.83 (12.01,24.7) | -1.88 (-2.06 , -1.69) |
| Republic of Moldova | 1183 (848,1531) | 26.4 (18.96,34.1) | 1358 (935,1899) | 22.84 (15.74,31.91) | -0.34 (-0.64 , -0.04) |
| Republic of Nauru | 3 (1,4) | 55.27 (31.68,83.2) | 4 (2,6) | 61.42 (35.87,87.15) | 0.19 (-0.45 , 0.83) |
| Republic of Niue | 1 (1,1) | 47.8 (34.33,63.86) | 1 (1,1) | 37.22 (25.58,50.03) | -1.1 (-1.2 , -1) |
| Republic of Palau | 5 (4,8) | 55.72 (38.28,76.58) | 8 (5,11) | 39.54 (26.39,53.73) | -0.9 (-1.03 , -0.78) |
| Republic of San Marino | 4 (3,6) | 11.87 (7.38,17.35) | 4 (2,7) | 5.52 (3.1,8.71) | -1.7 (-1.98 , -1.42) |
| Republic of the Gambia | 74 (51,96) | 19.98 (13.96,25.89) | 183 (119,255) | 17.76 (11.65,24.6) | -0.65 (-0.86 , -0.44) |
| Romania | 3537 (2379,4886) | 12.79 (8.62,17.67) | 3737 (2439,5292) | 10.43 (6.8,14.64) | -1.3 (-1.63 , -0.96) |
| Russian Federation | 47890 (35092,61792) | 26.51 (19.45,34.13) | 45768 (30961,60334) | 19.32 (13.08,25.44) | -1.82 (-2.15 , -1.49) |
| Rwanda | 1560 (1049,2163) | 51.35 (35.16,70.59) | 1784 (1066,2621) | 27.73 (16.6,40.14) | -3.37 (-3.83 , -2.91) |
| Saint Kitts and Nevis | 16 (11,20) | 42.77 (31.05,55) | 20 (14,27) | 30.52 (21.55,40.61) | -0.55 (-0.72 , -0.39) |
| Saint Lucia | 21 (16,27) | 25.14 (18.49,31.91) | 43 (31,59) | 18.25 (12.85,24.75) | -1.14 (-1.39 , -0.88) |
| Saint Vincent and the Grenadines | 33 (25,41) | 46.33 (34.61,57.49) | 34 (24,44) | 24.36 (17.34,31.95) | -2.44 (-2.85 , -2.03) |
| Samoa | 35 (25,47) | 41.64 (29.58,54.09) | 43 (29,58) | 29.62 (20.1,39.55) | -1.54 (-1.81 , -1.28) |
| Sao Tome and Principe | 34 (25,45) | 53.68 (39.01,69.09) | 56 (39,76) | 50.81 (36.15,69.29) | -0.13 (-0.2 , -0.06) |
| Saudi Arabia | 717 (472,1043) | 10.6 (7.09,15) | 2762 (1692,3947) | 10.52 (6.79,14.6) | 0.2 (-0.17 , 0.57) |
| Senegal | 1202 (875,1560) | 36.44 (26.41,47.17) | 2472 (1666,3406) | 31.88 (21.61,43.8) | -0.54 (-0.68 , -0.39) |
| Serbia | 3059 (2031,4396) | 29.02 (19.45,41.57) | 3665 (2371,5280) | 22.53 (14.46,32.55) | -1.29 (-1.54 , -1.04) |
| Seychelles | 31 (24,40) | 55.26 (41.74,69.94) | 51 (37,66) | 43.78 (31.94,56.98) | -0.46 (-0.6 , -0.31) |
| Sierra Leone | 789 (556,1070) | 37.98 (27.01,51.04) | 1584 (1022,2189) | 40.21 (26.36,55.14) | 0.46 (0.34 , 0.58) |
| Singapore | 894 (655,1141) | 38.96 (28.56,50.09) | 1303 (940,1755) | 15.3 (11.05,20.52) | -3.11 (-3.32 , -2.91) |
| Slovakia | 2438 (1727,3229) | 40.79 (28.97,53.8) | 2996 (1968,4173) | 31.78 (20.85,44.33) | -0.9 (-1.08 , -0.72) |
| Slovenia | 452 (310,623) | 18.33 (12.56,25.23) | 370 (242,536) | 8.1 (5.27,11.82) | -2.97 (-3.16 , -2.77) |
| Socialist Republic of Viet Nam | 33754 (23809,43579) | 81.12 (57.36,104.78) | 83464 (56460,109635) | 80.36 (54.98,104.67) | -0.16 (-0.26 , -0.07) |
| Solomon Islands | 67 (35,100) | 45.98 (25.97,66.4) | 159 (102,232) | 41.13 (27.44,58.85) | -0.31 (-0.43 , -0.19) |
| Somalia | 2079 (1242,3183) | 74.29 (45.26,112.28) | 5384 (3178,8401) | 77.34 (46.35,116.68) | 0.1 (0.03 , 0.18) |
| South Africa | 9776 (7267,13118) | 43.92 (32.44,59.84) | 25351 (19381,31320) | 52.74 (40.12,65.04) | 0.56 (0.29 , 0.84) |
| South Sudan | 946 (594,1355) | 35.97 (22.75,51.34) | 1579 (1005,2241) | 38.24 (24.58,54.15) | 0.02 (-0.12 , 0.16) |
| Spain | 10997 (7715,14579) | 20.72 (14.5,27.48) | 15474 (10685,21277) | 15.77 (10.92,21.71) | -0.49 (-0.69 , -0.3) |
| Sri Lanka | 3562 (2795,4354) | 31.4 (24.5,38.5) | 5167 (3142,7478) | 19.21 (11.72,27.7) | -1.38 (-1.59 , -1.17) |
| Sudan | 3221 (2025,4763) | 32.14 (20.49,46.67) | 5445 (3322,8614) | 24.54 (15.27,38.53) | -1 (-1.05 , -0.95) |
| Suriname | 84 (62,108) | 32.71 (24.13,41.78) | 235 (160,327) | 37.05 (25.31,51.31) | 0.45 (0.04 , 0.86) |
| Sweden | 1569 (1064,2160) | 10.66 (7.26,14.55) | 1502 (985,2109) | 6.94 (4.61,9.76) | -1.24 (-1.38 , -1.1) |
| Switzerland | 870 (595,1199) | 8.38 (5.75,11.54) | 982 (639,1375) | 5.34 (3.47,7.4) | -1.48 (-1.65 , -1.3) |
| Syrian Arab Republic | 797 (540,1114) | 13.79 (9.39,18.88) | 1562 (1000,2287) | 11.78 (7.57,17) | -0.8 (-1.12 , -0.47) |
| Taiwan (Province of China) | 4359 (3223,5675) | 26.34 (19.41,34.29) | 11368 (8063,14970) | 27.76 (19.79,36.48) | 0.16 (-0.11 , 0.43) |
| Tajikistan | 843 (606,1095) | 28.87 (20.73,37.59) | 1233 (801,1798) | 18.77 (12.44,26.1) | -2.03 (-2.42 , -1.63) |
| Thailand | 39665 (29215,49958) | 104.45 (76.89,132.12) | 80207 (54383,109142) | 76.59 (51.86,103.94) | -1.27 (-1.43 , -1.12) |
| The former Yugoslav Republic of Macedonia | 447 (315,604) | 23.73 (16.75,32.08) | 568 (367,826) | 17.96 (11.65,25.87) | -1.32 (-1.69 , -0.96) |
| Timor-Leste | 261 (170,360) | 79.27 (53.43,107.21) | 688 (471,917) | 78.14 (53.76,104.57) | -0.05 (-0.24 , 0.14) |
| Togo | 502 (354,668) | 38.44 (27.32,50.83) | 1712 (1077,2512) | 42.84 (27.56,61.16) | 0.42 (0.37 , 0.47) |
| Tokelau | 1 (1,1) | 63.19 (43.5,84.88) | 1 (0,1) | 40.07 (27.63,55.31) | -1.59 (-1.63 , -1.54) |
| Tonga | 22 (16,28) | 38.8 (28.02,49.56) | 26 (18,36) | 32.33 (22.51,44.24) | -0.58 (-0.67 , -0.5) |
| Trinidad and Tobago | 226 (165,289) | 27.22 (19.96,34.66) | 427 (286,616) | 22.4 (15.06,32.26) | -1.15 (-1.36 , -0.93) |
| Tunisia | 728 (502,981) | 14.5 (10.05,19.45) | 1275 (803,1905) | 9.58 (6.04,14.31) | -1.61 (-1.7 , -1.51) |
| Turkey | 7052 (4706,9868) | 19.3 (13.08,26.98) | 9949 (6340,14235) | 10.76 (6.88,15.26) | -1.98 (-2.11 , -1.84) |
| Turkmenistan | 244 (179,315) | 11.71 (8.61,15.1) | 279 (174,416) | 6.46 (4.03,9.6) | -2.3 (-2.66 , -1.93) |
| Tuvalu | 5 (4,8) | 78.02 (55.76,108.49) | 6 (4,8) | 57.86 (40.9,79.71) | -0.86 (-0.94 , -0.79) |
| Uganda | 2573 (1797,3487) | 39.14 (27.7,52.19) | 5810 (3747,8102) | 36.71 (24.44,50.09) | -0.84 (-1.11 , -0.56) |
| Ukraine | 18180 (12609,24127) | 25.56 (17.74,34) | 14379 (8817,21151) | 18.93 (11.58,28.06) | -1.73 (-2.1 , -1.36) |
| United Arab Emirates | 73 (40,116) | 13.97 (8.15,21.22) | 482 (298,756) | 14.16 (8.98,21.1) | 2.16 (1.51 , 2.81) |
| United Kingdom of Great Britain and Northern Ireland | 18072 (12708,24186) | 20.26 (14.23,27.02) | 12343 (8615,16658) | 9.62 (6.69,12.96) | -2.31 (-2.62 , -2) |
| United Republic of Tanzania | 6800 (4883,8997) | 59.64 (43.16,78.93) | 12826 (8324,17706) | 47.7 (31.39,65.06) | -0.97 (-1.06 , -0.87) |
| United States of America | 51220 (35631,69049) | 16.1 (11.22,21.71) | 49961 (34653,67588) | 9.06 (6.33,12.23) | -1.7 (-1.84 , -1.57) |
| United States Virgin Islands | 30 (21,39) | 35.35 (24.64,46.78) | 27 (17,40) | 16.23 (10.32,24.18) | -2.35 (-2.59 , -2.12) |
| Uruguay | 1397 (1011,1833) | 36.44 (26.35,47.73) | 1269 (892,1730) | 22.9 (15.99,31.42) | -1.6 (-1.71 , -1.49) |
| Uzbekistan | 1378 (986,1799) | 11.35 (8.08,14.79) | 1997 (1302,2777) | 6.84 (4.49,9.52) | -1.94 (-2.25 , -1.63) |
| Vanuatu | 52 (34,72) | 75.82 (50.64,104.51) | 126 (88,173) | 66.38 (46.54,89.76) | -0.61 (-0.74 , -0.47) |
| Yemen | 2396 (1512,3607) | 44.7 (29.3,65.63) | 7243 (4310,11048) | 45.89 (28.1,69.26) | -0.17 (-0.29 , -0.05) |
| Zambia | 2987 (2178,3862) | 95.62 (70.19,123.71) | 9870 (5701,21177) | 123.81 (74.17,249.01) | 0.76 (0.67 , 0.85) |
| Zimbabwe | 3394 (2498,4338) | 82.18 (61.05,104.06) | 9056 (6202,12492) | 118.33 (83.26,158.87) | 1.59 (1.06 , 2.12) |
| DALYs, disability-adjusted life-years; ASDR, age-standardized DALY rate; UI, uncertainty interval; EAPC, estimated annual percentage change; CI, confidence interval. | | | | | |

| Table S3. The deaths cases, age-standardized deaths, and temporal trends of prostate cancer attributable to diet low in calcium in 204 countries, 1990 and 2021 | | | | |
| --- | --- | --- | --- | --- |
|  | 1990 | 1990 | 2021 | 2021 |
| Location | Deaths cases  No. (95% UI) | ASMR per 100,000  No. (95% UI) | Deaths cases  No. (95% UI) | ASMR per 100,000  No. (95% UI) |
| Afghanistan | -6 (-13,1) | -0.11 (-0.27,0.02) | -7 (-17,1) | -0.12 (-0.28,0.02) |
| Albania | 0 (-1,0) | -0.03 (-0.07,0) | 0 (-1,0) | -0.01 (-0.02,0) |
| Algeria | -2 (-4,0) | -0.03 (-0.07,0.01) | -3 (-8,1) | -0.01 (-0.03,0) |
| American Samoa | 0 (0,0) | -0.47 (-1.08,0.09) | 0 (0,0) | -0.54 (-1.21,0.09) |
| Andorra | 0 (0,0) | -0.02 (-0.05,0) | 0 (0,0) | -0.01 (-0.04,0) |
| Angola | -9 (-21,2) | -0.4 (-0.93,0.08) | -26 (-59,5) | -0.36 (-0.84,0.07) |
| Antigua and Barbuda | 0 (0,0) | -0.32 (-0.7,0.06) | 0 (-1,0) | -0.35 (-0.73,0.06) |
| Argentina | -29 (-64,6) | -0.1 (-0.21,0.02) | -35 (-80,7) | -0.06 (-0.13,0.01) |
| Armenia | -1 (-3,0) | -0.05 (-0.11,0.01) | -1 (-2,0) | -0.02 (-0.05,0) |
| Australia | -10 (-24,2) | -0.05 (-0.12,0.01) | -13 (-31,2) | -0.02 (-0.06,0) |
| Austria | -4 (-9,1) | -0.03 (-0.07,0) | -3 (-8,1) | -0.02 (-0.04,0) |
| Azerbaijan | -3 (-6,0) | -0.06 (-0.14,0.01) | -2 (-5,0) | -0.03 (-0.06,0) |
| Bahrain | 0 (0,0) | -0.09 (-0.21,0.02) | 0 (-1,0) | -0.06 (-0.16,0.01) |
| Bangladesh | -58 (-135,8) | -0.15 (-0.35,0.02) | -141 (-397,20) | -0.12 (-0.34,0.02) |
| Barbados | -1 (-1,0) | -0.21 (-0.43,0.04) | -1 (-3,0) | -0.23 (-0.52,0.04) |
| Belarus | -1 (-3,0) | -0.01 (-0.02,0) | -3 (-6,0) | -0.02 (-0.04,0) |
| Belgium | -5 (-13,1) | -0.03 (-0.08,0.01) | -4 (-10,1) | -0.01 (-0.03,0) |
| Belize | 0 (0,0) | -0.13 (-0.27,0.03) | 0 (-1,0) | -0.2 (-0.43,0.03) |
| Benin | -5 (-13,1) | -0.32 (-0.76,0.06) | -14 (-33,3) | -0.37 (-0.84,0.07) |
| Bermuda | 0 (0,0) | -0.14 (-0.3,0.02) | 0 (-1,0) | -0.16 (-0.36,0.03) |
| Bhutan | 0 (0,0) | -0.08 (-0.2,0.01) | 0 (-1,0) | -0.07 (-0.18,0.01) |
| Bolivarian Republic of Venezuela | -17 (-36,3) | -0.21 (-0.45,0.04) | -71 (-153,12) | -0.26 (-0.57,0.04) |
| Bosnia and Herzegovina | -2 (-4,0) | -0.06 (-0.14,0.01) | -2 (-4,0) | -0.02 (-0.06,0) |
| Botswana | -1 (-3,0) | -0.29 (-0.67,0.06) | -2 (-6,0) | -0.23 (-0.52,0.04) |
| Brazil | -110 (-232,21) | -0.16 (-0.33,0.03) | -175 (-384,32) | -0.07 (-0.16,0.01) |
| Brunei Darussalam | 0 (0,0) | -0.08 (-0.18,0.01) | 0 (0,0) | -0.05 (-0.12,0.01) |
| Bulgaria | -3 (-7,1) | -0.03 (-0.07,0.01) | -8 (-17,1) | -0.05 (-0.11,0.01) |
| Burkina Faso | -9 (-21,1) | -0.28 (-0.67,0.05) | -25 (-60,5) | -0.36 (-0.86,0.07) |
| Burundi | -5 (-13,1) | -0.29 (-0.69,0.06) | -12 (-31,2) | -0.36 (-0.87,0.06) |
| Cambodia | -6 (-14,1) | -0.19 (-0.44,0.04) | -20 (-46,4) | -0.22 (-0.5,0.04) |
| Cameroon | -11 (-25,2) | -0.34 (-0.8,0.06) | -36 (-82,7) | -0.41 (-0.95,0.08) |
| Canada | -24 (-54,5) | -0.07 (-0.17,0.01) | -20 (-47,4) | -0.02 (-0.06,0) |
| Central African Republic | -2 (-5,0) | -0.3 (-0.67,0.06) | -4 (-9,1) | -0.29 (-0.69,0.06) |
| Chad | -5 (-13,1) | -0.22 (-0.53,0.04) | -17 (-39,3) | -0.41 (-0.95,0.07) |
| Chile | -18 (-39,3) | -0.21 (-0.44,0.04) | -39 (-85,7) | -0.15 (-0.32,0.03) |
| China | -261 (-584,43) | -0.05 (-0.11,0.01) | -496 (-1164,92) | -0.03 (-0.06,0.01) |
| Colombia | -28 (-59,5) | -0.2 (-0.42,0.04) | -57 (-125,9) | -0.1 (-0.23,0.02) |
| Commonwealth of the Bahamas | 0 (-1,0) | -0.24 (-0.52,0.04) | -1 (-2,0) | -0.28 (-0.61,0.05) |
| Comoros | -1 (-2,0) | -0.42 (-1.03,0.07) | -2 (-4,0) | -0.46 (-1.1,0.08) |
| Congo | -3 (-7,1) | -0.49 (-1.03,0.09) | -9 (-19,2) | -0.53 (-1.15,0.1) |
| Cook Islands | 0 (0,0) | -0.79 (-1.82,0.14) | 0 (0,0) | -0.46 (-1.02,0.08) |
| Costa Rica | -2 (-3,0) | -0.1 (-0.22,0.02) | -4 (-8,1) | -0.07 (-0.15,0.01) |
| Croatia | -4 (-8,1) | -0.07 (-0.15,0.01) | -3 (-8,1) | -0.03 (-0.08,0.01) |
| Cuba | -19 (-40,4) | -0.2 (-0.42,0.04) | -33 (-68,6) | -0.15 (-0.32,0.03) |
| Cyprus | -1 (-2,0) | -0.13 (-0.32,0.02) | -1 (-3,0) | -0.06 (-0.15,0.01) |
| Czech Republic | -4 (-10,1) | -0.03 (-0.07,0.01) | -5 (-12,1) | -0.02 (-0.05,0) |
| Democratic People's Republic of Korea | -5 (-12,1) | -0.05 (-0.1,0.01) | -17 (-38,3) | -0.06 (-0.12,0.01) |
| Democratic Republic of the Congo | -35 (-82,7) | -0.4 (-0.92,0.08) | -100 (-230,17) | -0.42 (-0.99,0.07) |
| Denmark | -4 (-8,1) | -0.04 (-0.09,0.01) | -3 (-7,1) | -0.02 (-0.05,0) |
| Djibouti | 0 (-1,0) | -0.35 (-0.85,0.07) | -1 (-3,0) | -0.34 (-0.8,0.06) |
| Dominica | 0 (0,0) | -0.14 (-0.33,0.02) | 0 (0,0) | -0.08 (-0.21,0.01) |
| Dominican Republic | -11 (-30,2) | -0.39 (-1.06,0.06) | -27 (-67,5) | -0.28 (-0.71,0.05) |
| Ecuador | -10 (-20,2) | -0.22 (-0.47,0.04) | -27 (-56,5) | -0.18 (-0.38,0.03) |
| Egypt | -10 (-28,2) | -0.06 (-0.18,0.01) | -23 (-53,4) | -0.07 (-0.16,0.01) |
| El Salvador | -5 (-15,1) | -0.19 (-0.54,0.03) | -9 (-21,2) | -0.14 (-0.31,0.02) |
| Equatorial Guinea | -1 (-1,0) | -0.4 (-0.88,0.07) | -1 (-2,0) | -0.28 (-0.63,0.06) |
| Eritrea | -2 (-5,1) | -0.3 (-0.72,0.07) | -6 (-14,1) | -0.31 (-0.75,0.07) |
| Estonia | 0 (-1,0) | -0.01 (-0.03,0) | -1 (-1,0) | -0.02 (-0.04,0) |
| Ethiopia | -26 (-64,4) | -0.18 (-0.45,0.03) | -50 (-126,6) | -0.15 (-0.38,0.02) |
| Federated States of Micronesia | 0 (0,0) | -0.38 (-0.93,0.06) | 0 (0,0) | -0.4 (-0.91,0.07) |
| Fiji | -1 (-1,0) | -0.24 (-0.62,0.05) | -1 (-3,0) | -0.2 (-0.51,0.05) |
| Finland | -1 (-3,0) | -0.02 (-0.04,0) | -2 (-4,0) | -0.01 (-0.03,0) |
| France | -23 (-54,4) | -0.02 (-0.06,0) | -28 (-65,5) | -0.02 (-0.03,0) |
| Gabon | -2 (-4,0) | -0.4 (-0.88,0.07) | -3 (-6,1) | -0.38 (-0.88,0.08) |
| Georgia | -2 (-4,0) | -0.03 (-0.07,0.01) | -3 (-8,1) | -0.06 (-0.13,0.01) |
| Germany | -42 (-95,7) | -0.03 (-0.07,0.01) | -36 (-83,6) | -0.01 (-0.03,0) |
| Ghana | -25 (-57,4) | -0.59 (-1.35,0.1) | -40 (-89,8) | -0.33 (-0.74,0.06) |
| Greece | -3 (-8,1) | -0.02 (-0.05,0) | -6 (-14,1) | -0.02 (-0.04,0) |
| Greenland | 0 (0,0) | -0.02 (-0.04,0) | 0 (0,0) | -0.01 (-0.02,0) |
| Grenada | 0 (-1,0) | -0.29 (-0.61,0.05) | 0 (-1,0) | -0.31 (-0.67,0.06) |
| Guam | 0 (0,0) | -0.12 (-0.28,0.02) | 0 (0,0) | -0.06 (-0.14,0.01) |
| Guatemala | -5 (-11,1) | -0.24 (-0.5,0.05) | -25 (-54,5) | -0.26 (-0.56,0.05) |
| Guinea | -8 (-18,1) | -0.28 (-0.66,0.05) | -14 (-31,3) | -0.32 (-0.71,0.06) |
| Guinea-Bissau | -1 (-3,0) | -0.41 (-0.95,0.08) | -3 (-6,1) | -0.56 (-1.3,0.11) |
| Guyana | -2 (-4,0) | -0.54 (-1.15,0.1) | -2 (-4,0) | -0.33 (-0.72,0.06) |
| Haiti | -18 (-43,3) | -0.81 (-1.93,0.14) | -44 (-100,9) | -0.95 (-2.16,0.19) |
| Honduras | -3 (-6,1) | -0.18 (-0.4,0.03) | -8 (-19,2) | -0.16 (-0.39,0.03) |
| Hungary | -8 (-18,1) | -0.05 (-0.12,0.01) | -9 (-21,2) | -0.04 (-0.09,0.01) |
| Iceland | 0 (0,0) | -0.02 (-0.05,0) | 0 (0,0) | -0.01 (-0.03,0) |
| India | -175 (-406,36) | -0.06 (-0.13,0.01) | -489 (-1094,85) | -0.05 (-0.12,0.01) |
| Indonesia | -114 (-258,20) | -0.16 (-0.38,0.03) | -360 (-814,63) | -0.22 (-0.5,0.04) |
| Iraq | -4 (-11,1) | -0.06 (-0.16,0.01) | -15 (-36,3) | -0.09 (-0.22,0.02) |
| Ireland | -1 (-2,0) | -0.02 (-0.05,0) | -1 (-2,0) | -0.01 (-0.03,0) |
| Islamic Republic of Iran | -18 (-41,3) | -0.11 (-0.25,0.02) | -63 (-137,12) | -0.1 (-0.22,0.02) |
| Israel | -1 (-3,0) | -0.03 (-0.06,0) | -1 (-3,0) | -0.01 (-0.02,0) |
| Italy | -24 (-54,4) | -0.02 (-0.06,0) | -29 (-70,5) | -0.02 (-0.04,0) |
| Jamaica | -4 (-8,1) | -0.2 (-0.43,0.04) | -10 (-21,2) | -0.3 (-0.65,0.05) |
| Japan | -47 (-103,9) | -0.03 (-0.06,0.01) | -151 (-332,26) | -0.03 (-0.06,0.01) |
| Jordan | -1 (-1,0) | -0.08 (-0.18,0.01) | -3 (-7,0) | -0.06 (-0.14,0.01) |
| Kazakhstan | -1 (-3,0) | -0.01 (-0.03,0) | -1 (-2,0) | -0.01 (-0.01,0) |
| Kenya | -6 (-15,1) | -0.1 (-0.23,0.01) | -15 (-33,3) | -0.09 (-0.2,0.02) |
| Kingdom of Eswatini | -1 (-2,0) | -0.4 (-0.9,0.07) | -2 (-4,0) | -0.37 (-0.82,0.07) |
| Kiribati | 0 (0,0) | -0.2 (-0.48,0.03) | 0 (0,0) | -0.2 (-0.49,0.03) |
| Kuwait | 0 (0,0) | -0.02 (-0.04,0) | 0 (-1,0) | -0.02 (-0.05,0) |
| Kyrgyzstan | 0 (-1,0) | -0.01 (-0.03,0) | 0 (-1,0) | -0.01 (-0.02,0) |
| Lao People's Democratic Republic | -3 (-6,1) | -0.18 (-0.41,0.04) | -7 (-14,1) | -0.2 (-0.44,0.04) |
| Latvia | 0 (-1,0) | -0.01 (-0.03,0) | -1 (-2,0) | -0.02 (-0.05,0) |
| Lebanon | -1 (-2,0) | -0.05 (-0.11,0.01) | -4 (-8,1) | -0.05 (-0.12,0.01) |
| Lesotho | -3 (-7,0) | -0.42 (-0.97,0.06) | -5 (-11,1) | -0.51 (-1.14,0.09) |
| Liberia | -3 (-8,1) | -0.35 (-0.82,0.06) | -8 (-19,1) | -0.55 (-1.35,0.1) |
| Libya | -1 (-3,0) | -0.07 (-0.19,0.01) | -4 (-8,1) | -0.1 (-0.22,0.02) |
| Lithuania | -1 (-3,0) | -0.03 (-0.06,0) | -1 (-3,0) | -0.02 (-0.05,0) |
| Luxembourg | 0 (0,0) | -0.02 (-0.05,0) | 0 (0,0) | -0.01 (-0.02,0) |
| Madagascar | -12 (-31,2) | -0.31 (-0.78,0.06) | -20 (-49,4) | -0.28 (-0.67,0.05) |
| Malawi | -9 (-21,1) | -0.32 (-0.75,0.05) | -20 (-46,3) | -0.35 (-0.78,0.06) |
| Malaysia | -10 (-23,2) | -0.13 (-0.3,0.02) | -31 (-67,6) | -0.13 (-0.29,0.03) |
| Maldives | 0 (0,0) | -0.16 (-0.38,0.03) | 0 (0,0) | -0.06 (-0.15,0.01) |
| Mali | -4 (-9,1) | -0.15 (-0.34,0.03) | -6 (-14,1) | -0.11 (-0.25,0.02) |
| Malta | 0 (0,0) | -0.03 (-0.07,0.01) | 0 (0,0) | -0.01 (-0.03,0) |
| Marshall Islands | 0 (0,0) | -0.33 (-0.77,0.06) | 0 (0,0) | -0.49 (-1.13,0.08) |
| Mauritania | -1 (-1,0) | -0.07 (-0.17,0.01) | -2 (-5,0) | -0.12 (-0.28,0.02) |
| Mauritius | 0 (-1,0) | -0.08 (-0.16,0.01) | -1 (-2,0) | -0.07 (-0.14,0.01) |
| Mexico | -51 (-110,9) | -0.16 (-0.34,0.03) | -114 (-249,22) | -0.1 (-0.22,0.02) |
| Mongolia | 0 (-1,0) | -0.02 (-0.06,0) | 0 (-1,0) | -0.02 (-0.04,0) |
| Montenegro | 0 (0,0) | -0.01 (-0.03,0) | 0 (0,0) | -0.02 (-0.04,0) |
| Morocco | -8 (-18,2) | -0.07 (-0.15,0.01) | -14 (-31,3) | -0.05 (-0.11,0.01) |
| Mozambique | -8 (-18,1) | -0.18 (-0.43,0.03) | -13 (-30,2) | -0.17 (-0.38,0.03) |
| Myanmar | -30 (-65,6) | -0.17 (-0.38,0.03) | -56 (-122,12) | -0.14 (-0.31,0.03) |
| Namibia | -2 (-4,0) | -0.3 (-0.75,0.05) | -4 (-10,1) | -0.39 (-0.87,0.08) |
| Nepal | -6 (-13,1) | -0.09 (-0.21,0.01) | -12 (-32,2) | -0.06 (-0.18,0.01) |
| Netherlands | -3 (-8,1) | -0.02 (-0.04,0) | -5 (-11,1) | -0.01 (-0.03,0) |
| New Zealand | -3 (-6,1) | -0.07 (-0.16,0.01) | -8 (-18,2) | -0.09 (-0.2,0.02) |
| Nicaragua | -2 (-6,0) | -0.19 (-0.45,0.03) | -6 (-13,1) | -0.14 (-0.3,0.03) |
| Niger | -4 (-9,1) | -0.2 (-0.47,0.03) | -13 (-33,2) | -0.25 (-0.64,0.04) |
| Nigeria | -233 (-552,42) | -0.67 (-1.58,0.12) | -420 (-1007,90) | -0.68 (-1.6,0.14) |
| Northern Mariana Islands | 0 (0,0) | -0.16 (-0.4,0.03) | 0 (0,0) | -0.21 (-0.48,0.04) |
| Norway | -2 (-5,0) | -0.03 (-0.07,0) | -2 (-6,0) | -0.02 (-0.05,0) |
| Oman | 0 (0,0) | -0.03 (-0.07,0.01) | 0 (0,0) | -0.01 (-0.03,0) |
| Pakistan | -56 (-139,9) | -0.12 (-0.31,0.02) | -87 (-205,14) | -0.1 (-0.25,0.02) |
| Palestine | -2 (-4,0) | -0.29 (-0.66,0.05) | -4 (-10,1) | -0.25 (-0.57,0.05) |
| Panama | -3 (-7,1) | -0.25 (-0.54,0.05) | -7 (-15,1) | -0.16 (-0.33,0.03) |
| Papua New Guinea | -3 (-7,0) | -0.29 (-0.74,0.04) | -12 (-28,1) | -0.4 (-0.99,0.04) |
| Paraguay | -2 (-6,0) | -0.13 (-0.3,0.02) | -8 (-17,1) | -0.15 (-0.34,0.03) |
| Peru | -25 (-55,4) | -0.24 (-0.54,0.04) | -46 (-101,9) | -0.14 (-0.31,0.03) |
| Philippines | -62 (-145,9) | -0.32 (-0.76,0.05) | -164 (-371,28) | -0.25 (-0.57,0.04) |
| Plurinational State of Bolivia | -12 (-26,2) | -0.48 (-1.05,0.09) | -32 (-74,6) | -0.44 (-1.01,0.08) |
| Poland | -9 (-20,2) | -0.02 (-0.05,0) | -23 (-54,4) | -0.03 (-0.07,0.01) |
| Portugal | -10 (-23,2) | -0.07 (-0.17,0.01) | -9 (-21,2) | -0.03 (-0.06,0) |
| Principality of Monaco | 0 (0,0) | -0.02 (-0.04,0) | 0 (0,0) | -0.02 (-0.05,0) |
| Puerto Rico | -6 (-13,1) | -0.18 (-0.37,0.03) | -6 (-13,1) | -0.07 (-0.15,0.01) |
| Qatar | 0 (0,0) | -0.06 (-0.15,0.01) | 0 (0,0) | -0.03 (-0.07,0) |
| Republic of Cabo Verde | -1 (-2,0) | -0.22 (-0.63,0.04) | -1 (-2,0) | -0.25 (-0.6,0.04) |
| Republic of Côte d'Ivoire | -18 (-40,3) | -0.71 (-1.6,0.12) | -51 (-118,9) | -0.67 (-1.52,0.12) |
| Republic of Korea | -6 (-17,1) | -0.03 (-0.08,0.01) | -28 (-66,5) | -0.03 (-0.07,0) |
| Republic of Moldova | -1 (-2,0) | -0.03 (-0.06,0.01) | -2 (-5,0) | -0.03 (-0.08,0.01) |
| Republic of Nauru | 0 (0,0) | -0.38 (-0.91,0.07) | 0 (0,0) | -0.33 (-0.77,0.06) |
| Republic of Niue | 0 (0,0) | -0.26 (-0.61,0.04) | 0 (0,0) | -0.28 (-0.63,0.05) |
| Republic of Palau | 0 (0,0) | -0.41 (-0.99,0.07) | 0 (0,0) | -0.34 (-0.83,0.06) |
| Republic of San Marino | 0 (0,0) | -0.02 (-0.04,0) | 0 (0,0) | -0.01 (-0.02,0) |
| Republic of the Gambia | 0 (-1,0) | -0.09 (-0.21,0.02) | -1 (-2,0) | -0.1 (-0.23,0.02) |
| Romania | -4 (-10,1) | -0.02 (-0.04,0) | -4 (-9,1) | -0.01 (-0.02,0) |
| Russian Federation | -33 (-71,6) | -0.02 (-0.04,0) | -58 (-131,10) | -0.02 (-0.05,0) |
| Rwanda | -7 (-16,1) | -0.33 (-0.77,0.07) | -11 (-25,2) | -0.23 (-0.55,0.04) |
| Saint Kitts and Nevis | 0 (0,0) | -0.52 (-1.09,0.09) | 0 (-1,0) | -0.57 (-1.22,0.1) |
| Saint Lucia | 0 (-1,0) | -0.5 (-1.08,0.1) | -1 (-2,0) | -0.44 (-0.96,0.07) |
| Saint Vincent and the Grenadines | 0 (-1,0) | -0.58 (-1.22,0.11) | -1 (-2,0) | -0.58 (-1.24,0.11) |
| Samoa | 0 (0,0) | -0.18 (-0.41,0.03) | 0 (0,0) | -0.16 (-0.35,0.03) |
| Sao Tome and Principe | 0 (0,0) | -0.24 (-0.54,0.05) | 0 (-1,0) | -0.32 (-0.68,0.06) |
| Saudi Arabia | -2 (-4,0) | -0.04 (-0.11,0.01) | -2 (-7,0) | -0.03 (-0.08,0) |
| Senegal | -8 (-20,2) | -0.33 (-0.78,0.06) | -27 (-62,6) | -0.46 (-1.05,0.09) |
| Serbia | -5 (-11,1) | -0.06 (-0.14,0.01) | -8 (-20,2) | -0.05 (-0.11,0.01) |
| Seychelles | 0 (-1,0) | -0.34 (-0.99,0.06) | 0 (-1,0) | -0.37 (-0.81,0.07) |
| Sierra Leone | -6 (-15,1) | -0.36 (-0.88,0.06) | -14 (-34,3) | -0.51 (-1.2,0.1) |
| Singapore | -1 (-1,0) | -0.04 (-0.09,0.01) | -2 (-3,0) | -0.02 (-0.04,0) |
| Slovakia | -4 (-11,1) | -0.07 (-0.18,0.01) | -7 (-15,1) | -0.07 (-0.16,0.01) |
| Slovenia | -1 (-2,0) | -0.03 (-0.06,0) | -1 (-3,0) | -0.02 (-0.05,0) |
| Socialist Republic of Viet Nam | -16 (-37,3) | -0.04 (-0.1,0.01) | -39 (-94,8) | -0.05 (-0.11,0.01) |
| Solomon Islands | 0 (-1,0) | -0.35 (-0.86,0.05) | -1 (-2,0) | -0.34 (-0.77,0.05) |
| Somalia | -6 (-15,1) | -0.37 (-0.98,0.06) | -12 (-31,2) | -0.29 (-0.72,0.04) |
| South Africa | -68 (-162,10) | -0.39 (-0.95,0.06) | -168 (-371,32) | -0.44 (-0.96,0.08) |
| South Sudan | -7 (-17,1) | -0.31 (-0.77,0.06) | -7 (-18,1) | -0.28 (-0.69,0.05) |
| Spain | -27 (-62,5) | -0.05 (-0.11,0.01) | -39 (-90,6) | -0.03 (-0.07,0.01) |
| Sri Lanka | -9 (-21,2) | -0.12 (-0.27,0.02) | -17 (-38,3) | -0.07 (-0.16,0.01) |
| Sudan | -7 (-16,1) | -0.1 (-0.24,0.02) | -16 (-37,3) | -0.11 (-0.26,0.02) |
| Suriname | -1 (-2,0) | -0.29 (-0.72,0.05) | -2 (-4,0) | -0.31 (-0.74,0.05) |
| Sweden | -4 (-9,1) | -0.02 (-0.05,0) | -4 (-9,1) | -0.01 (-0.03,0) |
| Switzerland | -3 (-6,1) | -0.02 (-0.05,0) | -3 (-7,0) | -0.01 (-0.03,0) |
| Syrian Arab Republic | -3 (-7,0) | -0.07 (-0.19,0.01) | -7 (-17,1) | -0.09 (-0.2,0.02) |
| Taiwan (Province of China) | -4 (-8,1) | -0.03 (-0.07,0.01) | -18 (-40,3) | -0.04 (-0.09,0.01) |
| Tajikistan | -1 (-3,0) | -0.06 (-0.13,0.01) | -2 (-5,0) | -0.05 (-0.1,0.01) |
| Thailand | -47 (-99,10) | -0.17 (-0.37,0.03) | -142 (-328,26) | -0.13 (-0.3,0.02) |
| The former Yugoslav Republic of Macedonia | -1 (-2,0) | -0.06 (-0.16,0.01) | -1 (-2,0) | -0.04 (-0.09,0.01) |
| Timor-Leste | 0 (-1,0) | -0.16 (-0.39,0.02) | -1 (-3,0) | -0.2 (-0.46,0.04) |
| Togo | -3 (-7,1) | -0.35 (-0.85,0.06) | -12 (-26,2) | -0.45 (-1.03,0.09) |
| Tokelau | 0 (0,0) | -0.42 (-1.01,0.06) | 0 (0,0) | -0.37 (-0.84,0.06) |
| Tonga | 0 (-1,0) | -0.52 (-1.19,0.08) | 0 (-1,0) | -0.48 (-1.1,0.09) |
| Trinidad and Tobago | -2 (-5,0) | -0.33 (-0.71,0.06) | -4 (-9,1) | -0.23 (-0.5,0.04) |
| Tunisia | -2 (-4,0) | -0.05 (-0.12,0.01) | -3 (-7,1) | -0.03 (-0.06,0.01) |
| Turkey | -11 (-26,2) | -0.04 (-0.1,0.01) | -21 (-51,4) | -0.03 (-0.06,0) |
| Turkmenistan | 0 (-1,0) | -0.02 (-0.04,0) | 0 (-1,0) | -0.01 (-0.02,0) |
| Tuvalu | 0 (0,0) | -0.3 (-0.73,0.05) | 0 (0,0) | -0.37 (-0.87,0.07) |
| Uganda | -27 (-63,5) | -0.55 (-1.28,0.1) | -57 (-131,10) | -0.51 (-1.19,0.09) |
| Ukraine | -15 (-33,3) | -0.02 (-0.05,0) | -21 (-50,4) | -0.03 (-0.06,0) |
| United Arab Emirates | 0 (0,0) | -0.02 (-0.05,0) | -1 (-1,0) | -0.07 (-0.17,0.01) |
| United Kingdom of Great Britain and Northern Ireland | -35 (-81,6) | -0.03 (-0.08,0.01) | -33 (-79,6) | -0.02 (-0.05,0) |
| United Republic of Tanzania | -36 (-90,6) | -0.42 (-1.03,0.08) | -65 (-159,11) | -0.33 (-0.79,0.06) |
| United States of America | -100 (-233,16) | -0.03 (-0.07,0) | -86 (-198,14) | -0.01 (-0.03,0) |
| United States Virgin Islands | 0 (0,0) | -0.26 (-0.6,0.04) | 0 (0,0) | -0.11 (-0.25,0.02) |
| Uruguay | -5 (-10,1) | -0.12 (-0.26,0.02) | -3 (-7,1) | -0.05 (-0.11,0.01) |
| Uzbekistan | -2 (-3,0) | -0.01 (-0.03,0) | -2 (-5,0) | -0.01 (-0.03,0) |
| Vanuatu | 0 (0,0) | -0.42 (-0.98,0.06) | 0 (-1,0) | -0.42 (-0.96,0.07) |
| Yemen | -3 (-8,0) | -0.1 (-0.23,0.01) | -15 (-37,2) | -0.16 (-0.38,0.02) |
| Zambia | -12 (-27,3) | -0.55 (-1.32,0.12) | -44 (-112,6) | -0.83 (-2.08,0.12) |
| Zimbabwe | -24 (-56,4) | -0.78 (-1.78,0.14) | -47 (-105,8) | -0.98 (-2.19,0.17) |
| ASMR, age-standardized mortality rate; UI, uncertainty interval. | | | | |

| Table S4. The DALYs cases, age-standardized DALYs, and temporal trends of prostate cancer attributable to diet low in calcium in 204 countries, 1990 and 2021 | | | | |
| --- | --- | --- | --- | --- |
|  | 1990 | 1990 | 2021 | 2021 |
| Location | DALYs  No. (95% UI) | ASDR per 100,000  No. (95% UI) | DALYs  No. (95% UI) | ASDR per 100,000  No. (95% UI) |
| Afghanistan | -107 (-245,18) | -1.86 (-4.38,0.3) | -136 (-317,23) | -1.93 (-4.41,0.31) |
| Albania | -8 (-21,1) | -0.47 (-1.19,0.08) | -7 (-18,1) | -0.16 (-0.4,0.03) |
| Algeria | -36 (-80,7) | -0.43 (-1.02,0.09) | -54 (-131,9) | -0.2 (-0.49,0.04) |
| American Samoa | -1 (-3,0) | -7.67 (-17.98,1.33) | -3 (-8,1) | -8.62 (-19.51,1.5) |
| Andorra | 0 (0,0) | -0.33 (-0.84,0.05) | 0 (-1,0) | -0.25 (-0.65,0.04) |
| Angola | -202 (-463,42) | -7.03 (-16.07,1.45) | -536 (-1242,103) | -6.15 (-14.14,1.17) |
| Antigua and Barbuda | -3 (-7,1) | -5.36 (-11.59,0.99) | -5 (-12,1) | -5.65 (-12.08,0.98) |
| Argentina | -517 (-1131,97) | -1.64 (-3.59,0.31) | -586 (-1348,117) | -1 (-2.31,0.2) |
| Armenia | -25 (-54,5) | -0.95 (-2.07,0.17) | -18 (-43,3) | -0.42 (-0.97,0.08) |
| Australia | -182 (-423,34) | -0.91 (-2.11,0.17) | -205 (-494,32) | -0.41 (-0.98,0.06) |
| Austria | -60 (-141,11) | -0.46 (-1.07,0.08) | -55 (-128,11) | -0.27 (-0.63,0.05) |
| Azerbaijan | -59 (-133,11) | -1.24 (-2.78,0.22) | -50 (-117,9) | -0.55 (-1.29,0.1) |
| Bahrain | -2 (-4,0) | -1.47 (-3.54,0.26) | -6 (-15,1) | -1.07 (-2.66,0.2) |
| Bangladesh | -1126 (-2609,150) | -2.7 (-6.26,0.36) | -2561 (-7249,337) | -2.02 (-5.75,0.27) |
| Barbados | -12 (-24,2) | -3.49 (-7.39,0.61) | -20 (-44,4) | -3.71 (-8.26,0.68) |
| Belarus | -25 (-59,5) | -0.19 (-0.45,0.03) | -54 (-133,10) | -0.33 (-0.8,0.06) |
| Belgium | -88 (-207,16) | -0.53 (-1.24,0.09) | -64 (-153,12) | -0.24 (-0.58,0.04) |
| Belize | -2 (-4,0) | -2.15 (-4.51,0.42) | -9 (-19,2) | -3.36 (-7.37,0.59) |
| Benin | -99 (-227,17) | -5.5 (-12.73,0.97) | -272 (-626,51) | -6.31 (-14.51,1.2) |
| Bermuda | -1 (-3,0) | -2.23 (-5.11,0.4) | -4 (-9,1) | -2.77 (-6.3,0.46) |
| Bhutan | -3 (-7,0) | -1.43 (-3.55,0.18) | -6 (-17,1) | -1.08 (-3.07,0.16) |
| Bolivarian Republic of Venezuela | -330 (-690,62) | -3.82 (-8,0.72) | -1369 (-2997,222) | -4.83 (-10.49,0.79) |
| Bosnia and Herzegovina | -35 (-82,6) | -0.97 (-2.31,0.18) | -25 (-62,4) | -0.38 (-0.94,0.06) |
| Botswana | -24 (-54,5) | -5.12 (-11.79,1.03) | -51 (-117,10) | -4.15 (-9.49,0.77) |
| Brazil | -2090 (-4433,399) | -2.7 (-5.69,0.52) | -3026 (-6534,551) | -1.25 (-2.69,0.23) |
| Brunei Darussalam | -1 (-3,0) | -1.29 (-2.99,0.23) | -2 (-5,0) | -0.87 (-1.98,0.17) |
| Bulgaria | -58 (-130,10) | -0.5 (-1.13,0.09) | -133 (-290,21) | -0.84 (-1.85,0.14) |
| Burkina Faso | -178 (-430,28) | -4.82 (-11.42,0.77) | -480 (-1151,92) | -6.13 (-14.76,1.16) |
| Burundi | -113 (-268,23) | -5.4 (-12.95,1.08) | -270 (-673,44) | -6.68 (-16.32,1.1) |
| Cambodia | -131 (-290,25) | -3.48 (-7.78,0.66) | -411 (-924,72) | -3.9 (-8.8,0.7) |
| Cameroon | -211 (-490,39) | -5.83 (-13.74,1.08) | -723 (-1668,140) | -7.17 (-16.23,1.4) |
| Canada | -398 (-894,75) | -1.2 (-2.68,0.22) | -322 (-738,60) | -0.41 (-0.94,0.08) |
| Central African Republic | -46 (-103,9) | -5.34 (-12.09,1.08) | -84 (-195,18) | -5.16 (-12.09,1.07) |
| Chad | -100 (-236,16) | -3.86 (-9.14,0.62) | -327 (-775,59) | -6.98 (-16.46,1.26) |
| Chile | -328 (-692,57) | -3.5 (-7.39,0.61) | -638 (-1421,122) | -2.42 (-5.4,0.46) |
| China | -5357 (-11938,871) | -0.78 (-1.71,0.13) | -8709 (-20672,1608) | -0.44 (-1.03,0.08) |
| Colombia | -534 (-1128,104) | -3.49 (-7.34,0.68) | -1042 (-2317,165) | -1.91 (-4.25,0.3) |
| Commonwealth of the Bahamas | -6 (-14,1) | -4.37 (-9.57,0.79) | -16 (-35,3) | -4.6 (-10.02,0.79) |
| Comoros | -14 (-35,2) | -8 (-19.79,1.32) | -36 (-88,6) | -8.35 (-20.2,1.4) |
| Congo | -71 (-161,13) | -8.35 (-18.03,1.6) | -177 (-393,33) | -8.86 (-19.26,1.68) |
| Cook Islands | -1 (-3,0) | -12.96 (-29.64,2.24) | -2 (-4,0) | -7.45 (-16.62,1.33) |
| Costa Rica | -27 (-58,5) | -1.67 (-3.59,0.32) | -65 (-144,11) | -1.19 (-2.66,0.21) |
| Croatia | -59 (-132,12) | -1.08 (-2.4,0.21) | -54 (-131,10) | -0.54 (-1.29,0.1) |
| Cuba | -315 (-667,58) | -3.12 (-6.62,0.58) | -546 (-1160,103) | -2.65 (-5.64,0.5) |
| Cyprus | -11 (-27,2) | -1.74 (-4.36,0.28) | -22 (-53,3) | -1 (-2.4,0.16) |
| Czech Republic | -72 (-168,13) | -0.49 (-1.15,0.09) | -91 (-215,16) | -0.38 (-0.91,0.07) |
| Democratic People's Republic of Korea | -111 (-242,22) | -0.81 (-1.78,0.16) | -349 (-778,65) | -1.09 (-2.43,0.2) |
| Democratic Republic of the Congo | -731 (-1752,139) | -6.54 (-15.31,1.25) | -2132 (-5012,369) | -7.43 (-17.2,1.26) |
| Denmark | -59 (-137,10) | -0.66 (-1.54,0.11) | -46 (-105,9) | -0.34 (-0.78,0.07) |
| Djibouti | -7 (-18,1) | -6.74 (-16.64,1.22) | -32 (-77,6) | -6.3 (-14.84,1.15) |
| Dominica | -1 (-3,0) | -2.26 (-5.29,0.39) | -1 (-3,0) | -1.42 (-3.61,0.25) |
| Dominican Republic | -187 (-509,31) | -6 (-16.33,0.98) | -445 (-1144,76) | -4.63 (-11.93,0.79) |
| Ecuador | -155 (-327,29) | -3.35 (-7.07,0.63) | -435 (-931,77) | -2.78 (-5.96,0.49) |
| Egypt | -206 (-539,36) | -1.01 (-2.91,0.17) | -461 (-1068,78) | -1.09 (-2.49,0.17) |
| El Salvador | -92 (-253,15) | -3.29 (-9.14,0.54) | -149 (-347,25) | -2.32 (-5.4,0.39) |
| Equatorial Guinea | -11 (-24,2) | -7.01 (-15.75,1.35) | -17 (-39,4) | -4.59 (-10.26,0.97) |
| Eritrea | -56 (-137,13) | -6.05 (-14.52,1.41) | -135 (-332,31) | -5.94 (-14.52,1.39) |
| Estonia | -5 (-11,1) | -0.22 (-0.53,0.04) | -10 (-23,2) | -0.33 (-0.78,0.06) |
| Ethiopia | -576 (-1418,91) | -3.38 (-8.34,0.52) | -963 (-2392,118) | -2.64 (-6.64,0.33) |
| Federated States of Micronesia | -3 (-7,0) | -6.49 (-16.32,1.1) | -4 (-9,1) | -6.82 (-15.49,1.15) |
| Fiji | -10 (-27,2) | -3.93 (-10.31,0.84) | -21 (-53,5) | -3.29 (-8.49,0.74) |
| Finland | -21 (-49,4) | -0.28 (-0.66,0.05) | -30 (-69,6) | -0.21 (-0.48,0.04) |
| France | -368 (-850,65) | -0.4 (-0.93,0.07) | -441 (-1001,80) | -0.27 (-0.62,0.05) |
| Gabon | -36 (-79,7) | -6.92 (-15.17,1.31) | -57 (-132,11) | -6.61 (-15.22,1.36) |
| Georgia | -38 (-86,7) | -0.61 (-1.38,0.11) | -64 (-146,12) | -1.05 (-2.38,0.2) |
| Germany | -680 (-1552,119) | -0.49 (-1.11,0.09) | -590 (-1349,109) | -0.26 (-0.6,0.05) |
| Ghana | -508 (-1168,81) | -10.11 (-23.06,1.64) | -802 (-1835,148) | -5.82 (-13.08,1.09) |
| Greece | -57 (-135,10) | -0.36 (-0.85,0.06) | -82 (-192,14) | -0.27 (-0.63,0.05) |
| Greenland | 0 (0,0) | -0.3 (-0.73,0.05) | 0 (0,0) | -0.17 (-0.43,0.03) |
| Grenada | -4 (-9,1) | -5.4 (-11.21,0.98) | -7 (-15,1) | -5.92 (-13.04,1.18) |
| Guam | -1 (-3,0) | -1.98 (-4.65,0.36) | -2 (-6,0) | -1.14 (-2.65,0.2) |
| Guatemala | -101 (-211,20) | -3.8 (-7.91,0.74) | -437 (-932,79) | -4.31 (-9.25,0.78) |
| Guinea | -144 (-335,24) | -4.83 (-11.18,0.79) | -272 (-607,53) | -5.61 (-12.46,1.08) |
| Guinea-Bissau | -25 (-59,5) | -7.57 (-17.59,1.46) | -57 (-131,11) | -10.15 (-23.18,2.03) |
| Guyana | -32 (-67,6) | -9.64 (-20.17,1.77) | -31 (-67,6) | -5.62 (-12.11,1.02) |
| Haiti | -377 (-879,70) | -14.17 (-33.38,2.59) | -854 (-1948,165) | -15.48 (-35.48,3.06) |
| Honduras | -53 (-122,10) | -3 (-6.8,0.57) | -138 (-342,28) | -2.58 (-6.35,0.53) |
| Hungary | -134 (-301,25) | -0.89 (-2,0.17) | -166 (-371,31) | -0.78 (-1.75,0.14) |
| Iceland | -1 (-2,0) | -0.35 (-0.82,0.07) | -2 (-4,0) | -0.25 (-0.59,0.04) |
| India | -3527 (-8183,729) | -0.96 (-2.24,0.2) | -8833 (-19586,1535) | -0.84 (-1.88,0.15) |
| Indonesia | -2361 (-5271,423) | -2.93 (-6.63,0.53) | -7366 (-16585,1285) | -3.75 (-8.5,0.65) |
| Iraq | -79 (-200,11) | -1.11 (-2.82,0.15) | -316 (-749,55) | -1.66 (-3.92,0.29) |
| Ireland | -17 (-40,3) | -0.39 (-0.91,0.07) | -16 (-38,3) | -0.19 (-0.45,0.03) |
| Islamic Republic of Iran | -376 (-849,71) | -1.83 (-4.18,0.34) | -1121 (-2466,213) | -1.66 (-3.64,0.32) |
| Israel | -20 (-46,4) | -0.4 (-0.92,0.07) | -20 (-47,3) | -0.15 (-0.35,0.02) |
| Italy | -418 (-963,75) | -0.43 (-1,0.08) | -448 (-1043,77) | -0.26 (-0.6,0.05) |
| Jamaica | -65 (-142,12) | -3.48 (-7.58,0.65) | -159 (-354,28) | -5.14 (-11.57,0.9) |
| Japan | -842 (-1838,156) | -0.5 (-1.09,0.09) | -2269 (-4994,403) | -0.5 (-1.11,0.09) |
| Jordan | -13 (-29,2) | -1.33 (-2.87,0.23) | -59 (-137,10) | -1.04 (-2.37,0.17) |
| Kazakhstan | -30 (-70,5) | -0.25 (-0.58,0.04) | -19 (-45,4) | -0.11 (-0.27,0.02) |
| Kenya | -121 (-293,16) | -1.7 (-4.14,0.23) | -316 (-713,58) | -1.63 (-3.65,0.3) |
| Kingdom of Eswatini | -19 (-42,3) | -7.58 (-16.99,1.41) | -37 (-86,7) | -7.33 (-16.7,1.33) |
| Kiribati | -1 (-2,0) | -3.55 (-8.41,0.6) | -2 (-5,0) | -3.42 (-8.17,0.58) |
| Kuwait | -1 (-3,0) | -0.29 (-0.67,0.05) | -8 (-18,1) | -0.37 (-0.9,0.06) |
| Kyrgyzstan | -7 (-15,1) | -0.23 (-0.51,0.04) | -7 (-15,1) | -0.16 (-0.37,0.03) |
| Lao People's Democratic Republic | -60 (-136,12) | -3.32 (-7.58,0.66) | -129 (-284,24) | -3.39 (-7.46,0.65) |
| Latvia | -8 (-18,1) | -0.21 (-0.49,0.04) | -16 (-38,2) | -0.36 (-0.88,0.06) |
| Lebanon | -13 (-30,2) | -0.76 (-1.66,0.14) | -53 (-126,10) | -0.8 (-1.92,0.16) |
| Lesotho | -64 (-150,10) | -8.02 (-18.66,1.23) | -109 (-249,21) | -10.55 (-23.71,2) |
| Liberia | -60 (-143,10) | -5.91 (-13.91,1.02) | -153 (-377,27) | -9.36 (-22.84,1.64) |
| Libya | -19 (-46,3) | -1.18 (-2.94,0.18) | -65 (-146,14) | -1.59 (-3.56,0.34) |
| Lithuania | -21 (-49,4) | -0.46 (-1.07,0.08) | -25 (-62,4) | -0.41 (-1.01,0.06) |
| Luxembourg | -2 (-4,0) | -0.33 (-0.76,0.06) | -2 (-5,0) | -0.17 (-0.4,0.03) |
| Madagascar | -257 (-667,52) | -5.79 (-14.6,1.16) | -461 (-1101,79) | -5.16 (-12.3,0.92) |
| Malawi | -196 (-456,31) | -5.92 (-13.74,0.95) | -445 (-1026,77) | -6.78 (-15.45,1.18) |
| Malaysia | -189 (-428,36) | -2.36 (-5.31,0.44) | -586 (-1262,117) | -2.3 (-4.96,0.47) |
| Maldives | -2 (-3,0) | -2.51 (-5.91,0.5) | -3 (-6,1) | -0.98 (-2.26,0.21) |
| Mali | -81 (-178,15) | -2.55 (-5.63,0.46) | -120 (-274,23) | -1.76 (-3.93,0.35) |
| Malta | -2 (-5,0) | -0.53 (-1.18,0.11) | -2 (-6,0) | -0.21 (-0.51,0.04) |
| Marshall Islands | -1 (-2,0) | -5.77 (-13.39,1.07) | -2 (-4,0) | -7.79 (-17.98,1.31) |
| Mauritania | -10 (-23,2) | -1.16 (-2.67,0.2) | -34 (-81,7) | -1.89 (-4.45,0.37) |
| Mauritius | -9 (-18,2) | -1.35 (-2.85,0.25) | -20 (-42,4) | -1.12 (-2.38,0.21) |
| Mexico | -918 (-1961,172) | -2.54 (-5.45,0.48) | -2032 (-4448,393) | -1.73 (-3.78,0.33) |
| Mongolia | -4 (-12,1) | -0.41 (-1.14,0.07) | -6 (-13,1) | -0.28 (-0.65,0.05) |
| Montenegro | -1 (-3,0) | -0.22 (-0.57,0.04) | -3 (-7,0) | -0.29 (-0.72,0.04) |
| Morocco | -140 (-319,29) | -1.11 (-2.51,0.23) | -262 (-578,54) | -0.86 (-1.88,0.18) |
| Mozambique | -152 (-359,26) | -3.15 (-7.39,0.54) | -280 (-666,50) | -3.08 (-7.04,0.55) |
| Myanmar | -623 (-1372,118) | -3.12 (-6.87,0.59) | -1068 (-2344,239) | -2.49 (-5.46,0.56) |
| Namibia | -35 (-83,6) | -5.88 (-14.15,0.99) | -94 (-213,18) | -7.6 (-16.85,1.49) |
| Nepal | -112 (-271,16) | -1.5 (-3.62,0.21) | -219 (-600,35) | -1.07 (-2.92,0.17) |
| Netherlands | -58 (-138,11) | -0.28 (-0.65,0.05) | -83 (-193,16) | -0.22 (-0.5,0.04) |
| New Zealand | -50 (-116,10) | -1.23 (-2.86,0.24) | -144 (-317,26) | -1.58 (-3.49,0.29) |
| Nicaragua | -45 (-106,8) | -3.33 (-7.86,0.56) | -112 (-246,21) | -2.48 (-5.42,0.48) |
| Niger | -79 (-187,13) | -3.61 (-8.46,0.6) | -251 (-643,42) | -4.04 (-10.33,0.67) |
| Nigeria | -4607 (-11079,842) | -11.81 (-28.23,2.16) | -7727 (-18668,1679) | -10.88 (-26,2.35) |
| Northern Mariana Islands | 0 (-1,0) | -2.58 (-6.28,0.41) | -1 (-3,0) | -3.46 (-7.72,0.63) |
| Norway | -36 (-85,6) | -0.47 (-1.12,0.08) | -35 (-83,6) | -0.32 (-0.75,0.05) |
| Oman | -3 (-7,1) | -0.57 (-1.33,0.1) | -3 (-7,1) | -0.23 (-0.53,0.04) |
| Pakistan | -1036 (-2595,174) | -2.09 (-5.21,0.35) | -1642 (-3816,258) | -1.71 (-4.01,0.27) |
| Palestine | -34 (-79,6) | -4.62 (-10.64,0.8) | -87 (-208,17) | -4.31 (-10.12,0.83) |
| Panama | -60 (-130,11) | -4.39 (-9.44,0.81) | -122 (-257,23) | -2.74 (-5.8,0.51) |
| Papua New Guinea | -62 (-155,8) | -4.87 (-12.14,0.65) | -239 (-586,27) | -6.63 (-15.99,0.73) |
| Paraguay | -43 (-101,7) | -2.11 (-4.95,0.36) | -132 (-308,26) | -2.46 (-5.7,0.48) |
| Peru | -419 (-943,72) | -3.91 (-8.81,0.68) | -741 (-1670,143) | -2.28 (-5.15,0.44) |
| Philippines | -1215 (-2787,183) | -5.21 (-12.06,0.79) | -3378 (-7644,561) | -4.65 (-10.47,0.78) |
| Plurinational State of Bolivia | -221 (-492,43) | -8.06 (-17.89,1.55) | -585 (-1341,109) | -7.25 (-16.76,1.35) |
| Poland | -161 (-358,29) | -0.36 (-0.81,0.07) | -393 (-918,67) | -0.51 (-1.19,0.09) |
| Portugal | -175 (-399,32) | -1.23 (-2.81,0.22) | -128 (-301,23) | -0.44 (-1.03,0.08) |
| Principality of Monaco | 0 (-1,0) | -0.29 (-0.7,0.05) | 0 (-1,0) | -0.4 (-0.96,0.06) |
| Puerto Rico | -100 (-217,18) | -2.79 (-6.04,0.5) | -98 (-214,16) | -1.2 (-2.62,0.2) |
| Qatar | -1 (-1,0) | -0.95 (-2.46,0.14) | -2 (-6,0) | -0.45 (-1.18,0.08) |
| Republic of Cabo Verde | -9 (-25,1) | -3.64 (-10.42,0.58) | -17 (-40,3) | -4.15 (-9.88,0.72) |
| Republic of Côte d'Ivoire | -377 (-859,64) | -12.63 (-28.71,2.14) | -1047 (-2430,186) | -11.91 (-27.53,2.14) |
| Republic of Korea | -128 (-337,22) | -0.52 (-1.41,0.09) | -467 (-1093,77) | -0.49 (-1.15,0.08) |
| Republic of Moldova | -22 (-47,4) | -0.52 (-1.12,0.1) | -46 (-103,9) | -0.74 (-1.68,0.14) |
| Republic of Nauru | 0 (0,0) | -5.92 (-13.76,1.11) | 0 (-1,0) | -5.4 (-12.42,0.98) |
| Republic of Niue | 0 (0,0) | -4.46 (-10.55,0.74) | 0 (0,0) | -4.71 (-10.44,0.85) |
| Republic of Palau | -1 (-1,0) | -6.79 (-16.46,1.16) | -1 (-2,0) | -5.56 (-13.31,0.95) |
| Republic of San Marino | 0 (0,0) | -0.29 (-0.72,0.05) | 0 (0,0) | -0.15 (-0.39,0.03) |
| Republic of the Gambia | -5 (-12,1) | -1.75 (-3.86,0.31) | -16 (-35,3) | -1.85 (-4.13,0.34) |
| Romania | -82 (-187,15) | -0.3 (-0.7,0.05) | -71 (-171,11) | -0.18 (-0.43,0.03) |
| Russian Federation | -721 (-1586,134) | -0.39 (-0.87,0.07) | -1157 (-2595,207) | -0.47 (-1.04,0.08) |
| Rwanda | -149 (-354,32) | -6.11 (-14.37,1.31) | -227 (-535,37) | -4.24 (-9.92,0.72) |
| Saint Kitts and Nevis | -4 (-8,1) | -8.84 (-18.62,1.62) | -5 (-12,1) | -9.55 (-20.54,1.6) |
| Saint Lucia | -7 (-14,1) | -8.13 (-17.41,1.53) | -16 (-35,3) | -6.87 (-15.08,1.15) |
| Saint Vincent and the Grenadines | -8 (-16,1) | -10.57 (-22.2,1.93) | -13 (-27,2) | -9.38 (-19.95,1.74) |
| Samoa | -2 (-5,0) | -3.21 (-7.23,0.59) | -3 (-7,1) | -2.6 (-6.02,0.53) |
| Sao Tome and Principe | -3 (-6,0) | -4.36 (-9.93,0.81) | -5 (-10,1) | -5.27 (-11.55,1.01) |
| Saudi Arabia | -30 (-84,4) | -0.68 (-1.87,0.1) | -57 (-178,9) | -0.49 (-1.47,0.08) |
| Senegal | -162 (-384,29) | -5.77 (-13.76,1.04) | -511 (-1186,105) | -7.74 (-17.86,1.6) |
| Serbia | -82 (-199,13) | -0.93 (-2.25,0.15) | -141 (-342,27) | -0.78 (-1.89,0.15) |
| Seychelles | -4 (-10,1) | -6.14 (-17.64,1.11) | -7 (-15,1) | -6.53 (-14.25,1.18) |
| Sierra Leone | -119 (-286,20) | -6.34 (-15.47,1.05) | -280 (-681,53) | -8.79 (-21.15,1.68) |
| Singapore | -13 (-28,2) | -0.68 (-1.49,0.13) | -28 (-63,5) | -0.34 (-0.76,0.06) |
| Slovakia | -82 (-189,13) | -1.34 (-3.12,0.22) | -122 (-284,22) | -1.22 (-2.85,0.23) |
| Slovenia | -11 (-25,2) | -0.43 (-0.99,0.08) | -17 (-42,3) | -0.34 (-0.84,0.06) |
| Socialist Republic of Viet Nam | -311 (-716,52) | -0.82 (-1.88,0.14) | -770 (-1867,164) | -0.86 (-2.1,0.18) |
| Solomon Islands | -5 (-13,1) | -5.61 (-13.78,0.76) | -14 (-33,2) | -5.43 (-12.73,0.86) |
| Somalia | -128 (-348,21) | -7.08 (-18.93,1.21) | -293 (-745,44) | -5.79 (-14.41,0.85) |
| South Africa | -1263 (-3082,183) | -6.81 (-16.54,0.99) | -3399 (-7545,631) | -7.99 (-17.69,1.5) |
| South Sudan | -142 (-361,29) | -5.86 (-14.87,1.2) | -155 (-392,29) | -5.08 (-12.65,0.96) |
| Spain | -445 (-1036,83) | -0.77 (-1.81,0.14) | -582 (-1354,99) | -0.51 (-1.2,0.09) |
| Sri Lanka | -175 (-413,31) | -1.94 (-4.53,0.35) | -322 (-756,54) | -1.21 (-2.83,0.2) |
| Sudan | -135 (-315,22) | -1.71 (-3.92,0.26) | -292 (-688,56) | -1.86 (-4.4,0.36) |
| Suriname | -11 (-28,2) | -5 (-12.32,0.85) | -33 (-77,6) | -5.48 (-12.82,0.94) |
| Sweden | -63 (-150,11) | -0.38 (-0.89,0.07) | -59 (-135,10) | -0.23 (-0.54,0.04) |
| Switzerland | -47 (-107,8) | -0.41 (-0.93,0.07) | -48 (-113,7) | -0.23 (-0.54,0.04) |
| Syrian Arab Republic | -50 (-120,8) | -1.16 (-2.96,0.19) | -131 (-316,23) | -1.31 (-3.15,0.23) |
| Taiwan (Province of China) | -73 (-157,14) | -0.55 (-1.18,0.1) | -301 (-660,53) | -0.69 (-1.52,0.12) |
| Tajikistan | -29 (-67,5) | -1.13 (-2.57,0.19) | -49 (-118,8) | -0.92 (-2.16,0.16) |
| Thailand | -936 (-1994,196) | -3.08 (-6.53,0.63) | -2636 (-6219,478) | -2.42 (-5.7,0.44) |
| The former Yugoslav Republic of Macedonia | -18 (-43,3) | -1.08 (-2.65,0.18) | -19 (-46,3) | -0.59 (-1.43,0.1) |
| Timor-Leste | -5 (-13,1) | -2.69 (-6.85,0.33) | -25 (-59,4) | -3.31 (-7.64,0.57) |
| Togo | -62 (-149,10) | -6.2 (-14.83,1.05) | -251 (-558,53) | -8.1 (-18.31,1.7) |
| Tokelau | 0 (0,0) | -6.77 (-16.08,0.95) | 0 (0,0) | -5.8 (-13.39,0.98) |
| Tonga | -4 (-10,1) | -8.68 (-20.18,1.35) | -6 (-14,1) | -7.91 (-18.2,1.46) |
| Trinidad and Tobago | -45 (-96,8) | -5.59 (-11.98,1.05) | -75 (-168,12) | -3.92 (-8.74,0.64) |
| Tunisia | -36 (-83,7) | -0.85 (-1.93,0.17) | -54 (-129,10) | -0.45 (-1.07,0.08) |
| Turkey | -188 (-443,38) | -0.67 (-1.57,0.13) | -358 (-881,67) | -0.42 (-1.02,0.08) |
| Turkmenistan | -6 (-14,1) | -0.35 (-0.8,0.07) | -7 (-16,1) | -0.18 (-0.43,0.03) |
| Tuvalu | 0 (-1,0) | -5.28 (-12.25,0.86) | -1 (-1,0) | -6.11 (-14.07,1.1) |
| Uganda | -561 (-1312,99) | -9.96 (-23.45,1.79) | -1207 (-2794,209) | -9.59 (-22.2,1.66) |
| Ukraine | -302 (-662,55) | -0.41 (-0.9,0.07) | -417 (-1004,77) | -0.51 (-1.24,0.09) |
| United Arab Emirates | -1 (-2,0) | -0.35 (-0.86,0.06) | -15 (-37,2) | -1.01 (-2.44,0.16) |
| United Kingdom of Great Britain and Northern Ireland | -601 (-1402,95) | -0.6 (-1.39,0.09) | -504 (-1207,89) | -0.34 (-0.82,0.06) |
| United Republic of Tanzania | -777 (-1944,136) | -7.89 (-19.67,1.4) | -1352 (-3351,224) | -6.09 (-15.06,1.04) |
| United States of America | -1818 (-4314,299) | -0.54 (-1.27,0.09) | -1626 (-3772,285) | -0.26 (-0.61,0.05) |
| United States Virgin Islands | -3 (-8,1) | -4.39 (-10.49,0.73) | -3 (-8,1) | -1.64 (-4.05,0.33) |
| Uruguay | -82 (-177,14) | -2 (-4.32,0.35) | -47 (-108,8) | -0.74 (-1.74,0.13) |
| Uzbekistan | -31 (-69,6) | -0.28 (-0.63,0.05) | -49 (-110,10) | -0.21 (-0.48,0.04) |
| Vanuatu | -3 (-7,0) | -6.86 (-15.99,1.05) | -9 (-20,2) | -6.8 (-15.65,1.12) |
| Yemen | -72 (-172,11) | -1.75 (-4.19,0.25) | -303 (-718,49) | -2.7 (-6.47,0.42) |
| Zambia | -246 (-563,55) | -10.21 (-23.95,2.31) | -1038 (-2726,124) | -16.94 (-43,2.19) |
| Zimbabwe | -506 (-1175,89) | -14.31 (-33.12,2.52) | -1017 (-2306,183) | -17.89 (-39.66,3.18) |
| DALYs, disability-adjusted life-years; ASDR, age-standardized DALY rate; UI, uncertainty interval. | | | | |

| Table S5. The deaths cases, age-standardized deaths, and temporal trends of colon and rectum cancer attributable to diet low in calcium in GBD regions, 1990 and 2021 | | | | | |
| --- | --- | --- | --- | --- | --- |
|  | 1990 | 1990 | 2021 | 2021 | 1990-2021 |
| Location | Deaths cases  No. (95% UI) | ASMR per 100,000  No. (95% UI) | Deaths cases  No. (95% UI) | ASMR per 100,000  No. (95% UI) | EAPC  No. (95% CI) |
| High SDI | 11445 (8119,15110) | 1.03 (0.73,1.36) | 15351 (10701,20424) | 0.66 (0.45,0.87) | -1.45 (-1.48 , -1.41) |
| High-middle SDI | 14059 (10251,17941) | 1.5 (1.09,1.91) | 18050 (12759,23869) | 0.92 (0.65,1.22) | -1.74 (-1.83 , -1.65) |
| Middle SDI | 20392 (15148,25325) | 2.14 (1.59,2.66) | 33662 (25076,42327) | 1.31 (0.97,1.65) | -1.72 (-1.78 , -1.66) |
| Low-middle SDI | 7334 (5375,9339) | 1.28 (0.96,1.63) | 14888 (11013,18210) | 1.1 (0.82,1.34) | -0.57 (-0.65 , -0.5) |
| Low SDI | 4074 (2951,5212) | 1.96 (1.45,2.47) | 7039 (5238,8698) | 1.59 (1.18,1.97) | -0.87 (-0.97 , -0.78) |
| Andean Latin America | 300 (219,388) | 1.57 (1.16,2.04) | 696 (478,936) | 1.21 (0.83,1.63) | -0.99 (-1.09 , -0.88) |
| Australasia | 247 (171,327) | 1.08 (0.75,1.42) | 361 (243,494) | 0.62 (0.42,0.85) | -1.99 (-2.15 , -1.83) |
| Caribbean | 404 (298,508) | 1.62 (1.21,2.03) | 787 (575,1022) | 1.46 (1.07,1.9) | -0.45 (-0.53 , -0.36) |
| Central Asia | 304 (221,387) | 0.67 (0.48,0.85) | 300 (209,398) | 0.4 (0.28,0.52) | -1.94 (-2.13 , -1.75) |
| Central Europe | 1530 (1074,2016) | 1.07 (0.75,1.41) | 2067 (1434,2743) | 0.89 (0.61,1.18) | -0.95 (-1.21 , -0.69) |
| Central Latin America | 629 (475,775) | 0.83 (0.63,1.03) | 1923 (1396,2465) | 0.79 (0.57,1.01) | -0.23 (-0.36 , -0.1) |
| Central Sub-Saharan Africa | 423 (304,554) | 2.18 (1.56,2.81) | 1094 (726,1576) | 2.29 (1.52,3.39) | 0.1 (0.04 , 0.15) |
| East Asia | 19435 (13891,25176) | 2.52 (1.79,3.24) | 22174 (15483,29949) | 1.07 (0.75,1.45) | -2.9 (-2.99 , -2.8) |
| Eastern Europe | 2929 (2109,3786) | 1.07 (0.77,1.38) | 3086 (2107,4050) | 0.86 (0.59,1.13) | -1.38 (-1.67 , -1.09) |
| Eastern Sub-Saharan Africa | 2219 (1563,2810) | 3.21 (2.28,4.05) | 3627 (2665,4547) | 2.56 (1.91,3.14) | -1.03 (-1.15 , -0.92) |
| High-income Asia Pacific | 2643 (1934,3365) | 1.39 (1.02,1.77) | 5967 (4201,7921) | 1.04 (0.74,1.36) | -0.8 (-0.88 , -0.73) |
| High-income North America | 3040 (2102,4076) | 0.84 (0.58,1.12) | 2972 (2039,4033) | 0.43 (0.3,0.59) | -2.01 (-2.11 , -1.91) |
| North Africa and Middle East | 1496 (1099,1907) | 0.98 (0.73,1.24) | 3086 (2209,3999) | 0.75 (0.53,0.96) | -0.77 (-0.86 , -0.69) |
| Oceania | 34 (24,45) | 1.36 (1.01,1.76) | 80 (57,100) | 1.18 (0.85,1.47) | -0.41 (-0.45 , -0.37) |
| South Asia | 4233 (3169,5448) | 0.79 (0.59,1.02) | 8376 (6120,10592) | 0.61 (0.45,0.77) | -0.88 (-0.96 , -0.8) |
| Southeast Asia | 8816 (6634,10796) | 3.64 (2.77,4.44) | 20991 (15708,26106) | 3.41 (2.56,4.23) | -0.32 (-0.4 , -0.24) |
| Southern Latin America | 646 (473,824) | 1.48 (1.08,1.88) | 1013 (724,1340) | 1.13 (0.81,1.5) | -0.46 (-0.63 , -0.3) |
| Southern Sub-Saharan Africa | 540 (411,717) | 2.19 (1.64,2.91) | 1383 (1054,1703) | 2.62 (1.97,3.22) | 0.57 (0.27 , 0.87) |
| Tropical Latin America | 745 (556,933) | 0.91 (0.67,1.14) | 1791 (1276,2335) | 0.71 (0.51,0.93) | -0.98 (-1.09 , -0.86) |
| Western Europe | 5562 (3881,7425) | 0.92 (0.65,1.23) | 5299 (3558,7159) | 0.48 (0.33,0.65) | -2.07 (-2.14 , -1.99) |
| Western Sub-Saharan Africa | 1186 (889,1488) | 1.52 (1.16,1.92) | 2014 (1447,2519) | 1.22 (0.87,1.49) | -0.77 (-0.82 , -0.71) |
| ASMR, age-standardized mortality rate; UI, uncertainty interval; EAPC, estimated annual percentage change; CI, confidence interval. | | | | | |

| Table S6. The DALYs cases, age-standardized DALYs, and temporal trends of colon and rectum cancer attributable to diet low in calcium in GBD regions,1990 and 2021 | | | | | |
| --- | --- | --- | --- | --- | --- |
|  | 1990 | 1990 | 2021 | 2021 | 1990-2021 |
| Location | DALYs  No. (95% UI) | ASDR per 100,000  No. (95% UI) | DALYs  No. (95% UI) | ASDR per 100,000  No. (95% UI) | EAPC  No. (95% CI) |
| High SDI | 235254 (167372,309405) | 21.54 (15.34,28.32) | 276182 (191267,367830) | 13.44 (9.33,17.88) | -1.48 (-1.52 , -1.45) |
| High-middle SDI | 361776 (263117,464840) | 35.97 (26.17,46.12) | 403351 (287676,533432) | 20.79 (14.83,27.52) | -1.98 (-2.07 , -1.89) |
| Middle SDI | 587646 (434963,736458) | 52.46 (38.97,65.36) | 860241 (640404,1080803) | 31.39 (23.35,39.44) | -1.8 (-1.86 , -1.74) |
| Low-middle SDI | 211529 (154145,268942) | 31.93 (23.29,40.75) | 397069 (291742,489556) | 26.36 (19.35,32.36) | -0.72 (-0.8 , -0.65) |
| Low SDI | 115089 (82738,146985) | 47.66 (34.46,60.97) | 189763 (140383,235803) | 35.54 (26.48,43.95) | -1.2 (-1.3 , -1.1) |
| Andean Latin America | 7287 (5299,9422) | 34.56 (25.21,44.85) | 15728 (10586,21443) | 26.36 (17.75,35.89) | -1.06 (-1.17 , -0.96) |
| Australasia | 5235 (3594,6927) | 22.65 (15.57,29.95) | 6665 (4495,9184) | 12.75 (8.58,17.42) | -2.09 (-2.25 , -1.93) |
| Caribbean | 9783 (7226,12441) | 37.18 (27.42,47.2) | 18308 (13185,24369) | 34.24 (24.61,45.57) | -0.36 (-0.44 , -0.29) |
| Central Asia | 8346 (6063,10715) | 17.09 (12.4,21.91) | 8006 (5482,10699) | 9.42 (6.49,12.56) | -2.31 (-2.53 , -2.09) |
| Central Europe | 33949 (23870,44795) | 22.97 (16.15,30.26) | 39956 (27821,53315) | 18.24 (12.66,24.39) | -1.05 (-1.31 , -0.79) |
| Central Latin America | 15637 (11833,19220) | 17.99 (13.58,22.13) | 46981 (34242,60391) | 18.49 (13.45,23.76) | 0.01 (-0.13 , 0.15) |
| Central Sub-Saharan Africa | 12169 (8770,16048) | 51.15 (36.79,66.95) | 31598 (20564,45047) | 52.97 (35.14,76.47) | 0.04 (-0.01 , 0.09) |
| East Asia | 561490 (402571,735058) | 60.93 (43.56,79.33) | 536168 (379605,725187) | 25.05 (17.63,33.9) | -3.01 (-3.11 , -2.92) |
| Eastern Europe | 70756 (50948,91823) | 25.33 (18.22,32.83) | 64953 (44555,85534) | 18.68 (12.79,24.61) | -1.75 (-2.07 , -1.43) |
| Eastern Sub-Saharan Africa | 62679 (43301,79618) | 78.22 (54.98,99.09) | 95984 (70422,123976) | 54.71 (40.17,68.74) | -1.52 (-1.66 , -1.39) |
| High-income Asia Pacific | 61786 (45437,78698) | 30.8 (22.66,39.2) | 98291 (70305,129309) | 21.6 (15.46,28.19) | -1.03 (-1.1 , -0.95) |
| High-income North America | 58581 (40963,78633) | 16.73 (11.68,22.4) | 58618 (40740,79326) | 9.43 (6.62,12.7) | -1.67 (-1.79 , -1.54) |
| North Africa and Middle East | 42058 (30089,53647) | 23.3 (16.91,29.73) | 82416 (58652,108454) | 17.06 (12.19,22.18) | -0.97 (-1.05 , -0.9) |
| Oceania | 1045 (750,1384) | 32.29 (22.88,42.42) | 2413 (1738,3087) | 28.53 (20.63,36.04) | -0.35 (-0.38 , -0.32) |
| South Asia | 123780 (92061,158662) | 19.66 (14.67,25.23) | 220553 (161659,280598) | 14.37 (10.57,18.28) | -1.06 (-1.14 , -0.99) |
| Southeast Asia | 256099 (191960,315041) | 91.45 (68.83,112.07) | 560315 (418460,695240) | 81.73 (61.48,101.51) | -0.49 (-0.57 , -0.41) |
| Southern Latin America | 13972 (10156,17951) | 30.55 (22.21,39.19) | 20622 (14724,27361) | 23.89 (17.1,31.67) | -0.37 (-0.52 , -0.22) |
| Southern Sub-Saharan Africa | 14175 (10755,18430) | 49.53 (37.61,65.11) | 36721 (28046,45674) | 60.37 (46.28,74.71) | 0.72 (0.4 , 1.04) |
| Tropical Latin America | 19081 (14358,23859) | 20.18 (15.13,25.21) | 43201 (30830,56259) | 16.66 (11.89,21.7) | -0.86 (-0.98 , -0.74) |
| Western Europe | 104399 (73127,139614) | 18.11 (12.7,24.23) | 89755 (61109,120994) | 9.61 (6.56,12.96) | -2.01 (-2.09 , -1.94) |
| Western Sub-Saharan Africa | 30455 (22721,38391) | 33.94 (25.36,42.62) | 51685 (36709,66205) | 25.6 (18.34,32.19) | -0.98 (-1.05 , -0.91) |
| DALYs, disability-adjusted life-years; ASDR, age-standardized DALY rate; UI, uncertainty interval; EAPC, estimated annual percentage change; CI, confidence interval. | | | | | |

| Table S7. The deaths cases, age-standardized deaths, and temporal trends of prostate cancer attributable to diet low in calcium in GBD regions, 1990 and 2021 | | | | |
| --- | --- | --- | --- | --- |
|  | 1990 | 1990 | 2021 | 2021 |
| Location | Deaths cases  No. (95% UI) | ASMR per 100,000  No. (95% UI) | Deaths cases  No. (95% UI) | ASMR per 100,000  No. (95% UI) |
| High SDI | -358 (-814,63) | -0.03 (-0.07,0.01) | -522 (-1183,92) | -0.02 (-0.05,0) |
| High-middle SDI | -350 (-782,64) | -0.04 (-0.09,0.01) | -591 (-1323,113) | -0.03 (-0.07,0.01) |
| Middle SDI | -784 (-1714,141) | -0.11 (-0.24,0.02) | -1837 (-3960,348) | -0.08 (-0.17,0.02) |
| Low-middle SDI | -587 (-1322,111) | -0.13 (-0.3,0.02) | -1363 (-2984,249) | -0.12 (-0.26,0.02) |
| Low SDI | -414 (-951,79) | -0.25 (-0.58,0.05) | -904 (-2032,175) | -0.25 (-0.55,0.05) |
| Andean Latin America | -46 (-99,8) | -0.27 (-0.58,0.05) | -104 (-229,19) | -0.19 (-0.41,0.03) |
| Australasia | -13 (-30,2) | -0.06 (-0.13,0.01) | -21 (-50,3) | -0.03 (-0.08,0.01) |
| Caribbean | -68 (-150,12) | -0.29 (-0.64,0.05) | -137 (-295,25) | -0.25 (-0.55,0.05) |
| Central Asia | -11 (-25,2) | -0.03 (-0.06,0) | -13 (-29,2) | -0.02 (-0.04,0) |
| Central Europe | -46 (-104,8) | -0.03 (-0.08,0.01) | -73 (-170,13) | -0.03 (-0.07,0.01) |
| Central Latin America | -117 (-247,22) | -0.18 (-0.37,0.03) | -301 (-646,53) | -0.13 (-0.28,0.02) |
| Central Sub-Saharan Africa | -52 (-118,10) | -0.4 (-0.91,0.08) | -141 (-327,26) | -0.41 (-0.95,0.07) |
| East Asia | -270 (-602,44) | -0.05 (-0.1,0.01) | -531 (-1239,98) | -0.03 (-0.06,0.01) |
| Eastern Europe | -52 (-116,10) | -0.02 (-0.04,0) | -87 (-192,16) | -0.02 (-0.05,0) |
| Eastern Sub-Saharan Africa | -163 (-382,30) | -0.29 (-0.68,0.05) | -337 (-788,56) | -0.27 (-0.62,0.05) |
| High-income Asia Pacific | -54 (-118,10) | -0.03 (-0.06,0.01) | -181 (-403,31) | -0.03 (-0.06,0) |
| High-income North America | -124 (-286,21) | -0.03 (-0.08,0.01) | -107 (-244,18) | -0.01 (-0.03,0) |
| North Africa and Middle East | -80 (-182,14) | -0.07 (-0.15,0.01) | -207 (-463,39) | -0.06 (-0.14,0.01) |
| Oceania | -5 (-12,1) | -0.29 (-0.69,0.04) | -16 (-38,2) | -0.35 (-0.82,0.04) |
| South Asia | -294 (-670,55) | -0.07 (-0.17,0.01) | -729 (-1693,121) | -0.06 (-0.15,0.01) |
| Southeast Asia | -299 (-661,57) | -0.15 (-0.34,0.03) | -839 (-1769,162) | -0.16 (-0.33,0.03) |
| Southern Latin America | -53 (-111,10) | -0.12 (-0.26,0.02) | -77 (-173,15) | -0.08 (-0.19,0.02) |
| Southern Sub-Saharan Africa | -99 (-231,15) | -0.45 (-1.05,0.07) | -228 (-496,43) | -0.49 (-1.05,0.09) |
| Tropical Latin America | -113 (-237,21) | -0.16 (-0.33,0.03) | -183 (-400,33) | -0.08 (-0.17,0.01) |
| Western Europe | -194 (-444,34) | -0.03 (-0.07,0.01) | -210 (-480,37) | -0.02 (-0.04,0) |
| Western Sub-Saharan Africa | -345 (-784,62) | -0.52 (-1.18,0.09) | -703 (-1611,146) | -0.52 (-1.19,0.11) |
| ASMR, age-standardized mortality rate; UI, uncertainty interval. | | | | |

| Table S8. The DALYs cases, age-standardized DALYs, and temporal trends of prostate cancer attributable to diet low in calcium in GBD regions, 1990 and 2021 | | | | |
| --- | --- | --- | --- | --- |
|  | 1990 | 1990 | 2021 | 2021 |
| Location | DALYs  No. (95% UI) | ASDR per 100,000  No. (95% UI) | DALYs  No. (95% UI) | ASDR per 100,000  No. (95% UI) |
| High SDI | -6247 (-14135,1115) | -0.54 (-1.22,0.1) | -8543 (-19103,1547) | -0.37 (-0.82,0.07) |
| High-middle SDI | -6645 (-14956,1215) | -0.71 (-1.6,0.13) | -10401 (-23450,2000) | -0.52 (-1.18,0.1) |
| Middle SDI | -15262 (-33261,2747) | -1.81 (-3.95,0.33) | -34282 (-73402,6497) | -1.37 (-2.94,0.26) |
| Low-middle SDI | -11540 (-26017,2193) | -2.28 (-5.12,0.43) | -25861 (-56832,4720) | -2.03 (-4.45,0.37) |
| Low SDI | -8647 (-19807,1658) | -4.56 (-10.43,0.86) | -18223 (-40992,3487) | -4.3 (-9.68,0.83) |
| Andean Latin America | -796 (-1740,146) | -4.38 (-9.59,0.81) | -1762 (-3872,326) | -3.12 (-6.85,0.58) |
| Australasia | -232 (-533,44) | -0.96 (-2.2,0.18) | -349 (-819,58) | -0.59 (-1.38,0.1) |
| Caribbean | -1224 (-2761,220) | -4.94 (-11.07,0.89) | -2422 (-5276,443) | -4.49 (-9.79,0.82) |
| Central Asia | -229 (-522,43) | -0.51 (-1.15,0.09) | -268 (-609,52) | -0.36 (-0.83,0.07) |
| Central Europe | -816 (-1836,148) | -0.56 (-1.26,0.1) | -1260 (-2922,228) | -0.52 (-1.22,0.09) |
| Central Latin America | -2160 (-4554,408) | -2.98 (-6.29,0.56) | -5466 (-11737,960) | -2.3 (-4.95,0.4) |
| Central Sub-Saharan Africa | -1097 (-2499,217) | -6.65 (-15.11,1.3) | -3005 (-6943,552) | -7.11 (-16.47,1.29) |
| East Asia | -5541 (-12330,907) | -0.77 (-1.7,0.13) | -9358 (-22053,1726) | -0.45 (-1.06,0.08) |
| Eastern Europe | -1103 (-2470,205) | -0.39 (-0.87,0.07) | -1724 (-3917,321) | -0.47 (-1.07,0.09) |
| Eastern Sub-Saharan Africa | -3498 (-8229,644) | -5.45 (-12.71,1.01) | -7215 (-16811,1162) | -5 (-11.64,0.83) |
| High-income Asia Pacific | -984 (-2129,179) | -0.51 (-1.1,0.09) | -2766 (-6098,486) | -0.49 (-1.09,0.09) |
| High-income North America | -2217 (-5170,373) | -0.59 (-1.39,0.1) | -1949 (-4490,348) | -0.28 (-0.64,0.05) |
| North Africa and Middle East | -1543 (-3514,277) | -1.13 (-2.56,0.2) | -3847 (-8691,722) | -1.03 (-2.32,0.19) |
| Oceania | -104 (-246,16) | -4.86 (-11.45,0.74) | -325 (-774,42) | -5.78 (-13.7,0.75) |
| South Asia | -5804 (-13251,1103) | -1.26 (-2.86,0.24) | -13260 (-30534,2198) | -1.02 (-2.37,0.17) |
| Southeast Asia | -6030 (-13195,1150) | -2.76 (-6.08,0.53) | -16744 (-35590,3213) | -2.85 (-6.05,0.55) |
| Southern Latin America | -927 (-1973,169) | -2.06 (-4.37,0.37) | -1271 (-2867,247) | -1.4 (-3.16,0.27) |
| Southern Sub-Saharan Africa | -1909 (-4501,296) | -7.91 (-18.62,1.23) | -4707 (-10386,879) | -8.98 (-19.64,1.69) |
| Tropical Latin America | -2133 (-4518,407) | -2.68 (-5.65,0.51) | -3158 (-6785,578) | -1.27 (-2.74,0.23) |
| Western Europe | -3231 (-7398,572) | -0.51 (-1.18,0.09) | -3263 (-7431,590) | -0.3 (-0.69,0.05) |
| Western Sub-Saharan Africa | -6840 (-15674,1238) | -9.03 (-20.58,1.64) | -13344 (-31106,2779) | -8.57 (-19.74,1.78) |
| DALYs, disability-adjusted life-years; ASDR, age-standardized DALY rate; UI, uncertainty interval. | | | | |
